# Genome sequencing of herb Tulsi (*Ocimum tenuiflorum*) unravels key genes behind its strong medicinal properties

**DOI:** 10.1186/s12870-015-0562-x

**Published:** 2015-08-28

**Authors:** Atul K. Upadhyay, Anita R. Chacko, A. Gandhimathi, Pritha Ghosh, K. Harini, Agnel P. Joseph, Adwait G. Joshi, Snehal D. Karpe, Swati Kaushik, Nagesh Kuravadi, Chandana S Lingu, J. Mahita, Ramya Malarini, Sony Malhotra, Manoharan Malini, Oommen K. Mathew, Eshita Mutt, Mahantesha Naika, Sathyanarayanan Nitish, Shaik Naseer Pasha, Upadhyayula S. Raghavender, Anantharamanan Rajamani, S Shilpa, Prashant N. Shingate, Heikham Russiachand Singh, Anshul Sukhwal, Margaret S. Sunitha, Manojkumar Sumathi, S. Ramaswamy, Malali Gowda, Ramanathan Sowdhamini

**Affiliations:** National Centre for Biological Sciences (TIFR), GKVK Campus, Bellary Road, 560 065 Bangalore, India; Centre for Cellular and Molecular Platforms, GKVK Campus, Bellary Road, 560 065 Bangalore, India; Manipal University, Madhav Nagar, 576104 Manipal, Karnataka India; School of Chemical and Biotechnology, SASTRA (A University), 613401 Thanjavur, TamilNadu India

**Keywords:** *O. tenuiflorum*, Basil, Thulasi, Genome, Transcriptome

## Abstract

**Background:**

Krishna Tulsi, a member of Lamiaceae family, is a herb well known for its spiritual, religious and medicinal importance in India. The common name of this plant is ‘Tulsi’ (or ‘Tulasi’ or ‘Thulasi’) and is considered sacred by Hindus. We present the draft genome of *Ocimum tenuiflurum* L (subtype Krishna Tulsi) in this report. The paired-end and mate-pair sequence libraries were generated for the whole genome sequenced with the Illumina Hiseq 1000, resulting in an assembled genome of 374 Mb, with a genome coverage of 61 % (612 Mb estimated genome size). We have also studied transcriptomes (RNA-Seq) of two subtypes of *O. tenuiflorum,* Krishna and Rama Tulsi and report the relative expression of genes in both the varieties.

**Results:**

The pathways leading to the production of medicinally-important specialized metabolites have been studied in detail, in relation to similar pathways in *Arabidopsis thaliana* and other plants. Expression levels of anthocyanin biosynthesis-related genes in leaf samples of Krishna Tulsi were observed to be relatively high, explaining the purple colouration of Krishna Tulsi leaves. The expression of six important genes identified from genome data were validated by performing q-RT-PCR in different tissues of five different species, which shows the high extent of urosolic acid-producing genes in young leaves of the Rama subtype*.* In addition, the presence of eugenol and ursolic acid, implied as potential drugs in the cure of many diseases including cancer was confirmed using mass spectrometry.

**Conclusions:**

The availability of the whole genome of *O.tenuiflorum* and our sequence analysis suggests that small amino acid changes at the functional sites of genes involved in metabolite synthesis pathways confer special medicinal properties to this herb.

**Electronic supplementary material:**

The online version of this article (doi:10.1186/s12870-015-0562-x) contains supplementary material, which is available to authorized users.

## Background

Plants of the genus *Ocimum* belong to the family Lamiaceae (Order Lamiales) and are widely distributed in the tropical, sub-tropical and warm temperate regions of the world [1]. These plants are known to produce essential oils comprising of a number of aromatic compounds and Tulsi is rightly known as the “Queen of Herbs” for this reason. In India, these plants are mostly grown at homes for worship and as offerings in temples. Among plants with medicinal value, those belonging to the genus *Ocimum* are very important aromatic herbs or shrubs.

**Fig. 1:**
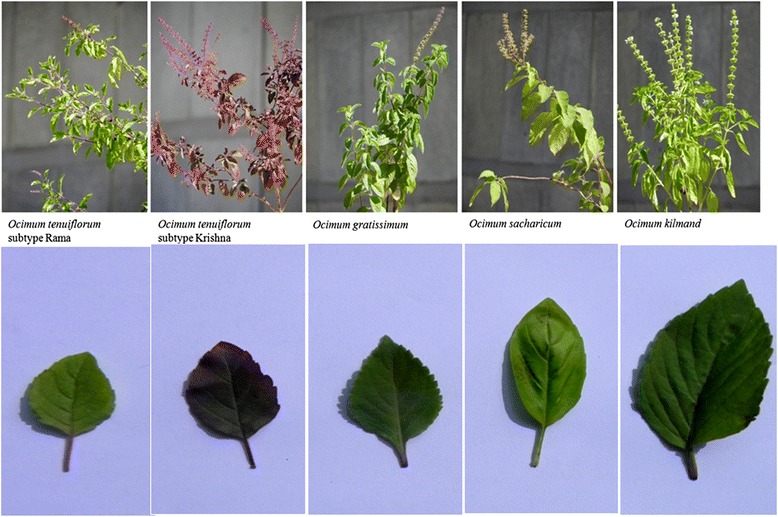
Plant and leaf morphology of five *Ocimum* species prevalent in India viz., *O. tenuiflorum* subtype Krishna, *O. tenuiflorum* subtype Rama, *O. gratissimum*, *O. sacharicum*, *O. kilmand.* Leaf morphologies are quite different for the five species

The genus *Ocimum* is highly variable and possesses wide genetic diversity at intra and inter-species levels. Nine species of *Ocimum* viz., *O. teniuflorum* L., *O. basilicum* L., *O. gratissimum* L., *O. kilimandscharicum, O. micranthum* L.*, O. campechianum* L., *O. americanum* L., *O. minimum* L., and *O. citriodorum* L., are found in India, three of which (*O. americanum* L., *O. minimum* L., and *O. citriodorum* L.) are exotic [2]. It is difficult to distinguish all these species on the basis of leaf morphology alone (Fig. 1). The metabolites (essential oils) of genus *Ocimum* have been reported to possess antioxidant and antifungal properties and to cure many diseases including bronchitis in Ayurveda, an Indian system of medicine [3]. Plants produce specialized metabolites as part of their defense mechanisms and these metabolites have significant medicinal properties that cure several human diseases. They can be isolated from various parts of the plant, including leaves, flowers, roots, bark, seeds and stem [4]. Pharmacological screening and the systematic study of the chemical constituents of plant metabolites provide a basis for developing new drugs. Some of the important metabolites reported from *Ocimum* species include linalool, linalyl, geraniol, citral, camphor, eugenol, methyleugenol, methyl chavicol, methyl cinnamate, thymol, safrol, taxol, urosolic acid etc. [4]. These metabolites are of immense value in the pharmaceutical, perfume and cosmetic industries. Metabolites derived from *Ocimum* species have been found to contain many medicinally relevant properties including anti-cancer, antioxidant, antifungal and anti-inflammatory virtues, and are also recommended for the treatment of malaria, bronchitis, diarrhea, dysentery, etc. [5]. Essential oils produced as specialized metabolites found in leaves, seeds, flowers and roots of *Ocimum* species are used in pharmaceutics and many systems of traditional Indian medicine [3, 4]. Genome and transcriptome sequencing of medicinal plants serve as a robust tool for gene discovery and downstream biochemical pathway discovery of medicinally important metabolites [6]. Recently, an abundance of transcripts for biosynthesis of terpenoids in *O. sanctum* and of phenylpropanoids in *O. basilicum* [7] was reported during an attempt to compare transcriptomes of the two species of *Ocimum*. Despite its important role in traditional Indian medicine and its impressive arsenal of bioactive compounds, our understanding of Krishna Tulsi biology is limited. In this paper, we present the draft genome sequence of the non-model plant *O. tenuiflorum* (subtype Krishna), along with transcriptomes of two subtypes, Krishna and Rama Tulsi from leaf samples. We have identified a large set of genes involved in the production of specialized metabolites of medicinal interest such as apigenin, luteolin, rosmarinic acid pathway, eugenol, and ursolic acid.

## Results

### Genome sequencing and assembly of the non-model plant *O. tenuiflorum* subtype Krishna

The paired-end (PE; 2x100-bp) and mate-paired (MP; 2x50-bp) DNA libraries were generated for Krishna Tulsi subtype using Illumina protocols. In total we obtained 373 million reads of PE and 166 million reads of MP data for Krishna Tulsi. Low quality (LQ) sequence reads were trimmed (Additional file 1: Figure S1 and Additional file 2: Figure S2) and reads with quality scores of less than Q30 were removed. The good quality reads were used for de-novo genome assembly. Median insert size of PE data was 335 (with a median absolute deviation of 21), whereas median insert size of MP data was 2473 (with a median absolute deviation of 704). K-mer 43 was opted as the best assembly from the statistical analysis of different k-mers. We obtained a maximum scaffold length of 184.7 Kb (Table 1) with an N50 length of 27.1 Kb. This assembly gives rise to a total of 78,224 scaffolds including equal to or more than 100 bp. The current draft assembly of Krishna Tulsi genome is 374.8 Mb in length. The genomic content of Krishna Tulsi is 0.72 pg/2C which is equivalent to 704.6 Mb [8], but the estimated genome size by k-mer method is 612 Mb and 61 % of the estimated genome size was assembled. The genome size reported in the literature [8], may be of a different cultivar. This lower genome coverage may be due to limited sequencing data (only two libraries were used in sequencing) or due to a high percentage of repeats (42.9 %). In terms of depth of sequencing, we sequenced 59× of the genome with paired-end (100 bp) and mate-pair (50 bp) libraries (since one lane can produce approximately 30Gb of data, even assuming that reads cover the entire 612 Mb of the estimated genome size). *Ocimum* species are characterized by the different basic chromosome numbers x = 8, 10, 12, or 16 [9, 10]. In case of *O. tenuiflorum* individuals with 2n = 32, 2n = 36, and 2n = 76 have been recorded and the chromosome number of *O. tenuiflorum* is observed to be 2n = 36 [8].

**Table 1:** Genome assembly results of Krishna Tulsi

Assembly	Status^a^	Number	N50 (bp)	Longest seq (*Kb*)	Size (*Mb*)	% GC
Contigs	All	510359	2562	37.147	362.4	53.3
Scaffold	All	78224	27,111	184.8	374.8	54.5

^a^Contigs/scaffolds equal or more than 100 bp

A comparative analysis of the assemblies generated using PE data alone and with both PE and MP data show that the size and quality of the genome assembled using PE data alone improved substantially with the inclusion of MP data (Additional file 3: Figures S3 and Additional file 4: Figure S4, Additional file 5: Table S1 and Additional file 6: Table S2).

### Validation of *de novo* genome assembly, annotation and repeat content of *Ocimum tenuiflorum* subtype Krishna genome

The *de novo* genome assembly was validated by mapping raw reads to the assembled genome. On an average, 74 % of reads were mapped back to the assembled genome. Almost 83.3 % of the RNA-seq reads were mapped to the assembled genome. The completeness of *de novo* genome assembly and annotations were also checked with two other approaches i.e., by using CEGMA (Core Eukaryotic Genes Mapping approach) [11] and DEG (Database of Essential Genes) [12] (please see Methods for details). First, we searched for essential eukaryotic genes in the *O. tenuiflorum* assembly. This resulted in the mapping of 85.1 % of complete core proteins (CEGMA) and more than 95 % including partial genes against our genome assembly (Additional file 7: Table S3). Secondly, we searched for the predicted genes from the final assembly of essential genes recorded in the DEG database. We observed that about 89 % of essential genes were included within the assembly. These genes were also validated using Pfam domain annotation and were of comparable domain lengths as the classical members of that family (Additional file 8: Table S4). Phylogenetic trees for highly conserved essential genes like glyceraldehyde 3-phosphate dehydrogenase (Additional file 9: Figure S5), cytochrome P450 (Additional file 10: Figure S6) and actin (Additional file 11: Figure S7) from Krishna Tulsi and their respective homologues were analyzed and compared with other plant species. Krishna Tulsi genes were found to cluster with genes belonging to related species namely, *Solanum lycopersicum, Cucumis sativus* and even with distantly related *Arabidopsis thaliana,* indicating that highly conserved genes, essential to plant growth and functioning, have been detected in *O. tenuiflorum* assemblies. These trends further support the quality of the genome assembly.

Regarding the repeat content of the genome, we identified 78224 repeat regions, with a GC content of 36.1 %, adding to 160889218 bp (160 Mb), which constituted 42.9 % of assembled genome which is 374806882 bp (374 Mb) long (Additional file 12: Table S5). Long terminal repeats (LTRs) are found in large numbers in plant genomes (Schmidt T**,** 1999) and a similar trend is also found in the type of repeats identified in the Tulsi genome.

### Genome annotation

We identified 36768 putative gene models in the initial genome draft (version 1.2) of *O. tenuiflorum* genome. At least one gene was observed in each of the 10012 scaffolds, with an average of three to four genes per scaffold. During the process of refined gene prediction, 16384 gene models were observed to have expression evidence (RNA-Seq data from leaves of Tulsi (Krishna and Rama)). A total of 19384 gene models have been identified by *ab initio* means (without any RNA or protein evidence) (Table 2).

**Table 2:** Genome annotation results of Krishna Tulsi

Annotation	Number	Average size (bp)	Total length (Mb)	% of genome	Transcript-evidence	*Ab initio*	Pfam hit
Gene	36768	2421	86.3	23	16384	19384	24,607

All the gene predictions, with or without RNA/protein evidences, were screened based on length (>100 bp). In case of sequential overlaps between different gene models, the gene models which are of longer length and with RNA or protein evidence for a given region of scaffold were preferred over the ones without any evidence.

There are 31,020 genes with at least one homologue in NRDB and 24,607 genes which contain at least one Pfam domain. In total, 3929 unique Pfam domains were identified for all the predicted genes in Tulsi (please see URL: http://caps.ncbs.res.in/Ote for the full list of predicted genes). A majority of domains identified were protein kinases or LRR-containing domains (Additional file 13: Figure S8). Further comparison of Pfam results, with assembled plant genomes of similar size, reveals that the number of predicted gene models is in overall agreement in numbers as well as gene boundaries.

### Orthology of Tulsi genes

**Fig. 2:**
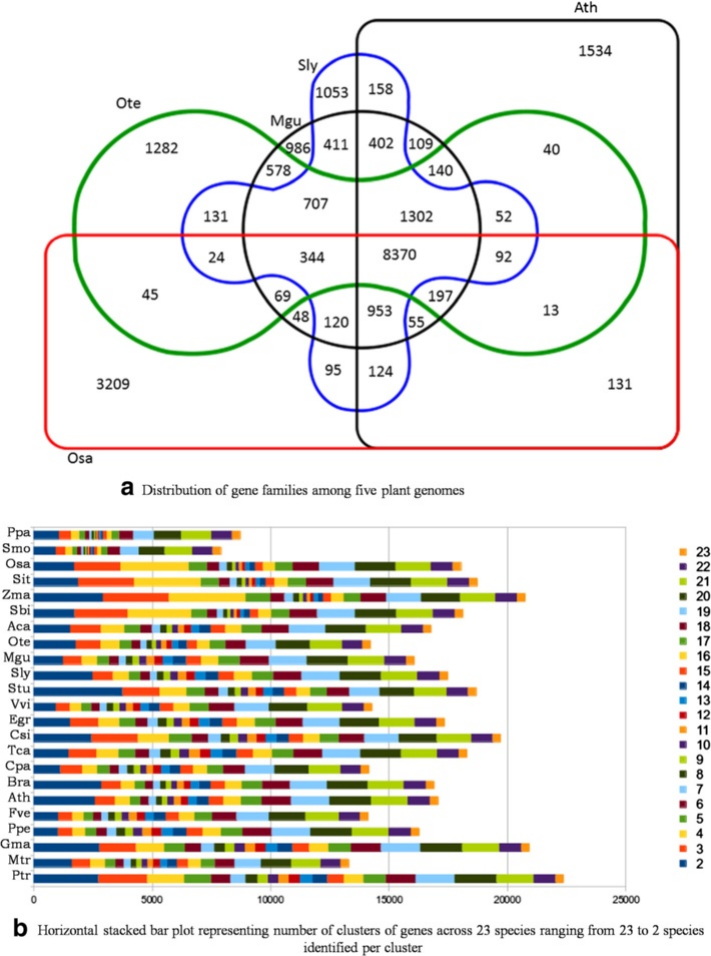
Distribution and clustering of orthologous genes of Tulsi genome to other related plant genomes. **a**. Distribution of gene families among five plant genomes. *Ocimum tenuiflorum* (Ote - green), *Arabidopsis thaliana* (Ath – black rectangle), *Oryza sativa* (Osa – red), *Solanum lycopersicum* (Sly – blue) and *Mimulus guttatus* (Mgu – black circle). The numbers in the Venn diagram represent shared and unique gene families across these 5 species obtained by OrthoMCL. **b**. Horizontal stacked bar plot of all the genes in 23 different genomes. This figure shows ortholog group distribution in all 23 plant species including Tulsi. Each row represents a plant species - *Physcomitrella patens* (Ppa), *Selaginella moellendorffii* (Smo)*, Oryza sativa* (Osa)*, Setaria italic* (Sit)*, Zea mays* (Zma)*, Sorghum bicolor* (Sbi)*, Aquilegia caerulea* (Aca)*, Ocimum tenuiflorum* (Ote)*, Mimulus guttatus* (Mgu)*, Solanum lycopersicum* (Sly)*, Solanum tuberosum* (Stu)*, Vitis vinifera* (Vvi)*, Eucalyptus grandis* (Egr)*, Citrus sinensis* (Csi)*, Theobroma cacao* (Tca)*, Carica papaya* (Cpa)*, Brassica rapa* (Bra)*, Arabidopsis thaliana* (Ath)*, Fragaria vesca* (Fve)*, Prunus persica* (Ppe)*, Glycine max* (Gma)*, Medicago truncatula* (Mtr)*, Populus trichocarpa* (Ptr). The bar graph represents ortholog protein groups for that species subdivided into 22 categories depending on the degree of sharing with the other 22 plant species e.g., category 2 represents the number of orthologous groups that have representatives from the species of interest and from one more species out of the 23 species selected for the study

The orthology relationships were deduced between Krishna Tulsi (*O. tenuiflorum*; Ote) and four other species viz. *Arabidopsis thaliana* (Ath), *Mimulus guttatus* (Mgu), *Solanum lycopersicum* (Sly) and *Oryza sativa* (Osa) (please see Methods for details). We observe 8370 clusters which contain a total of 89922 gene products from the five plant species (Fig. 2a). *M. guttatus* and *O. tenuiflorum* share the same order (Lamiales), but belong to different families (Phrymaceae and Lamiaceae, respectively), which was evident from the presence of the highest number of common gene families (11707) between them. This was followed by *Solanum lycopersicum* (11022), *Arabidopsis thaliana* (10206) and *Oryza sativa* (9154) as expected from taxonomic hierarchy (Fig. 2a). We found 17584 genes to be orthologous to any of the above four species. Considering all the 36768 Ote genes, 1282 groups contained only Ote Krishna Tulsi genes (3302). We obtained 16 Ote genes which lack traceable orthology to 22 other plant species and homology relationships (list of these genes is available on the database). Few of these unique Ote genes are transposons.

In order to inspect in detail the distribution of the orthologous relationship of *Ocimum* genes across different species and taxonomic levels, 22 fully-sequenced plant genomes (Additional file 14: Table S6) were considered. The orthologous groups from all 23 species were organized according to the clustering. Three hundred and thirty four clusters of genes are present across all the 23 species chosen for the study. Common genes across all species, comprising of their respective orthologous group, are plotted as a horizontal stacked bar plot (Fig. 2b). The pattern of sharing orthologous groups is quite unique to primitive plant genomes (like lycophyte and bryophyte) and monocots. However, the pattern observed in the Tulsi genome is quite similar to that of *M. guttatus* (Mgu). Interestingly, this pattern is somewhat different for two members of Solanacea, which have more genes shared only in two out of 23 genomes, perhaps due to other features such as polyploidy.

### Genes involved in synthesis of specialized metabolites of medicinal value: comparative analysis between *O. tenuiflorum* (Ote, Krishna Tulsi) and other plant genomes

**Fig. 3:**
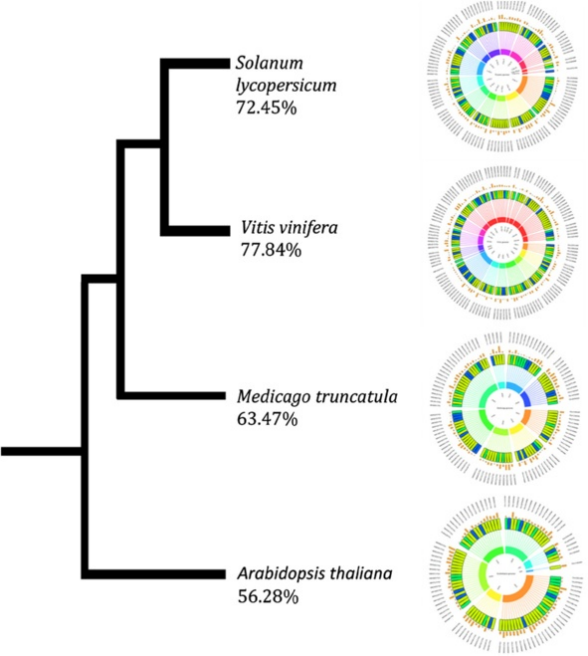
Phylogenetic representation of five selected plant genomes viz., *Solanum lycopercicum* (72.45 %), *Vitis vinifera* (77.84 %), *Medicago trucatula* (63.47 %), and *Arabidopsis thaliana* (56.28 %). The numbers indicate percentage of association of these genomes with the metabolite genes of *Ocimum* genome. These percentages agree with the taxonomic phylogeny and hierarchy, suggesting that the evolution of genes involved in metabolic pathways is not a cause of recent expansions or sudden genome drifts. The inner circle represents chromosomes from respective homolog genome. Each scaffold is organized in the middle circle and is represented in chronological order as per position on chromosomes. The line represents location of each scaffold on the respective chromosome. Colors indicate  = < 2 genes,  =2 genes,  = > 2 genes,  = Metabolite related genes. Height of orange columns in outermost circle represents amount of repeats in corresponding scaffolds

Next, we performed a restricted analysis of the genes involved in the metabolite production in Ote and the genomes of a few plant species that are either closely-related (*S. lycopersicum*, *V. vinifera)* or well-characterised (*M. truncatula*, and *A. thaliana*). We observed 121 (72.45 %), 130 (77.84 %), 106 (63.47 %) and 94 (56.28 %) scaffolds and contigs from the selected four representative genomes associated with 167 metabolite-related scaffolds and contigs in Ote Krishna Tulsi (Fig. 3) respectively*.* In terms of the number of orthologous genes from this selected plant genome associated with metabolite genes of Ote, we observed a similar trend of association as 601, 620, 570 and 556 genes in *S. lycopersicum*, *V. vinifera, M. truncatula*, and *A. thaliana* respectively. These numbers agree with the taxonomic phylogeny and hierarchy, suggesting that the evolution of genes involved in metabolic pathways is not a cause of recent expansions or sudden drifts.

**Fig. 4:**
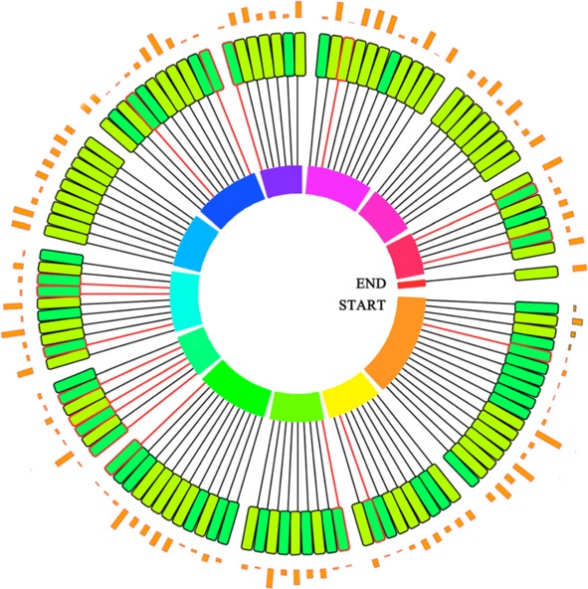
Circular representation of *O.tenuiflorum* metabolite related genes mapped onto chromosomes of *Solanum lycopersicum* genome. Height of orange column  in outer circle represents amount of repeats present in respective scaffold. The inner circle represents chromosomes from Tomato genome. Inner circle of rectangles represents scaffolds, each scaffold is organized in the middle circle and is represented in chronological order as per position on chromosomes. Color of each scaffold indicates following information:  =2 genes,  = > 2 genes,  = Metabolite related genes. Connecting line between scaffolds and chromosome represents postion of the scaffold in genome. Red color of connecting line represents presence of metabolite related genes. Scaffold Numbers are mentioned in Additional file 24: Text A

When compared against 11,389 scaffolds (greater than 10Kb in size) from Ote, 10032, 9997, 8648 and 8277 scaffolds were found to be associated with the four reference plant genomes (Additional file 15: Figure S9, Additional file 16: Figure S10 and Additional file 17: Figure S11 for three genomes and Additional file 18: Table S7 for four genomes). Further, most of the metabolite-related scaffolds in Ote Krishna Tulsi were associated with chromosomes 1, 6, 8, and 10 of tomato (Fig. 4). In particular, gene products that are likely associated in luteolin synthesis pathway are observed to cluster in scaffolds, which are similar to nucleotide stretches in Chromosomes 3, 5, 6, 8 and 10 of the tomato genome (Fig. 4).

### Transcriptome *de novo* assembly of Krishna and Rama Tulsi mature leaf samples

*De novo* transcriptome assembly was performed for the mature leaf samples of subtype Krishna Tulsi. The best assembly resulted in 109291 contigs with N50 of 893 bp and longest sequence of 12.1 Kb. All these contigs added up to 49.5 Mb with a GC content of 42.9 %. Scaffolding of these contigs resulted in 89878 scaffolds with N50 of 1597 bp and longest sequence of 12.7 Kb. All these scaffolds added up to 56.3 Mb with a GC content of 42.9 % (Table 3). Similarly, assembly was performed for the subtype Rama Tulsi and combined reads (Krishna and Rama Tulsi) as well (Table 3).

**Table 3:** Transcriptome assembly of *Ocimum tenuiflorum* subtype Krishna, Rama and combined data

	Assembly	Status^a^	Number	N50 (bp)	Longest seq (*Kb*)	Size (*Mb*)	% assembly
Krishna	Contigs	All	109291	893	12.1	49.5	42.9
	Scaffold	All	89878	1597	12.7	56.3	42.9
Rama	Contigs	All	108701	623	5.6	39.4	43.4
	Scaffolds	All	102038	815	6.4	40.5	43.4
Combined reads	Contigs	All	128469	742	12.2	54.4	42.9
	Scaffolds	All	115164	1073	12.2	57.1	42.9

^a^Contigs/scaffolds equal or more than 100 bp

### Differential expression of transcripts

The differentially expressed genes found in the transcriptomes of both the Tulsi subtypes were analysed. We observe a substantial number of genes up-regulated and down-regulated in Krishna Tulsi, compared to Rama Tulsi. Some of the highly expressed genes were also confirmed by q-RT-PCR technique in different tissue samples i.e., stems, leaves and flowers and also in five species viz. *O. tenuiflorum* subtype Krishna and Rama, *O. gratissimum*, *O. basilicum*, and *O. kilmand.*

**Fig. 5:**
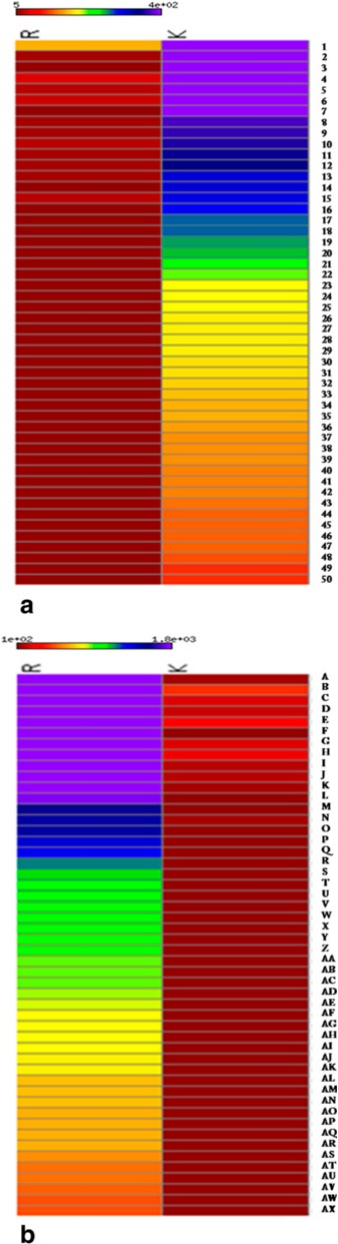
Transcript expression of Tulsi Krishna and Rama subtypes are expressed as RPKM values. Highly significant differentially abundant RNA scaffolds/transcripts were defined to have RPKM of atleast 5 in both and the fold-change difference between two subtypes should be atleast 8 times. Only the transcripts, for which the 95 % lower-confidence-bound of more abundant subtype and 95 % upper-confidence-bound of less abundant subtype, and had at least 8 times difference, were retained. Of these differentially abundant transcripts, top-50 in Krishna and Rama subtype were plotted in the form of heat-map. **a**. Differentially more abundant transcripts in Krishna. **b**. Differentially more abundant transcripts in Rama. (please look in Additional file 24: Text B and C for transcript IDs for a. and b)

For a comparison, we generated a heat map of the top 50 differentially more abundant genes in Krishna Tulsi samples (Fig. 5a). Similarly, top 50 differentially more abundant genes in Rama with respect to Krishna sample were also plotted (Fig. 5b). Gamma-cadinene synthase is one of the top 50 differentially expressed transcripts with RPKM values of 577.0 and 31.7 in Krishna and Rama Tulsi samples, respectively (please see below for details). Other highly expressed transcripts in Krishna Tulsi sample are Heat shock cognate protein 80, Cellulose synthase A catalyic subunit 6 (UDP-forming), Fructose-biphosphate aldolase (chloroplatic), Phototropin-2, and Rubisco activase 1 (chloroplatic). The chalcone synthase or naringenin-chalcone synthase (CHS) is one of the enzymes important for coloration of plant parts, which is observed to be highly expressed. Abundance values of all the transcripts, along with their functional annotations by NCBI BLAST results and their corresponding Krishna Tulsi genomic scaffold, show several genes involved in the synthesis of specialized metabolites implicated to be of medicinal value (Additional file 19: Table S8).

**Fig. 6:**
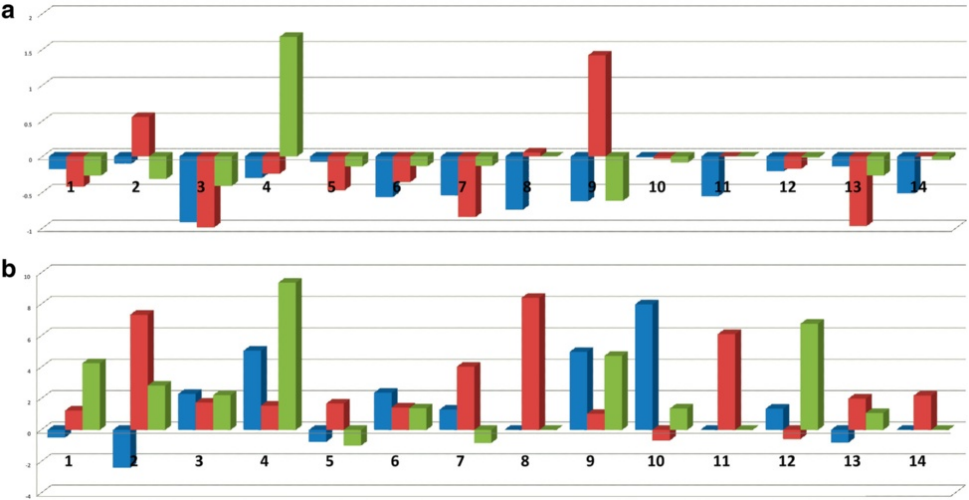
Expression quantification of selected genes by q-RT-PCR method. **a**. Fold changes of genes involved in color production, obtained through q-RT PCR. Blue colour horizontal bar is for chlorophyll a-b binding protein, red to denote Gamma-cadenine synthase and green to denote Anthocyanin. Mature leaf of Krishna subtype was used as control. It can be seen that, genes responsible for color production such as Chlorophyll a-b binding protein and gene in anthacyanin pathway are down-regulated as compared to mature Krishna leaf, which corresponds to phenotypic characteristics. **b**. Fold changes of genes involved in ursolic acid biosynthetic pathway, as obtained through qRT-PCR for 5 different Tulsi subtypes. Blue colour horizontal bar is for squalene epoxidase, red to denote alpha-amyrin synthase and green to denote Cytochrome P450 monooxygenase. Mature leaf of Krishna subtype was used as control. Mature leaf of Rama subtype has high expression of genes while expression in *Ocimum kilmund* is low. Expression of these genes are uniformly high in small, developing plants. Samples are as follows: 1) *O. tenuiflorum* (Rama) - Sampling Leaf. 2) *O. tenuiflorum* (Rama) - Sampling Root. 3) *O. tenuiflorum* (Rama) - Mature Leaf. 4) *O. tenuiflorum* (Krishna) - Sampling Leaf. 5) *O. tenuiflorum* (Krishna) - Sampling Root. 6) *O. gratissimum* - Sampling Leaf. 7) *O. gratissimum* - Sampling Root. 8) *O. gratissimum* - Mature Leaf. 9) *O. sacharicum* - Sampling Leaf. 10) *O. sacharicum* - Sampling Root. 11) *O. sacharicum* - Mature Leaf. 12) *O. kilmund* - Sampling Leaf. 13) *O. kilmund* - Sampling Root. 14) *O. kilmund* - Mature Leaf

Dark purple coloration of the leaves and stem of subtype Krishna Tulsi is one of its characteristic phenotypes, which distinguishes it from other subtypes and species of genus *Ocimum.* Chalcone synthase (CHS) is an enzyme belonging to a family of polyketide synthases which catalyzes the initial step for flavonoid biosynthesis. Flavonoids are important plant specific metabolites that perform various functions such as pigmentation, antifungal defense *etc*. Reviewed protein sequence for CHS from UniProt (Universal Protein resource) database [13] was employed to search against annotated protein sequences of Krishna Tulsi genome and six transcripts were obtained as possible hits. The best hit could be identified with 95 % query coverage and 99 % sequence identity. The extent of abundance of this hit (protein sequence) was checked in the leaf transcriptome of both the Tulsi subtypes viz. Krishna and Rama. Abundance (in terms of RPKM) of the six transcripts was, on an average, two times more in case of Krishna as compared to Rama (please see Fig. 5), and may be involved in the coloration phenotype of Krishna subtype plants [14]. For further confirmation of expression of these transcripts, q-RT-PCR was performed. As expected, anthocyanin producing gene was observed to be more abundant in Krishna young leaf samples and mature leaf samples (used as control) (Fig. 6a and b). In contrast, the chlorophyll binding protein was more abundant in Krishna mature leaf samples. In addition, we also examined the presence of gamma-cadeninene synthase gene which is responsible for aroma [15]. This gene was found to be more abundant in Rama root sample and young leaf samples of *O. Saccharum*, but not observed in higher quantities in *O. kilmund*.

### Specialized metabolites detection and validation

**Fig. 7:**
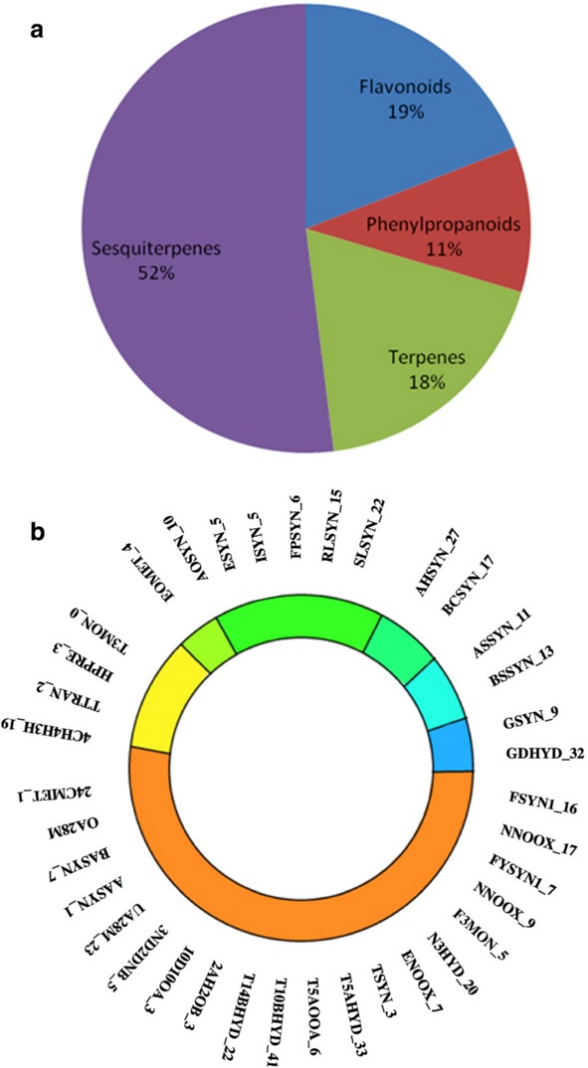
Number of genes involved in specialized metabolite synthesis in Tulsi genome. **a**. There are four classes of metabolites present in *Ocimum* genome viz., sesquiterpenes (52 %), flavonoids (19 %), terpenes (18 %) and phenylpropanoids (11 %). Number in the bracket is percentage of sepecialized metabolites present in the genome. 458 genes were identified as coding for enzymes involved in synthesis of specialized metabolites. **b**. Specialized metabolic pathways of disease relevance proposed in *Ocimum tenuiflorum.* Major classes of diseases investigated are indicated in different colors: anticancer , anticancer-antioxidant , antifungal , antiseptic , anti-infective , antioxidant , and anti-inflammatory . The enzymes have been labelled with 5–7 letters for convenience. The numbers after the’_’ in the enzyme label represent the number of putative hits found for the given enzyme in the genomic assembly of *O. tenuiflorum*. The metabolites involved in the disease relevance and the enzymes involved in the synthesis of these metabolites are as follows: APIGENIN (Flavone-synthaseI-FSYN1, Naringenin-NADPH-oxygen-oxidoreductase-NNOOX), LUTEOLIN (Flavone-synthaseI-FSYN1, Naringenin-NADPH-oxygen-oxidoreductase-NNOOX, Flavone-3-monooxygenase-F3MON), TAXOL (Taxadiene-synthase-TSYN, Taxadiene-5-alpha-hydroxylase-T5AHYD, Taxadien-5-alpha-ol-O-acetyltransferase-T5AOOA, Taxane-10-beta-hydroxylase-T10BHYD, Taxoid-14-beta-hydroxylase-T14BHYD, 2-alpha-hydroxytaxane-2-O-benzoyltransferase-2AH2OB, 10-deacetylbaccatin-III-10-O-acetyltransferase-10D10OA, 3-N-debenzoyl-2-deoxytaxol-N-benzoyltransferase-3ND2DNB, URSOLIC ACID (ursolic-aldehyde-28-monooxygenase-UA28M, Alpha-amyrin-synthase-AASYN), OLEANOLIC ACID (Beta-amyrin-synthase-BASYN, oleanolic-aldehyde-28-monooxygenase-OA28M), SITOSTEROL (24C-methyltransferase-24CMET), ROSMARINIC ACID I (4-coumaroyl-4-hydroxyphenyllactate-3-hydroxylase-4C4H3H, Tyrosine-transaminase-TTRAN), ROMARINIC ACID II (Hydroxyphenylpyruvate-reductase-HPPRE, Tyrosine-3-monooxygenase-TTRAN), METHYL CAHVICOL (Eugenol-o-methyltransferase-EOMET), EUGENOL (Alcohol-o-acetyltransferase-AOACE, Eugenol-synthase-ESYN, Isoeugenol-synthase-ISYN), LINALOOL (Farnesyl-pyrophosphate-synthase-FPSYN, R-linool-synthase-RLSYN, S-linool-synthase-SLSYN), CARYOPHYLENE (Alpha-humulene-synthase-AHSYN, Beta-caryophyllene-synthase-BCSYN), SELINENE (Alpha-selinene-synthase-ASSYN, Beta-selinene-synthase-BSSYN), CITRAL (Geraniol-synthase-GSYN, Geraniol-dehdrogenase-GDHYD)

Nearly 30 specialized metabolites (Fig. 7a) are reported form the genus *Ocimum* which are found to have medicinal values or properties [4]. Amongst these, 14 metabolites belonging to five basic groups were found to have complete pathway information in PlantCyc database (http://www.plantcyc.org/) [16] (Additional file 20: Figure S12). Hence, genes involved in these pathways were chosen for further analysis and searched against the assembled genome of *O. tenuiflorum*. Figure 7b highlights the distribution of the genes identified in various classes of metabolites of disease relevance (i.e., these metabolites are well-known as drugs in the cure of human diseases).

**Fig. 8:**
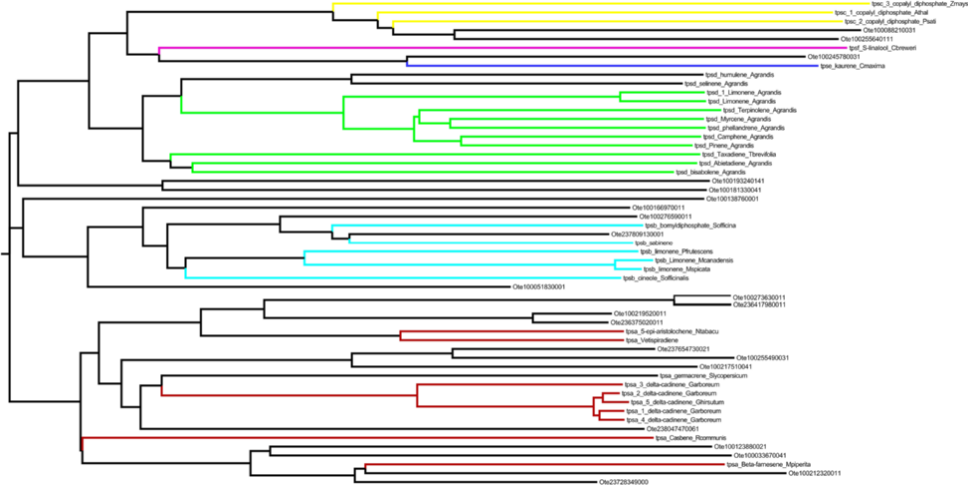
Phylogeny of terpene synthases of representative sequences of six classes from the plant kingdom along with putative Tulsi terpene synthases genes: The tree is color coded as tpsa:red, tbsb:blue, tpsc:yellow, tpsd: green, tpse: blue and tpsf:purple

A total of 458 genes were identified in Ote genome, which are either homologous or directly code for enzymes involved in the synthesis of specialized metabolites (Fig. 8) (details of gene IDs of these proteins are provided in Table 4 and Additional file 21: Table S9). Twenty eight *O. tenuiflorum* gene products were annotated as putative terpene synthases using BLAST sequence searches with E-value of 10^−4^ and query coverage filter of >75 % (Additional file 22: Table S10).

**Table 4:** The enzymes involved in metabolite biosynthesis were identified in the assembled genome and these genes were analyzed for their expression level in the transcriptome. The RKPM value signifies the level for expression

Sno.	Metabolite	Disease implication	Enzymes involved	No. of hits in *Ocimum* genome	RPKM value
	Flavonoids				
1	Apigenin	Anti-cancer	Flavone synthaseI	16	-
			Naringenin, NADPH oxygen oxidoreductase	17	-
2	Luteolin	Anti-cancer	Flavone synthaseI	7	34.44
			Naringenin, NADPH oxygen oxidoreductase	9	61.34
			Flavone 3’ monooxygenase	5	5.53
			Naringenin 3’ hydroxylase	26	104.24
			Eriodictoyl NADPH oxygen oxidoreductase	7	59.79
	Phenylpropanoids				
3	Rosmarinic acid Pathway1	Anti-cancer, anti-oxidant	4-coumaroyl-4’-hydroxyphenyllactate 3-hydroxylase	19	120.52
			Tyrosine_transaminase	2	0.00
4	Rosmarinic acid Pathway 2	Anti-cancer, anti-oxidant	Hydroxyphenylpyruvate_reductase	3	45.78
			tyrosine -3-monooxygenase	0	35.58
5	Eugenol	Anti-infective	Alcohol_o_acetyltransferase	10	4.69
			Eugenol synthase	5	68.93
			Isoeugenol synthase	5	68.93
6	Methylchavicol	Anti-fungal, antiparasitic, anti-oxidant	Eugenol-o-methyltransferase	4	132.39
	Terpenes				
7	Citral	Antiseptic	Geraniol synthase	9	0.00
			Geraniol_dehdrogenase	32	46.10
8	Linalool	Anti-infective	Farnesyl-pyrophosphate synthase	6	128.35
			r-linool_synthase	15	132.39
			s-linool_synthase	22	32.91
	Sesquiterpenes				
9	Caryophylene	Anti-inflammatory	Alpha humulene synthase	27	3.92
			Beta-caryophyllene synthase	17	3.92
10	Selinene	Anti-oxidant	Alpha_selinene_synthase	11	13.31
			Beta_selinene_synthase	13	
11	Taxol	Anti-cancer	Taxadiene synthase	3	9.36
			Taxadiene 5-alpha hydroxylase	33	3.43
			Taxadien-5-alpha-ol O-acetyltransferase	6	26.88
			Taxane 10-beta-hydroxylase	41	3.43
			Taxoid 14-beta-hydroxylase	22	3.43
			2-alpha-hydroxytaxane 2-O-benzoyltransferase	3	
			10-deacetylbaccatin III 10-O-acetyltransferase	3	
			3’-N-debenzoyl-2’-deoxytaxol N-benzoyltransferase	5	4.85
12	Ursolic acid	Anti-cancer	Cytochrome P450 monooxygenase	23	3.43
13	Oleanolic acid	Anti-cancer	Beta-amyrin synthase	7	12.69
			Cytochrome P450 monooxygenase	23	3.43
	Sterols				
14	Sitosterol	Anti-cancer	24C_methyltransferase	1	67.25

^-^refers to cases where there was no significant transcript evidence

**Fig. 9:**
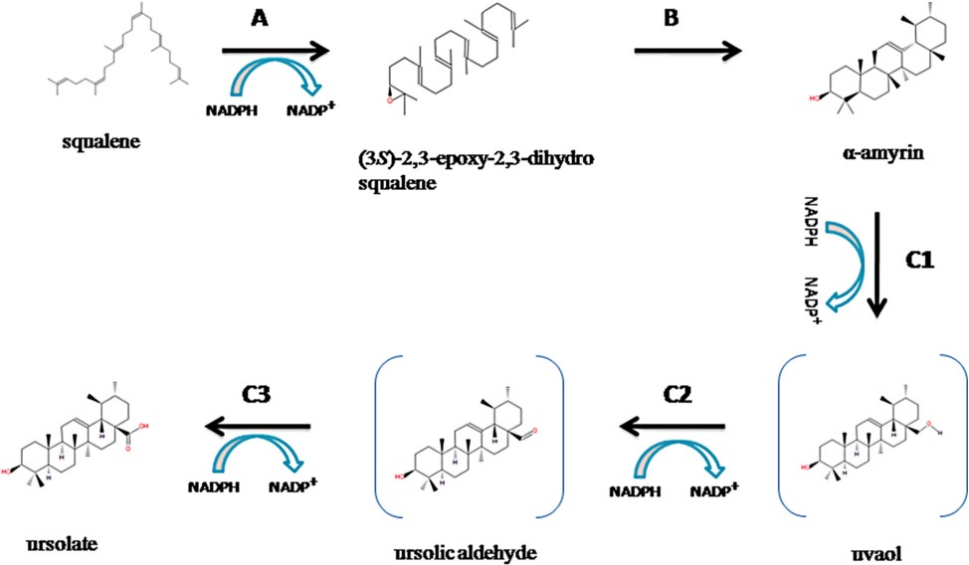
The synthesis of ursolic acid from squalene is a three-step process starting from squalene. A: Squalene epoxidase, B: α-amyrin synthase, C1: α-amyrin 28-monooxygenase [Multifunctional], C2: Uvaol dehydrogenase [Multifunctional] and C3: Ursolic aldehyde 28-monooxygenase. Squalene epoxidase and alpha amyrin synthase, along with alpha amyrin 28 mono-oxygenase, uvol dehydrogenase and ursolic aldehyde 28 mono-oxygenase, play important role in synthesis of ursolic acid. These three genes have been chosen for quantification of gene expression by q-RT PCR method in different tissues and species

**Fig. 10:**
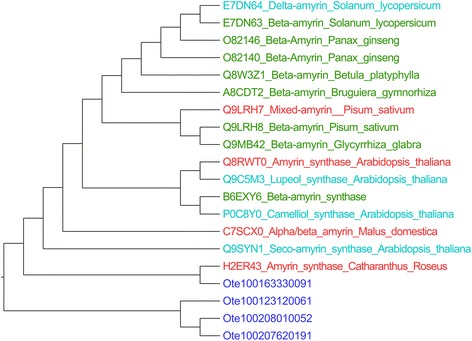
Phylogenetic tree of sixteen amyrin query sequences and four putative amyrins from Tulsi. Tulsi hits are marked in blue clour, red ones are alpha amyrin synthase, greens are beta amyrin synthase and cyan ones are proteins from other class of amyrin. The presence of motifs and position in the phylogeny indicate that the hits obtained in *O. tenuiflorum* genome are likely to be alpha-amyrin synthases

Among these specialized metabolites, we focused on ursolic acid, belonging to sesquiterpenes, since it is known to have anti-inflammatory, anti-microbial, anti-tumour and anti-cancer properties. The synthesis of ursolic acid from squalene is a three-step process starting from squalene (Fig. 9). α-Amyrin is formed by concerted cyclization of squalene epoxide, while ursolic acid is eventually synthesized by the catalytic activity of multifunctional cytochrome P450. The enzymes involved are, therefore, squalene epoxidase, alpha-amyrin synthase and alpha-amyrin 2, 8 monoxygenase. Sequence search algorithms were employed to search for the three enzymes of this pathway in the Tulsi genome, starting from protein sequences for each of these enzymes from PlantCyc database as queries. The search for squalene epoxidase in Tulsi, using the sequence of this enzyme in *Oryza sativa japonica* (LOC_Os02g04710.2) as a query, gave rise to a hit (C3776143), with 50 % sequence identity covering 80 % of the query length (Additional file 23: Figure S13). Using Amyrin synthase LUP2 from *A. thaliana* (Q8RWT0) and 13 other well-accepted alpha/beta amyrin synthases as a query, four hits were identified in the Tulsi genome (scaffold16333, scaffold20801, scaffold12312 and maker-C3776143). In classical amyrin synthases, a QW structural motif repeats six times in the entire sequence [17, 18], while there are two functional motifs, *viz*., a well conserved SDTAE [19] motif which is believed to form the catalytic pocket and the MWCYCR [20] motif that is shown to play a crucial role in catalysis. These motifs are observed in the four hits in Tulsi genome (Additional file 24: Text D). Further, a phylogenetic tree was constructed using 16 query sequences and these four hits (Fig. 10). One of the Tulsi hits, (scaffold 16333_mrnal) clusters with a well-characterized alpha amyrin synthase from *C. roseus* (H2ER439) suggesting that this particular scaffold might indeed retain an alpha amyrin synthase.

Interestingly, many genes involved in the synthesis of specialized metabolites of relevance in the treatment of diseases are also more abundant, as observed in the assembled transcriptome (Additional file 21: Table S9). Similarly, genes involved in the synthesis of 16 other specialized metabolites (Additional file 25: Table S11), are also equally interesting. However, this requires detailed understanding of the mechanism of synthesis and enzymes involved in the pathways. We analysed RNA-Seq data of two leaf samples in order to compare the genes related to important metabolite pathways and the peculiar phenotype of *O. tenuiflorum* subtype Krishna with subtype Rama Tulsi. There were 104 transcripts, whose fold change in expression was observed to be eight times more in Krishna Tulsi than in Rama Tulsi. Likewise, there were 229 transcripts whose fold change expression was eight times more in Rama Tulsi as compared to Krishna Tulsi. These are available for download at- (caps.ncbs.res.in/download/tdat_data/Supplementary_tables/Supplementary Table 8.txt).

In the case of the multifunctional Cytochome P450 (which catalyses the last three steps in the synthesis of urosolic acid, Fig. 9), a predicted gene from scaffold2032 was obtained as a hit, when a reviewed UniProt entry F1T282 from *V. vinifera* was considered as query and searched in the Tulsi genome assembly using BLAST. This hit retains 61 % sequence identity and the alignment covers 90 % of the length of the query (alignments are shown in Additional file 23: Figure S13). This scaffold contains a total of three predicted genes viz., Ote100020320011, Ote100020320001 (similar to UHRF1-binding protein) and Ote100020320031 (gene of interest).

From the available transcriptome assembly, these genes, identified as involved in the synthesis of urosolic acid, were analysed for their levels of expression. The RPKM values were also high for these three genes (please see Additional file 21: Table S9). To further validate the levels of expression of these genes, q-RT-PCR was performed using sequence-specific primers. The presence of these three enzymes is generally high in all the mature leaf samples and highest in Rama subtype (using Krishna subtype as control). Alpha-amyrin synthase is more abundant in mature leaf samples of *O. gratissimum* and *O. sacharicum* species. However, interestingly, the three enzymes are found to be more abundant in the young leaf samples of Rama subtype; in contrast, atleast one of the three genes is less in the Krishna leaf sample and in all root samples. The expression of the three genes implicated in urosolic acid synthesis is uniformly low in samples of *O. kilmund.*

Next, to correlate gene expression and to quantify the presence of ursolic acid and eugenol, chemical profiling was performed using LC-Mass spectrometry from different tissues and samples. Eugenol and ursolic acid were observed in the highest quantities in mature leaf sample of Rama subtype and in relatively low quantities in *O. kilmund*. The amount of eugenol in the leaf sample of *O. tenuiflorum* subtype Rama (2235 ng/mg) is considerably high followed by *O. kilmund* (1472 ng/mg), *O. sacharicum* (651 ng/mg) and lowest in *O. gratissimum* (73 ng/mg). In all stem samples, the amount of eugenol is consistently low with the highest in *O. tenuiflorum* subtype Rama (24 ng/mg), *O. tenuiflorum* subtype Krishna (17 ng/mg), *O. kilmund* (15 ng/mg) and below limits of quantification in *O. gratissimum* and *O. sacharicum*. The presence of oleanolic acid is also severely reduced in stem samples of Rama subtype (2869 ng/mg) and in Krishna subtype (1088 ng/mg) in comparison to the mature leaf samples (7556 ng/mg for Rama and 4630 ng/mg for Krishna). The presence of urosolic acid is 50 % less in stem samples of Rama subtype (2883 ng/mg) when compared to the mature leaf samples (4597), whereas it is much lower in the stem samples of other species as compared to the leaf sample. The amount of ursolic acid in the stem samples of Krishna subtype (746 ng/mg) is 4.6 times less than that of the mature leaf samples (3471 ng/mg) (please see Table 5).

**Table 5:** Estimation results of Eugenol and Ursolic acid in different samples

S. No	Sample	Eugenol (ng/mg) Leaf Stem		Ursolic acid (ng/mg) Leaf Stem		Oleanolic acid (ng/mg) Leaf Stem	
1	*O. tenuiflorum* (Rama Tulsi)	2235.93	24.91	4597.62	2883.46	7556.84	2869.80
2	*O. tenuiflorum* (Krishna Tulsi)	449.89	17.16	3471.59	746.02	4630.53	1088.21
3	*O. gratissimum*	73.88	BLQ	4391.97	1139.15	2584.56	711.08
4	*O. scharicum*	651.09	BLQ	4436.26	1491.68	2975.63	1024.14
5	*O. kilmand*	1472.51	15.74	1099.85	191.09	945.22	159.37

*• BLQ* Below level of quantification

## Discussion

*O. tenuiflorum* subtype Krishna Tulsi is one of the non-model plants of great medicinal value, for which there has been no genomic information available till date. We have performed genome sequencing of *O. tenuiflorum* subtype Krishna of the paired-end (PE; 2x100-bp) and mate-paired (MP; 2x50-bp) DNA libraries by Illumina Hiseq 1000. The best *de novo* assembly was obtained at k-mer 43 by SOAPdenovo2, an eukaryotic *de novo* genome assembler. Repeats were identified and masked, and gene prediction and annotation was carried out using the MAKER annotation pipeline by using genomic, transcriptomics and EST data. The nearest species whose genome has been sequenced is the monkey flower (*M. guttatus*), which shares its order Lamiales with *O. tenuiflorum* (Ote) but falls in a different family (Phrymaceae). Orthology search of Ote Krishna Tulsi genes in four genomes viz. *A. thaliana* (Ath), *M. guttatus* (Mgu), *S. lycopersicum* (Sly) and *O. sativa* (Osa) also confirmed the close relationship between Krishna Tulsi and *M. guttatus* (Mgu), in terms of the number of common gene families i.e., 578 out of 2488 total genes. When we considered all the 36,768 predicted genes from the Krishna Tulsi genome, we found that 1282 ortholog groups have *Ocimum-*only genes. These 1282 groups contain 13,306 *Ocimum* genes and hence they are referred to as paralogs by OrthoMCL. Of the remaining Ote genes, 17,584 genes were found to be orthologous to any of the other four species studied in this case. We performed an analysis of the genes involved in the metabolite production in *Ote* and the genomes of a few other related plant species. Based on the direct evidence or homology a total of 458 genes were identified in Ote genome, which are involved in coding of enzymes implied in the synthesis of specialized metabolites. Comparative analysis of transciptomes of *O. tenuiflorum* subtype Krishna and Rama was performed to detect potential differentially-regulated genes and their involvement in metabolite synthesis. On comparing both the transcriptomes, differentially expressed genes were observed with a substantial number of genes more abundant and others less abundant in either subtypes. Gamma-cadinene synthase is more abundant in Krishna sample (RPKM value 577.047) as compared to Rama sample (RPKM value 31.73). To confirm some of the more abundant genes along with Gamma-cadinene synthase, we performed q-RT-PCR in different tissue samples i.e., stem and leaves and also in five species viz. *O. tenuiflorum* subtype Krishna and Rama, *O. gratissimum*, *O. basilicum*, and *O. kilmand.* Expression of Gamma-cadinene synthase is found more in Krishna samples as compared to Rama by q-RT-PCR also. Likewise, Chalcone synthase (CHS) is an anthocyanin-producing gene, which is observed to be more abundant in young leaf samples of Krishna and mature leaf samples in transcriptome data. Subsequently, this has been confirmed by q-RT-PCR and from mass spectrometry readings of ursolic acid and eugenol from different tissue samples and from different species.

## Conclusion

We present a draft genome of *O. tenuiflorum* Krishna Tulsi subtype Krishna Tulsi. The habitat of genus *Ocimum* is tropical climate and it is wide spread over Asia, Africa, Central and South America. High RNA-seq expression values of the genes responsible for the purple coloration of the plant parts in Krishna subtype, as compared to Rama subtype, were observed. We also identified a fFew unique genes (16) of Ote, which lack any traceable orthology and homology relationships from all the 22 species used in this study.

Krishna Tulsi is described in the Vedas and Puranas (ancient scriptures of Hindus) and has a long history of cultivation, of roughly 3000 years, and is therefore assumed to be of Indian origin [21]. In literature, it is also referred to as the “Queen of Herbs”. Major genes involved in the synthesis of medicinally important specialized metabolites in the plant could be unraveled despite limited data on sequencing and coverage [22]. Expressions of these genes were confirmed by complementing with RNA-seq data and q-RT-PCR method. We also investigated one of the important metabolic pathways involving the production of ursolic acid in detail, by mass-spectrometry and q-RT-PCR methods. Synthesis of specialized metabolites or their precursors appear to begin in the young leaves of Tulsi. Subsequently, the mature leaves retain the medicinally relevant metabolites. *O. tenuiflorum* Rama subtype retains the high abundance of key medicinally relevant metabolites like eugenol and ursolic acid, as observed in the transcriptome, metabolite quantifications and q-RT-PCR expression values consistent with its high medicinal values. Our main emphasis was to unravel the important metabolite genes by using genomic and transcriptomic data despite limited sequencing information.

## Methods

### Isolation of genomic DNA from *O. tenuiflorum* subtype Krishna Tulsi

Young leaves of Tulsi subtype Krishna and Rama were used for genomic DNA isolation. About one gram of leaves were crushed using liquid Nitrogen and DNA extraction buffer (200 mM TrisHCL [pH-8.0], 200 mM NaCl, 25 mM EDTA and 1 % PVP) was added [23]. The ground material along with 1/10th volume of 20 % SDS solution was incubated at 65 °C for 30 min. The tubes were centrifuged at 14,000 RPM for 10 min at room temperature to remove the debris. The supernatant was transferred into a fresh tube and treated with equal volume of phenol: chloroform: isoamyl alcohol (25:24:1) and mixed gently for 5 min. The mixture was centrifuged at 12,000 RPM for 10 min to separate the phases. The aqueous phase from the centrifuged tube was transferred to a fresh tube and DNA was precipitated with 1/5th volume of 2 M NaCl and 2 volumes of ice-cold ethanol. The DNA was pelleted by centrifugation at 12,000 RPM for 10 min. Precipitated DNA pellet was taken as a starting material for purification using the Sigma Genelute plant DNA isolation kit (G2N70, Sigma). The DNA was run on a 1 % agarose gel to assess the quality. The A260/280 ratio and quantity were determined using the nanodrop.

### Genome sequencing, assembly and annotation

Genome sequencing was performed by using Illumina HiSeq 1000 technology in the Next Generation Genomics Facility at Centre for Cellular and Molecular Platforms (C-CAMP). Genomic DNA paired-end and gel free mate-pair library preparation was performed for Krishna Tulsi using TruSeq DNA sample preparation kit (FC-121-2001) and Nextera mate-pair sample preparation kit (FC-132-1001) from Illumina (www.illumina.com). FASTX-Toolkit [24] and FastQC tools [25] were used for pre-processing of raw reads and for quality check of the reads. Genome assembly from reads of PE and MP together was done by using SOAPdenovo2, a *de novo* draft genome assembler [26]. Preliminary assemblies were performed based on k-mers from 21 to 63 with an interval of two. Gene prediction and annotation was carried out using the MAKER annotation pipeline [27] with predicted gene models using AUGUSTUS [28] and *A. thaliana* genes as reference for initial prediction. The gene models were refined using homology searches against all protein sequences from *Viridaeplantae* kingdom.

### Validation of genome assembly and annotations

To validate genome assembly, we have mapped raw reads on to the *de novo* assembled genome by using REAPR (SMALT) [29], SAMtools [30] and Picard tools (http://broadinstitute.github.io/picard/). Maximum and minimum insert size of 500 bp and 0 bp respectively were selected for mapping. We report an alignment pairing with best score, using standard Smith-Waterman scores. The threshold minimum score used was calculated by the formula to be: < Minimum score > = < world length > + step size – 1. Here the word length of 13 is used with a step size of 6. Estimation of the genome size of the Tulsi genome was done using the k-mer distribution analysis by Jellyfish [31]. Essential genes implicated in the regulation, assembly and functioning of plant cells, have been identified in the Krishna Tulsi assembled genome using a two-way approach. Firstly, using CEGMA which was derived from the KOG database [32] (for eukaryotic genomes) and core proteins in any eukaryotic genome (including ones in draft stages), essential genes were annotated. Secondly, a subset of *A. thaliana* genes were extracted from a well-characterized Database of Essential Genes (DEG) and compared against Krishna Tulsi assemblies. Validation of the extracted genes was performed by Pfam domain annotation approaches. Putative essential genes from the Krishna Tulsi dataset were further searched using BLASTP [33] against the NCBI (NR) database and closely-related homologues were aligned and phylogenetic tree constructed.

### Repeat identification

Repeat elements in the assembled genome were identified using RepeatScout (version 1.0.5) [34] and RepeatMasker (version 4.0.3) [35]. The library of *ab initio* repeats generated by RepeatScout was classified into known repeat classes using the RepeatClassifier module of RepeatScout (Additional file 12: Table S5). The RepBase library of RepeatMasker and the non-redundant library of *ab-initio* classified repeats were then used to mask the repeat elements in the assembled genome. The repeat-masked genome assembly was then used for genome annotation.

### Genome annotation

The repeat-masked assembled genome of Krishna Tulsi was processed through the MAKER annotation pipeline [27]. AUGUSTUS [28] was used for gene prediction, trained on *A. thaliana* gene models. RNA-seq data obtained from leaf samples was used as EST evidence to refine the gene models. Initial gene models of protein sequences belonging to *Viridaeplantae* kingdom, obtained from the NCBI database, were used as protein evidence for refining gene prediction. Both EST and protein evidence were prepared using EXONERATE [36] and used for gene prediction refinement through AUGUSTUS. All the protein sequences of these gene models were subjected to validation based on identification of homologues through BLASTP search against NRDB at E-value cutoff of 10^−3^. Pfam release 27 was consulted for all domain predictions with an E-value cutoff of 10^−5^ using HMMER3 package [37].

### Orthology detection

All the predicted gene models from Krishna Tulsi were used with OrthoMCL tool [38] to identify clusters between selected species of *A. thaliana* (Ath), *O. sativa* (Osa), *S. lycopersicum* (Sly), *M. guttatus* (Mgu). In order to inspect distribution of the orthologous relationship of *Ocimum* genes across different species and taxonomic levels, ProteinOrtho tool [39] was implemented on Krishna Tulsi (Ote) gene models along with 22 different species: *Aquilegia caerulea* (Aca), *Glycine max* (Gma), *Setaria italic* (Sit), *Mimulus guttatus* (Mgu), *Solanum lycopersicum* (Sly), *Arabidopsis thaliana* (Ath), *Medicago truncatula* (Mtr), *Selaginella moellendorffii* (Smo), *Brassica rapa* (Bra), *Oryza sativa* (Osa), *Solanum tuberosum* (Stu), *Carica papaya* (Cpa), *Physcomitrella patens* (Ppa), *Theobroma cacao* (Tca), *Camellia sinensis* (Csi), *Prunus persica* (Ppe), *Vitis vinifera* (Vvi), *Eucalyptus grandis* (Egr), *Populus trichocarpa* (Ptr), *Zea mays* (Zma), *Fragaria vesca* (Fve), *Sorghum bicolor* (Sbi). All the complete proteome sets were obtained from Phytozome resource [40]. Phylogenetic tree reconstruction was carried out using ‘RbcS’ (Rubisco small subunit) coding sequences from all 23 species. CLUSTALW [41] and Phylip package [42] were employed for multiple sequence alignment (MSA) and subsequent clustering using Neighbor Joining (NJ) method, respectively. Distant homology relationships were verified through PSI-BLAST [33] at different set of E-value cutoffs. Gene products for which we were unable to establish any homology or orthology relationships, but consisted of a Pfam domain, were referred to as unique genes specific to Ote.

### Comparative analysis between Krishna Tulsi and other plant genomes

The most recent version of whole genome sequences of *S. lycopersicum, V. vinefera, M. tranculata* and *A. thaliana* were downloaded from NCBI (ftp://ftp.ncbi.nlm.nih.gov/genomes/). BLAT [43] was employed for sequence searches using *S. lycopersicum, V. vinefera, M. tranculata* and *A. thaliana* genomes against two sets of Tulsi genome data: one containing 11389 scaffolds (which are greater than 10000 bp) and another containing 167 scaffolds and contigs with metabolite-related genes (identified earlier on the Krishna Tulsi genome). The figures were prepared using in-house software written for this purpose.

### Isolation of RNA from Tulsi subtypes, Krishna and Rama, and RNA-seq library preparation

RNA isolation was carried out with 100 mg of the leaf tissue (Rama and Krishna) using the Sigma Spectrum Plant Total RNA Kit (STRN50, Sigma). DNA contamination was removed by DNAse treatment using DNA-free™ kit (AM1906, Ambion). The DNase free RNA quality was determined using the Agilent Bioanalyzer. The RNA Integrity Number (RIN) values of all the samples were greater than 6. The A260/280 ratio and the quantity were determined using the nanodrop. RNA-seq library preparation was done with 1 μg of total RNA following the TruSeq RNA sample preparation from Illumina (RS-122-2001).

### Transcriptome sequencing and assembly

We assembled all the mRNA reads having HQ scores of all the bases more than 20, of Krishna and Rama subtype separately and also by combining the reads from both of these subtypes by using SOAPdenovo-trans [26] at different K-mers starting from 19 to 63 at an interval of two. An insert size of 350 was used for the assembly of transcriptomes. RNA-seq reads were mapped to the assembled genome by Tophat2 [44], which uses Bowtie2 [45] as a mapping tool. We used a minimum and maximum intron length of 50 and 500000 bp respectively. Maximum multi hits (parameter that dictates the number of alignments to the reference for a given read) was assigned as 20 and transcriptome max hits (maximum number of mappings allowed for a read, when aligned to the transcriptome) of 60 was used.

### Transcript differential expression comparison

To quantify expression in terms of reads per kilo base per million (RPKM), non-redundant combined assembled transcript sequences (at 90 % sequence similarity by CD-hit EST [46]) were taken as reference. This non-redundant transcriptome was used as the reference transcriptome to calculate differential expression of transcripts in both the samples [6, 47]. The reads of RNA-seq experiments from Krishna and Rama subtypes were mapped back on to the reference transcriptome by using SeqMap (version – 1.0.12) [48] and RPKM values were determined by using rSeq: RNA-seq analyzer (version 0.1.1) [49].

### Specialized metabolites detection and validation

The dataset obtained after gene prediction on the assembled genome was employed to search for enzymes involved in secondary metabolite production. There are 14 metabolites (flavonoids (2), phenylpropanoids (4), terpenes (2), sesquiterpenes (5) and sterols (1)), which are reported to be present in *Ocimum* and have known pathway information in PlantCyc (http://www.plantcyc.org/) [16]. Reviewed entries from the UniProt database and all the known sequences of the enzymes from other species possessing these enzymes were used as queries to search in the full dataset of scaffolds and contigs, using PSI-BLAST at E-value of 10^−5^ and three iterations. The protein hits obtained in our dataset were further subjected to validation using a query coverage filter of 75 %.

In order to study the expression of genes involved in the synthesis of specialized metabolite (s), the assembled transcriptome of both *Ocimum* species were searched, employing the reviewed entry corresponding to each enzyme in the UniProt database. These searches were performed using TBLASTN at an E-value of 10^−3^, and the best hit in our dataset was selected based on the least E-value. If the reviewed entry for any of the enzyme was not present, unreviewed entries from PlantCyc database were employed.

### Quantification of eugenol and ursolic acid using UHPLC-MS/SRM method

A Vantage TSQ triple stage quadrupole mass spectrometer (Thermo Fisher Scientific, San Jose, CA, USA) equipped with a heated electro spray ionization (HESI) source was used for the analysis of eugenol and an APCI probe was used for the ursolic acid analysis. The mass spectrometer was interfaced with an Agilent 1290 infinity UHPLC system (Agilent Technologies India Pvt. Ltd., India) equipped with a column oven (set at 40 °C), auto sampler and a thermo-controller (set at 4 °C). The needle was washed from outside with acetonitrile (0.1 % formic acid) before every injection to avoid any potential carry-over problems. Separations were performed using a shim-pack XR-ODSIII column (2 × 150 mm, 2 μm). For Eugenol: Mobile phase A was water (10 mM Ammonium acetate) containing 0.1 % formic acid, and mobile phase B was acetonitrile containing 0.1 % formic acid. For Ursolic acid: Mobile phase A was water (10 mM Ammonium acetate), and mobile phase B was acetonitrile: methanol (3:1). Injections of 10 μL were performed using flow through a needle

(A) Eugenol:

Eugenol was quantified after derivatizing with pyridine sulfonyl chloride using estrone-d4 as an internal standard. Methanol was used to extract eugenol from fresh leaves (2 mg/mL) and dried stem powder (20 mg/ml). Briefly 10 μL of extract and 10 μL of internal standard (from 2.5 μg/mL) were added into 200 μL of buffer [acetone: NaHCO3 (1:1)]. To this 10 μL of pyridine sulfonyl chloride (10 mg/mL) was added and incubated at 60 °C for 15 min. After incubation the derivative was extracted with 800 μL of MTBE and the organic layer was dried and reconstituted in 50 μL of methanol followed by 10 μL injection for the analysis. A gradient (0–2 mins:30 %B, 2–5 mins:30–90 %B, 5–7 mins:90–100 %B, 7–10 mins:100 %B, 10–10.1 mins:100–30 %B, 10.1–15 mins:30) was then initiated at a flow rate of 200 μL/min. Operating conditions were as follows: spray voltage, 3000 V; ion transfer capillary temperature, 270 °C; source temperature 100 °C; sheath gas 20, auxiliary gas 5 (arbitrary units); collision gas, argon; S-lens voltage was optimized for individual metabolites; scan time of 50 millisec/transition; and ion polarity positive. A standard curve was constructed from 0.078 to 5ngon column to quantify eugenol. The SRM transition used for the analysis of eugenol is (306.1 → 79) and for estrone-d4 (416.3 → 274.1).

(B) Ursolic Acid:

Ursolic acid was quantified using estrone-d4 as an internal standard. A brief extraction was done from 2 mg/mL of dry powder using 1 mL of methanol (sonication-3 min, centrifugation −5 min). The extract was further diluted to 0.2 mg/mL in methanol. From this extract 10 μL was added along with 10 μL of internal standard (0.1 ug/mL) to 30 μL of methanol and 10 μL was injected for the analysis. A gradient (0–2 mins:20 %B, 2–8 mins:20–100 %B, 8–14.5 mins:100 %B, 14.5–14.6 mins:100–20 %B, 14.6–20 mins:20 %B) was then initiated at a flow rate of 200 μL/min. Operating conditions were as follows: Discharge current 4 μA; ion transfer capillary temperature, 270 °C; source temperature 300 °C; sheath gas 20, auxiliary gas 5 (arbitrary units); collision gas, argon; S-lens voltage was optimized for individual metabolites; scan time of 50 millisec/transition; and ion polarity positive. A standard curve was constructed from 0.034 to 2.5 ng on column to quantify ursolic acid. The same standard curve was used for the analysis of oleanolic acid. The SRM transition used for the analysis of both ursolic and oleanolic acid is (439.4 → 119) and for estrone-d4 (275.3 → 257.1).

### Availability of supporting data section

Information on the genes identified in Tulsi, along with the scaffold numbers, are provided in http://caps.ncbs.res.in/Ote.

BioProject : PRJNA251328

SRA id : SRP051184

Accession number of *O. tenuiflorum*: JQCZ00000000

Also please see DOI for supporting data: https://mynotebook.labarchives.com/share/National%2520Centre%2520for%2520Biological%2520Sciences/MTkuNXw2MjMwNC8xNS9UcmVlTm9kZS80MjAwNTk4MTM5fDQ5LjU=

Data available from the Dryad Digital Repository: http://dx.doi.org/10.5061/dryad.6f1r2

## Additional files

Additional file 1: Figure S1. Per base sequence quality of R1 reads of PE sequences used in final genome assembly. Additional file 2: Figure S2. Per base sequence quality of R2 reads of PE sequences used in final genome assembly. Additional file 3: Figure S3. Distribution of assembled scaffolds according to their length. Additional file 4: Figure S4. Distribution of scaffold length difference between paired end and paired with mate pair end assembly. Additional file 5: Table S1. Scaffold lengths distribution in the MP + PE and PE assemblies. Additional file 6: Table S2. Statistics of scaffold length comparison from assemblies of PE and MP + PE together. Additional file 7: Table S3. Completeness of assembly and presence of essential genes by CEGMA results for *O. tenuiflorum* at two levels; (a) only in PE assembly (b) in PE + PM assembly. Additional file 8: Table S4. Presence of essential genes in *O.tenuiflorum* (Tulsi) at three levels; a) in only paired end assembly (ab-initio gene prediction), b) in paired end and mate-pair assembly’s Level 2 [evidence from RNAseq, EST and known tulsi genes], c) in paired end and mate-paired assembly’s Level 1 (gene prediction). Additional file 9: Figure S5. Phylogenetic trees of essential gene, cytochrome P450 from *O.tenuiflorum* and their respective homologues. Additional file 10: Figure S6. NJ tree for glyceraldehydes phosphate dehydrogenase protein in *O. tenuiflorum* (Tulsi, marked in red) and its nearest homologues. Additional file 11: Figure S7. Phylogenetic trees of essential genes, actin from *O.tenuiflorum* and their respective homologues. Additional file 12: Table S5. Repeat elements identified in Tulsi genome assembly and classified in different groups of repeats. Additional file 13: Figure S8. Pie chart of distribution of protein domains (Pfam) of all the predicted genes in *O. tenuiflorum* subtype Krishna genome. Additional file 14: Table S6. List of species used for phylogeny analysis along with *Ocimum* to depict taxonomical distribution of this species in plant kingdom. Additional file 15: Figure S9. Circular representation of *O. tenuiflorum* metabolite-related genes mapped onto *Vitis vinefera* plant genome. Color indicate blue = < 2 genes, green =2 genes, yellowgreen = > 2 genes, red = Metabolite related genes. Connecting line between scaffolds and chromosome represents postion of the scaffold in genome. Red color of connecting line represents presence of metabolite related genes. Additional file 16: Figure S10. Circular representation of *O. tenuiflorum* metabolite-related genes mapped onto *Medicago tranculata* plant genome. Color indicate blue = < 2 genes, green =2 genes, yellowgreen = > 2 genes, red = Metabolite-related genes. Connecting line between scaffolds and chromosome represents postion of the scaffold in genome. Red color of connecting line represents presence of metabolite related genes. Additional file 17: Figure S11. Circular representation of *O. tenuiflorum* metabolite-related genes mapped onto *Arabidopsis thaliana* plant genome. Color indicate blue = < 2 genes, green =2 genes, yellowgreen = > 2 genes, red = Metabolite-related genes. Connecting line between scaffolds and chromosome represents postion of the scaffold in genome. Red color of connecting line represents presence of metabolite-related genes. Additional file 18: Table S7. Associations of *O. tenuiflorum* a) scaffolds related to metabolite-related genes, b) longer length scaffolds (greater than 10Kb in size) to four different plant genomes. Additional file 19: Table S8. All the transcripts with their expression and validation at different level such as genome hit and blast hit results against non-redundant database of NCBI. Additional file 20: Figure S12. Pathways of all the 14 important medicinal metabolites of the Tulsi genome which were studied in detail. Additional file 21: Table S9. Gene IDs involved in specialized metabolite production for each of the metabolites with known pathways. Additional file 22: Table S10. Sequences of putative terpene synthases in *O. tenuiflorum* genome. Additional file 23: Figure S13.
**a.** Sequence alignment of metabolite protein predicted from Scaffold 14352 from *Ocimum* and O65402 protein sequence from *Arabidopsis.* **b.** Sequence alignment of protein sequence predicted in scaffold16333 from *Ocimum* genome and Q8RWT0 protein sequence from *Arabidopsis.* **c.** Sequence alignment of protein sequence predicted in scaffold2032 from *Ocimum* genome and F1T282 protein sequence from *Vitis* proteome. Additional file 24:
**Text A.** List of scaffolds as marked from START to END in Fig. 4. Text B. List of IDs of transcripts more abundant in Krishna as compared to Rama subtype (from top to bottom) marked in Fig. 5a. Text C. List of IDs of transcripts more abundant in Rama as compared to Krishna subtype (from top to bottom) as marked in Fig. 5b. Text D. Multiple sequence alignment of amyrin synthases with hits in Tulsi genome. Additional file 25: Table S11. Metabolites with unknown pathways with their disease implications. There are 15 medicinally relevant metabolites in *Ocimum sp.* with unknown pathways.

## Abbreviations

PE: Paired end
MP: Mate paired
CEGMA: Core eukaryotic genes mapping approach
DEG: Database of essential genes
LTR: Long terminal repeats
Ote: *Ocimum tenuiflorum*
Ath: *Arabidopsis thaliana*
Mgu: *Mimulus guttatus*
Sly: *Solanum lycopersicum*
Osa: *Oryza sativa* (Osa)
SSR: Simple sequence repeats
CHS: chalcone synthase
RbcS: Rubisco small subunit
MSA: Multiple sequence alignment
NJ: Neighbor joining

## References

[1] Paton A, Harley RM, and Harley MM. Ocimum-an overview of relationships and classification. Ocimum Aromatic Plants-Industrial Profiles. Amsterdam: Harwood Academic (1999)

[2] Willis JC (1919). A dictionary of the flowering plants and ferns, by J. C. Willis.

[3] Prakash P, Gupta N (2005). Therapeutic uses of Ocimum sanctum Linn (Tulsi) with a note on eugenol and its pharmacological actions: a short review. Indian J Physiol Pharmacol.

[4] Khare CP. Indian medicinal plants: an illustrated dictionary. Springer Science & Business Media; 2007.

[5] Rao PS, Satelli A, Moridani M, Jenkins M, Rao US (2012). Luteolin induces apoptosis in multidrug resistant cancer cells without affecting the drug transporter function: involvement of cell line-specific apoptotic mechanisms. Int J Cancer.

[6] Góngora-castillo E, Fedewa G, Yeo Y, Chappell J, Dellapenna D, Buell CR (2012). Genomic approaches for interrogating the biochemistry of medicinal plant species. Methods Enzymol.

[7] Rastogi S, Meena S, Bhattacharya A, Ghosh S, Shukla RK, Sangwan NS (2014). De novo sequencing and comparative analysis of holy and sweet basil transcriptomes. BMC Genomics.

[8] Carović-Stanko K, Liber Z, Besendorfer V, Javornik B, Bohanec B, Kolak I (2009). Genetic relations among basil taxa (Ocimum L.) based on molecular markers, nuclear DNA content, and chromosome number. Plant Syst Evol.

[9] MORTON JK (1962). Cytotaxonomic studies on the West African Labiatae. J Linn Soc London, Bot.

[10] Khosla M, Sobti SN. Karyomorphological Studies in Genus Ocimum II. Sanctum Group. Cytologia 50: 253-263, 1935.

[11] Parra G, Bradnam K, Korf I (2007). CEGMA: a pipeline to accurately annotate core genes in eukaryotic genomes. Bioinformatics.

[12] Zhang R, Ou H-Y, Zhang C-T (2004). DEG: a database of essential genes. Nucleic Acids Res.

[13] Apweiler R, Bairoch A, Wu CH, Barker WC, Boeckmann B, Ferro S (2004). UniProt: the Universal Protein knowledgebase. Nucleic Acids Res.

[14] Ferrer JL, Jez JM, Bowman ME, Dixon RA, Noel JP (1999). Structure of chalcone synthase and the molecular basis of plant polyketide biosynthesis. Nat Struct Biol.

[15] Portnoy V, Benyamini Y, Bar E, Harel-Beja R, Gepstein S, Giovannoni JJ (2008). The molecular and biochemical basis for varietal variation in sesquiterpene content in melon (Cucumis melo L.) rinds. Plant Mol Biol.

[16] Plant Metabolic Network (PMN), http://www.plantcyc.org/tools/tools_overview.faces on www.plantcyc.org, February 28, 2008. [http://www.plantcyc.org/about/citing_pmn.faces]. Date Accessed 05-08-2014.

[17] Brendolise C, Yauk Y-K, Eberhard ED, Wang M, Chagne D, Andre C (2011). An unusual plant triterpene synthase with predominant α-amyrin-producing activity identified by characterizing oxidosqualene cyclases from Malus × domestica. FEBS J.

[18] Poralla K, Hewelt A, Prestwich GD, Abe I, Reipen I, Sprenger G (1994). A specific amino acid repeat in squalene and oxidosqualene cyclases. Trends Biochem Sci.

[19] Abe I, Prestwich GD (1995). Identification of the active site of vertebrate oxidosqualene cyclase. Lipids.

[20] Kushiro T, Shibuya M, Masuda K, Ebizuka Y. Mutational studies on triterpene synthases: engineering lupeol synthase into -amyrin synthase. 2000:6816–6824.

[21] Bast F, Rani P, Meena D (2014). Chloroplast DNA phylogeography of holy basil (Ocimum tenuiflorum) in Indian subcontinent. ScientificWorldJournal.

[22] Wang W, Feng B, Xiao J, Xia Z, Zhou X, Li P (2014). Cassava genome from a wild ancestor to cultivated varieties. Nat Commun.

[23] Ghosh P, Chattopadhyay SK, Adhikari S, Saha S, Mondal S. A high throughput DNA extraction method from chemotypically heterogeneous plant species. P. 2013;2603.

[24] Lab H. FASTX toolkit. http://hannonlab.cshl.edu/fastx_toolkit/index.html.

[25] Andrews S. FastQC a quality control tool for high throughput sequence data. http://www.bioinformatics.babraham.ac.uk/projects/fastqc/.

[26] Xie Y, Wu G, Tang J, Luo R, Patterson J, Liu S (2014). SOAPdenovo-Trans: de novo transcriptome assembly with short RNA-Seq reads. Bioinformatics.

[27] Cantarel BL, Korf I, Robb SMC, Parra G, Ross E, Moore B (2008). MAKER: an easy-to-use annotation pipeline designed for emerging model organism genomes. Genome Res.

[28] Stanke M, Morgenstern B (2005). AUGUSTUS: a web server for gene prediction in eukaryotes that allows user-defined constraints. Nucleic Acids Res.

[29] Hunt M, Kikuchi T, Sanders M, Newbold C, Berriman M, Otto TD (2013). REAPR: a universal tool for genome assembly evaluation. Genome Biol.

[30] Heng L, Bob H, Alec W, Tim F, Jue Ruan NH, Gabor M (2009). The Sequence Alignment/Map format and SAMtools.pdf. Bioinformatics.

[31] Marçais G, Kingsford C (2011). A fast, lock-free approach for efficient parallel counting of occurrences of k-mers. Bioinformatics.

[32] Tatusov RL, Fedorova ND, Jackson JD, Jacobs AR, Kiryutin B, Koonin EV (2003). The COG database: an updated version includes eukaryotes. BMC Bioinformatics.

[33] Altschul SF, Madden TL, Schaffer AA, Zhang J, Zhang Z, Miller W (1997). Gapped BLAST and PSI-BLAST: a new generation of protein database search programs. Nucleic Acids Res.

[34] Price AL, Jones NC, Pevzner PA (2005). De novo identification of repeat families in large genomes. Bioinformatics.

[35] RepeatMasker open-3.0.;1996–2010. http://www.repeatmasker.org. [http://www.repeatmasker.org/faq.html#faq3]

[36] Slater GSC, Birney E (2005). Automated generation of heuristics for biological sequence comparison. BMC Bioinformatics.

[37] Mistry J, Finn RD, Eddy SR, Bateman A, Punta M (2013). Challenges in homology search: HMMER3 and convergent evolution of coiled-coil regions. Nucleic Acids Res.

[38] Li L, Stoeckert CJ, Roos DS (2003). OrthoMCL: identification of ortholog groups for eukaryotic genomes. Genome Res.

[39] Lechner M, Findeiss S, Steiner L, Marz M, Stadler PF, Prohaska SJ (2011). Proteinortho: detection of (co-) orthologs in large-scale analysis. BMC Bioinformatics.

[40] Goodstein DM, Shu S, Howson R, Neupane R, Hayes RD, Fazo J (2012). Phytozome: a comparative platform for green plant genomics. Nucleic Acids Res.

[41] Larkin MA, Blackshields G, Brown NP, Chenna R, McGettigan PA, McWilliam H (2007). Clustal W and Clustal X version 2.0. Bioinformatics.

[42] Felsenstein J (1989). PHYLIP - phylogeny inference package (version 3.2). Cladistics.

[43] Kent WJ (2002). BLAT---the BLAST-like alignment tool. Genome Res.

[44] Kim D, Pertea G, Trapnell C, Pimentel H, Kelley R, Salzberg SL (2013). TopHat2: accurate alignment of transcriptomes in the presence of insertions, deletions and gene fusions. Genome Biol.

[45] Langmead B, Salzberg SL (2012). Fast gapped-read alignment with Bowtie 2. Nat Methods.

[46] Li W, Godzik A (2006). Cd-hit: a fast program for clustering and comparing large sets of protein or nucleotide sequences. Bioinformatics.

[47] Góngora-Castillo E, Buell CR (2013). Bioinformatics challenges in de novo transcriptome assembly using short read sequences in the absence of a reference genome sequence. Nat Prod Rep.

[48] Jiang H, Wong WH (2008). SeqMap: mapping massive amount of oligonucleotides to the genome. Bioinformatics.

[49] Salzman J, Jiang H, Wong WH (2011). Statistical modeling of RNA-seq data. Stat Sci.

