# Supplementary figures and images for: Genome sequencing of herb Tulsi (Ocimum tenuiflorum) unravels key genes behind its strong medicinal properties
Source: BMC Plant Biol. 2015 Aug 28;15:212. doi: 10.1186/s12870-015-0562-x (PMC4552454; doi:10.1186/s12870-015-0562-x)

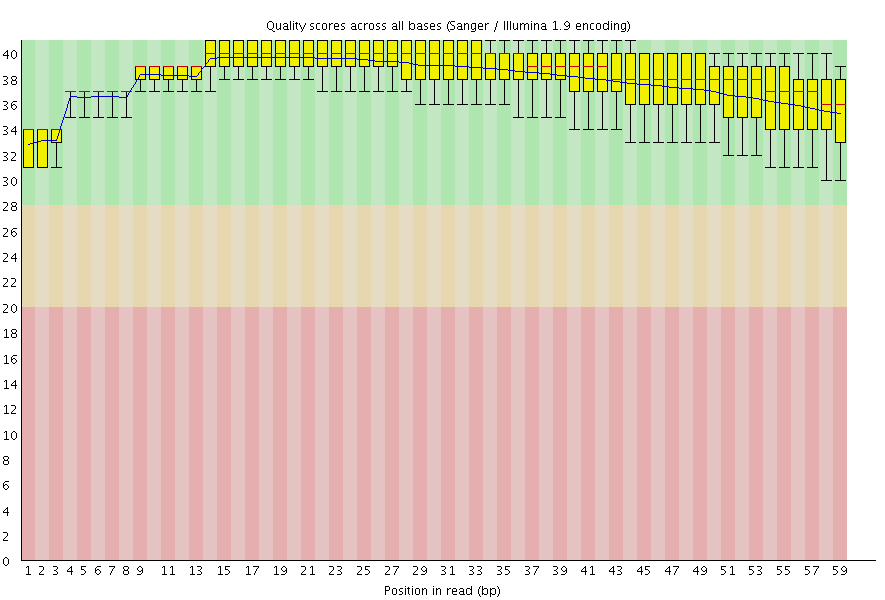

Supplement: Additional file 1: Figure S1. — Per base sequence quality of R1 reads of PE sequences used in final genome assembly. [file 12870_2015_562_MOESM1_ESM.png]

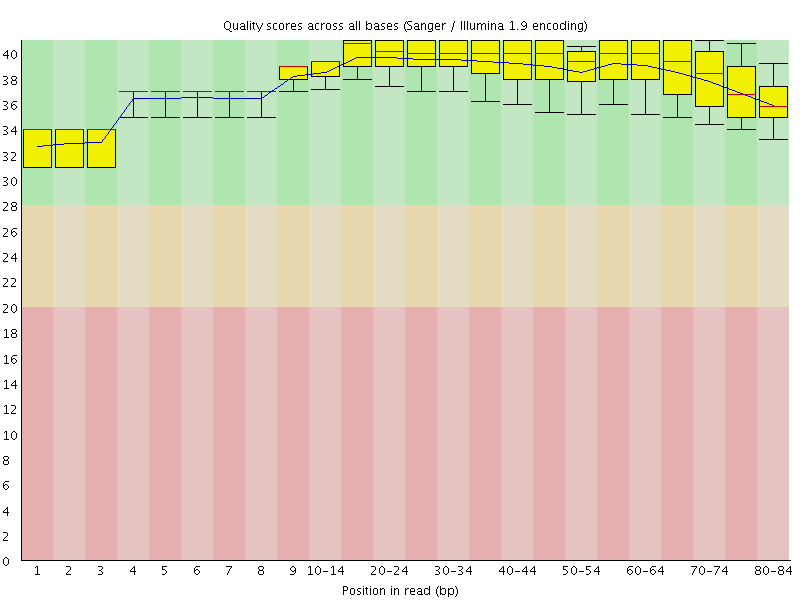

Supplement: Additional file 2: Figure S2. — Per base sequence quality of R2 reads of PE sequences used in final genome assembly. [file 12870_2015_562_MOESM2_ESM.png]

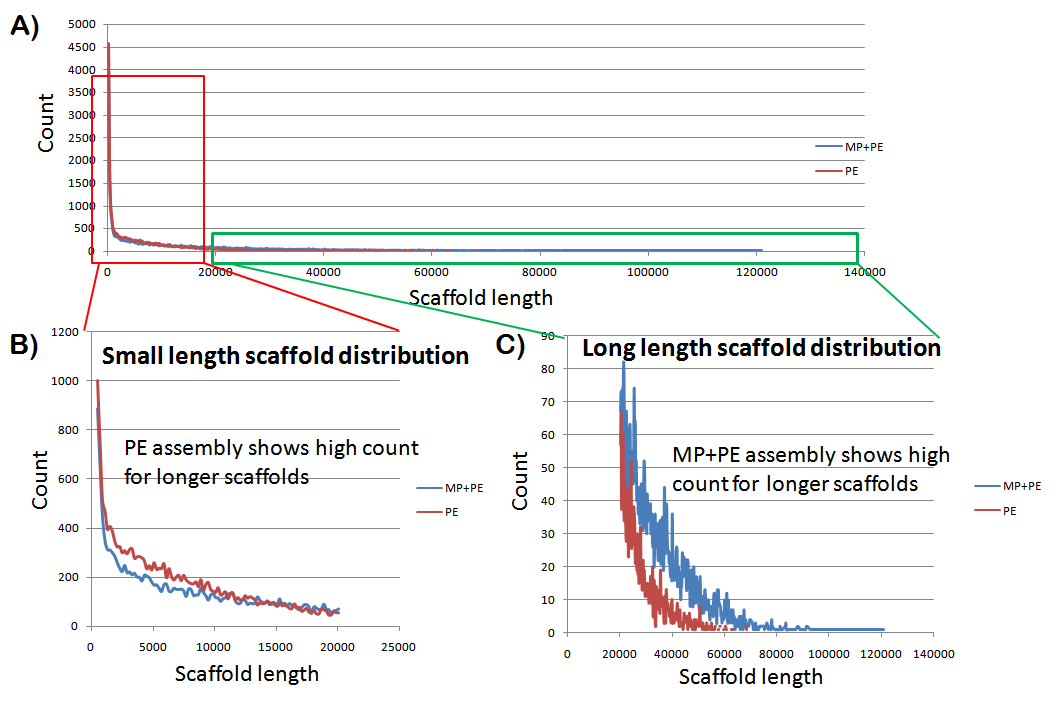

Supplement: Additional file 3: Figure S3. — Distribution of assembled scaffolds according to their length. [file 12870_2015_562_MOESM3_ESM.png]

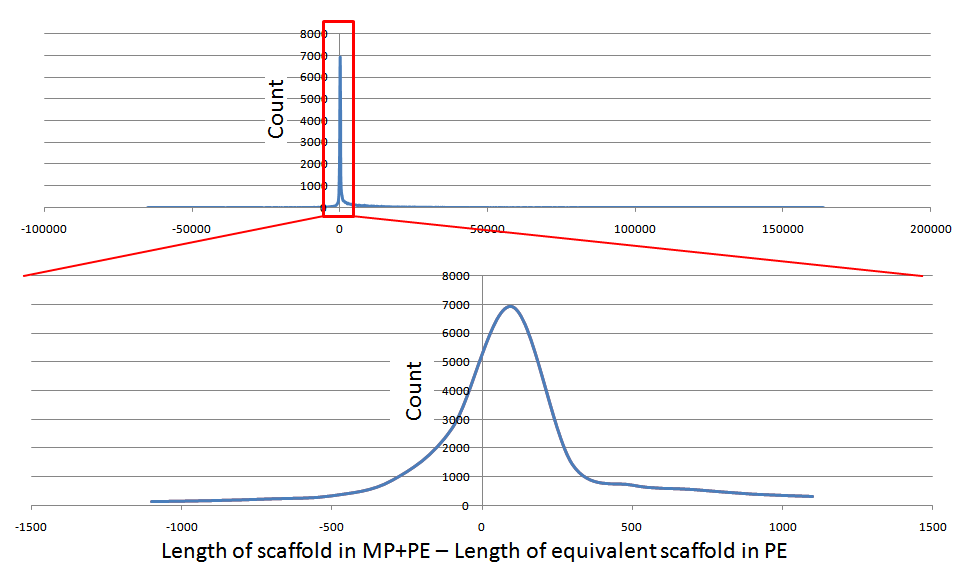

Supplement: Additional file 4: Figure S4. — Distribution of scaffold length difference between paired end and paired with mate pair end assembly. [file 12870_2015_562_MOESM4_ESM.png]

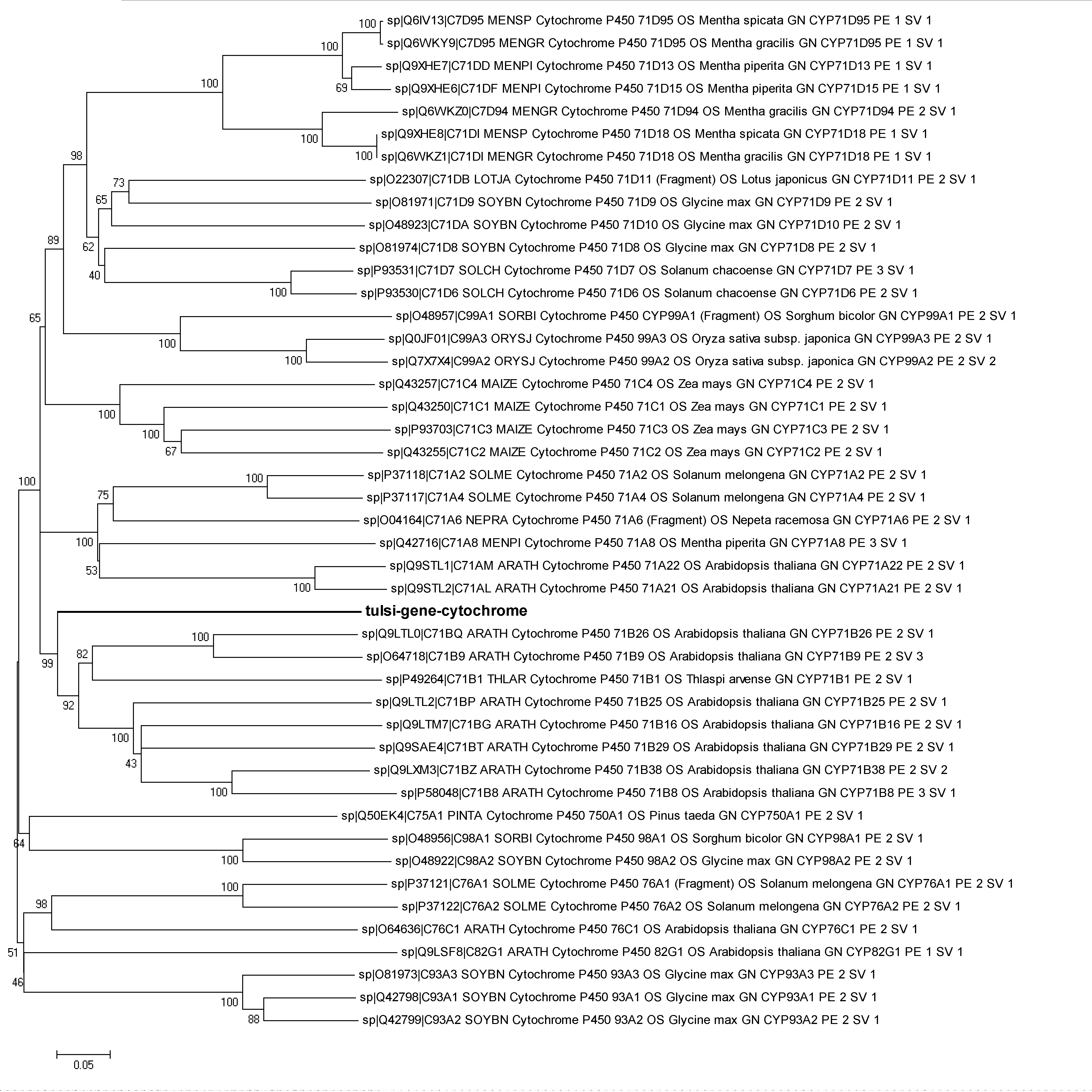

Supplement: Additional file 9: Figure S5. — Phylogenetic trees of essential gene, cytochrome P450 from O.tenuiflorum and their respective homologues. [file 12870_2015_562_MOESM9_ESM.png]

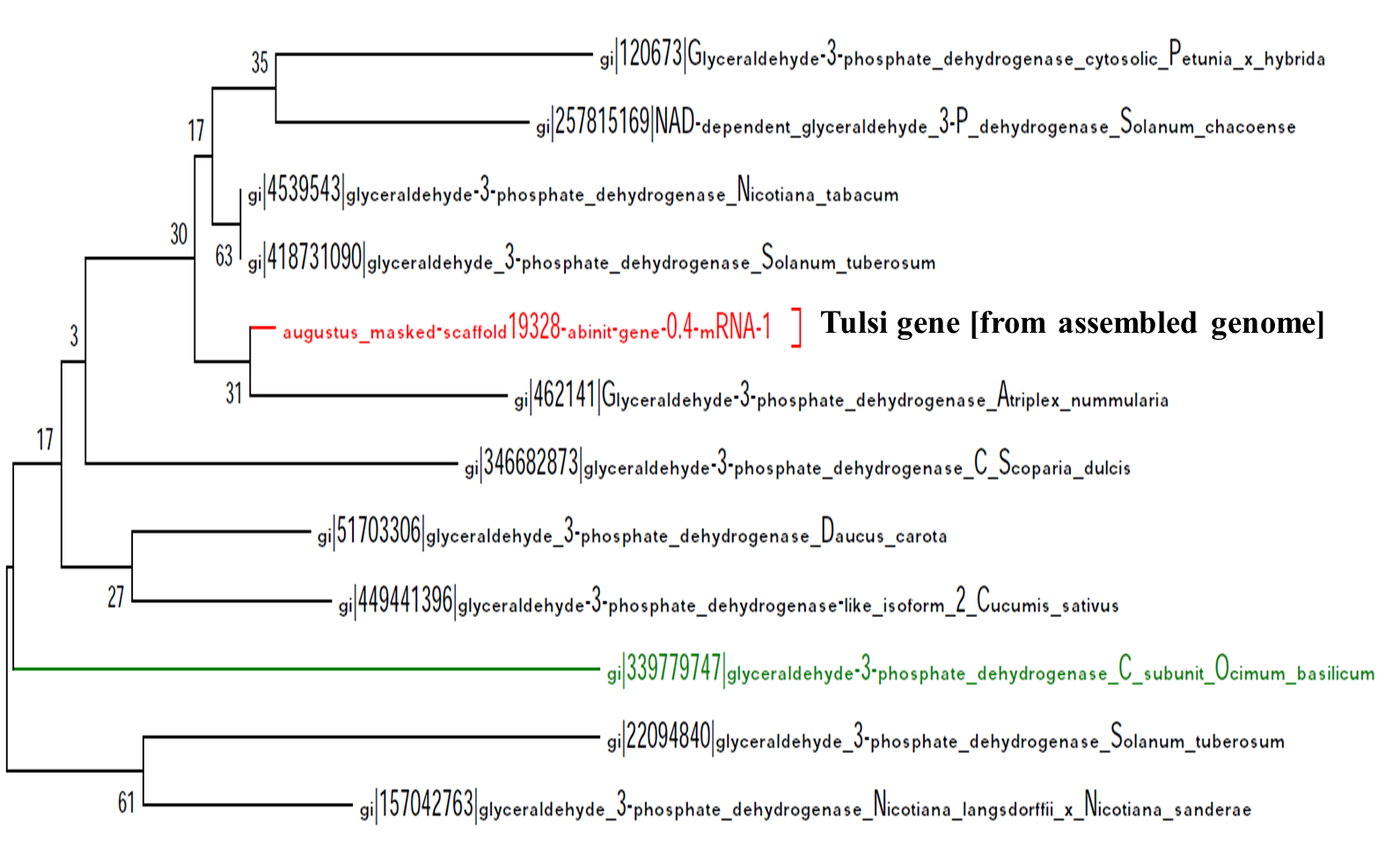

Supplement: Additional file 10: Figure S6. — NJ tree for glyceraldehydes phosphate dehydrogenase protein in O. tenuiflorum (Tulsi, marked in red) and its nearest homologues. [file 12870_2015_562_MOESM10_ESM.png]

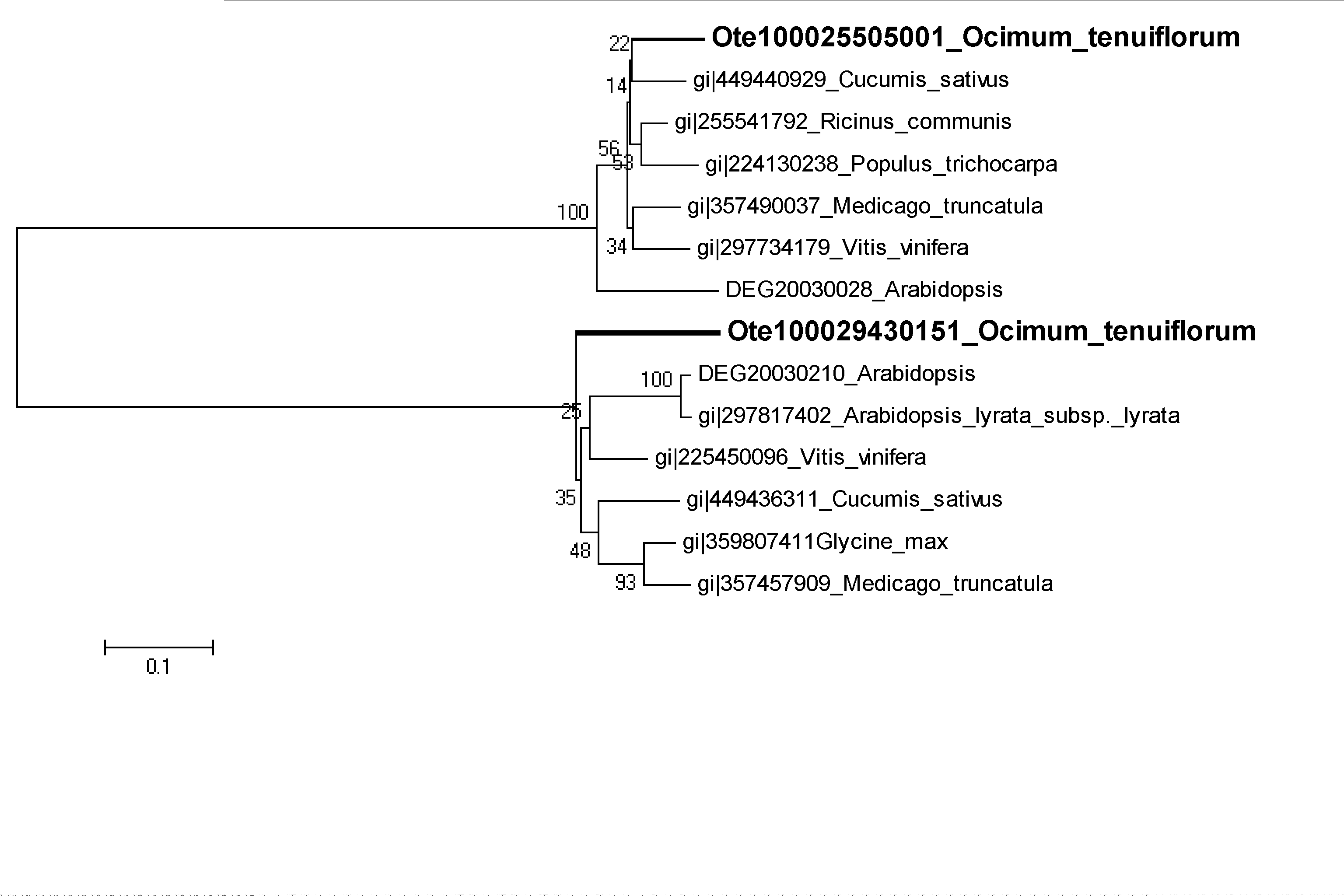

Supplement: Additional file 11: Figure S7. — Phylogenetic trees of essential genes, actin from O.tenuiflorum and their respective homologues. [file 12870_2015_562_MOESM11_ESM.png]

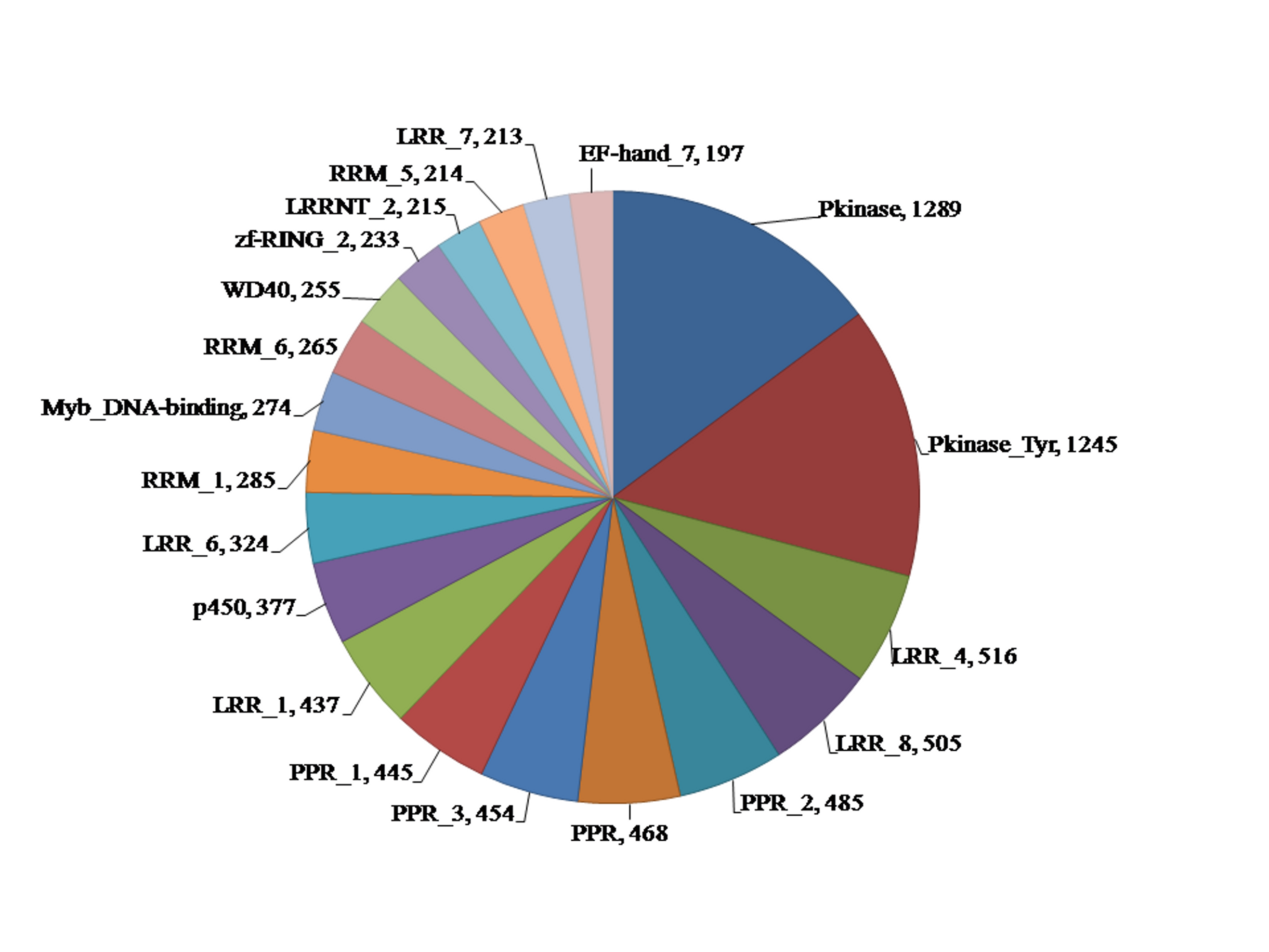

Supplement: Additional file 13: Figure S8. — Pie chart of distribution of protein domains (Pfam) of all the predicted genes in O. tenuiflorum subtype Krishna genome. [file 12870_2015_562_MOESM13_ESM.tif]

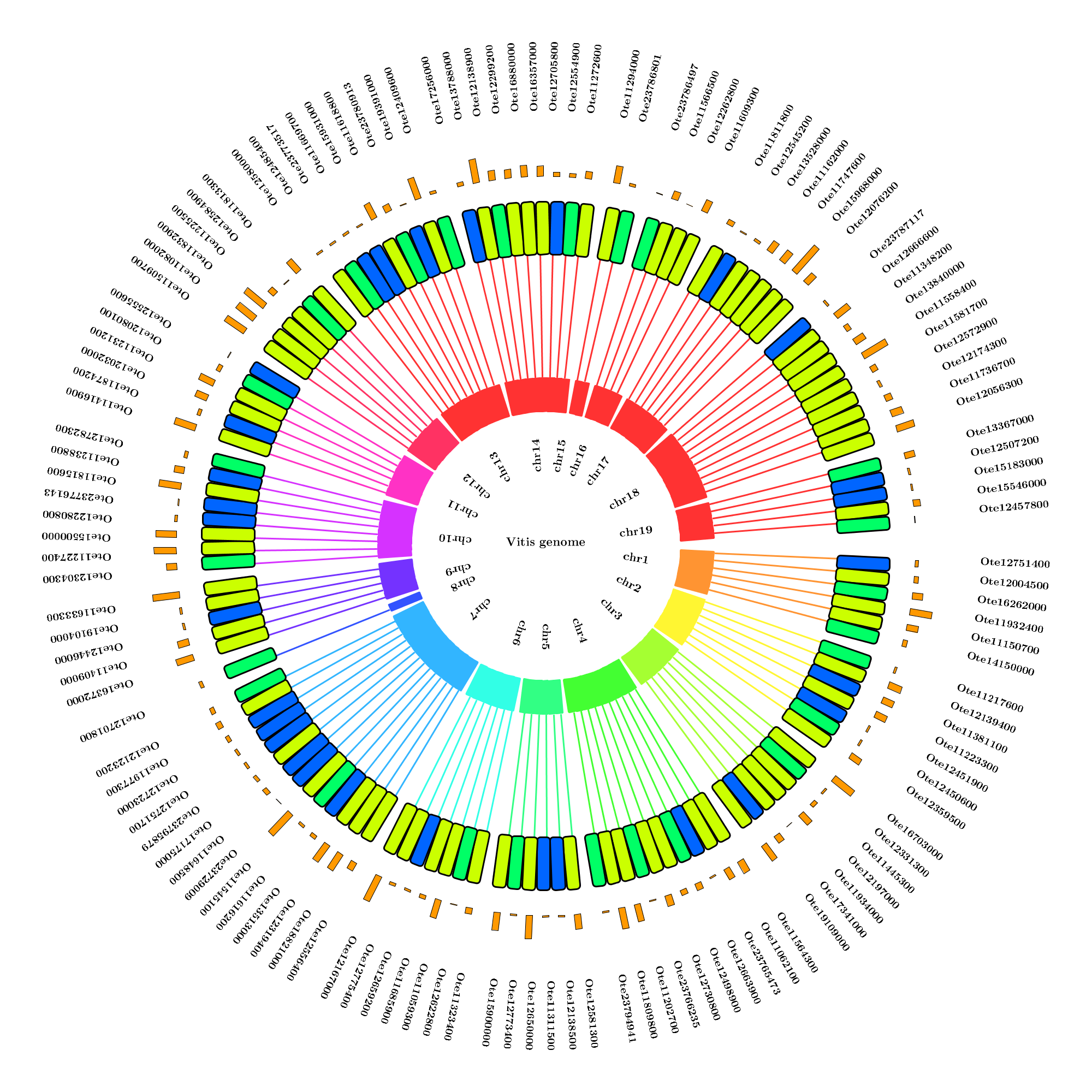

Supplement: Additional file 15: Figure S9. — Circular representation of O. tenuiflorum metabolite-related genes mapped onto Vitis vinefera plant genome. Color indicate blue = < 2 genes, green =2 genes, yellowgreen = > 2 genes, red = Metabolite related genes. Connecting line between scaffolds and chromosome represents postion of the scaffold in genome. Red color of connecting line represents presence of metabolite related genes. [file 12870_2015_562_MOESM15_ESM.png]

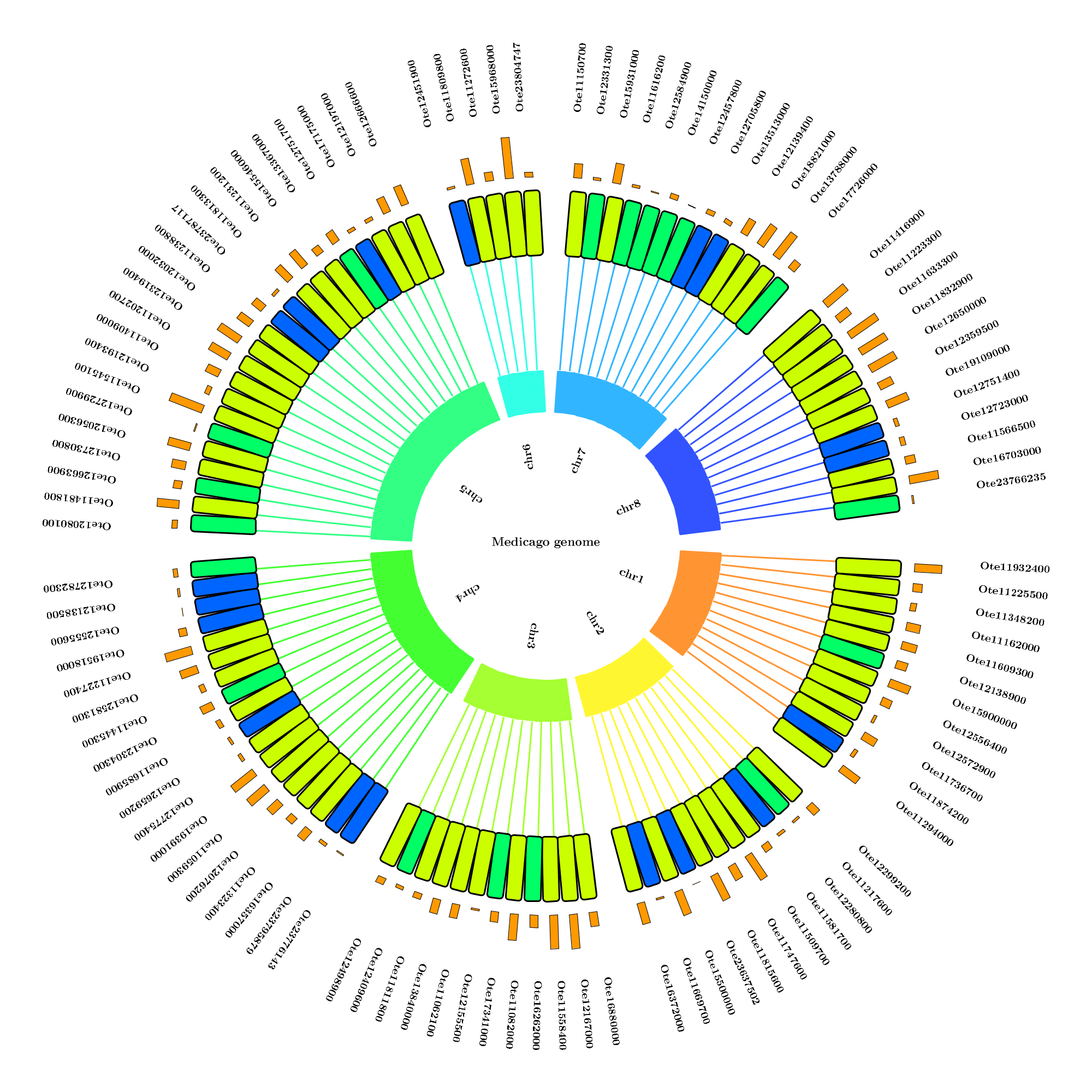

Supplement: Additional file 16: Figure S10. — Circular representation of O. tenuiflorum metabolite-related genes mapped onto Medicago tranculata plant genome. Color indicate blue = < 2 genes, green =2 genes, yellowgreen = > 2 genes, red = Metabolite-related genes. Connecting line between scaffolds and chromosome represents postion of the scaffold in genome. Red color of connecting line represents presence of metabolite related genes. [file 12870_2015_562_MOESM16_ESM.png]

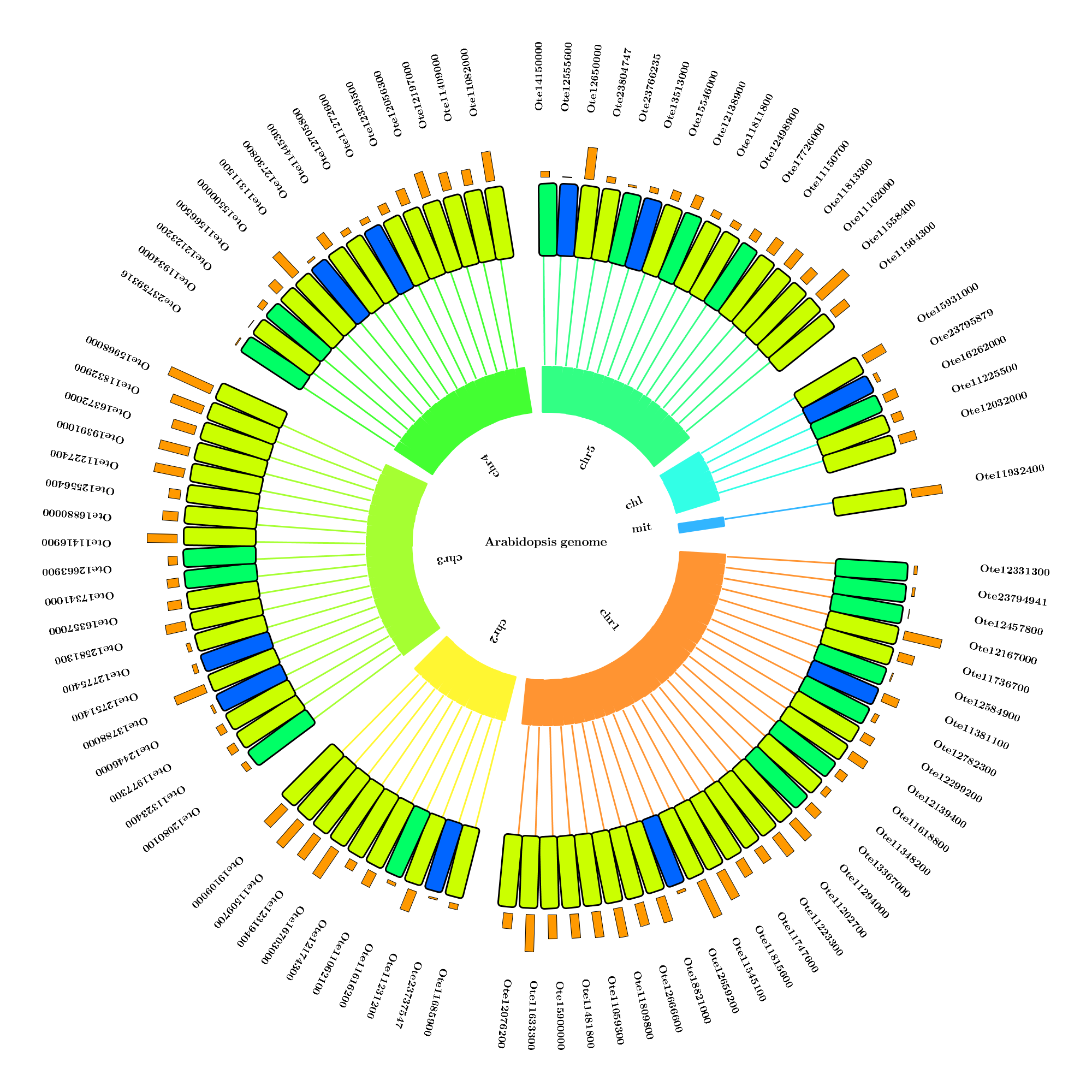

Supplement: Additional file 17: Figure S11. — Circular representation of O. tenuiflorum metabolite-related genes mapped onto Arabidopsis thaliana plant genome. Color indicate blue = < 2 genes, green =2 genes, yellowgreen = > 2 genes, red = Metabolite-related genes. Connecting line between scaffolds and chromosome represents postion of the scaffold in genome. Red color of connecting line represents presence of metabolite-related genes. [file 12870_2015_562_MOESM17_ESM.png]

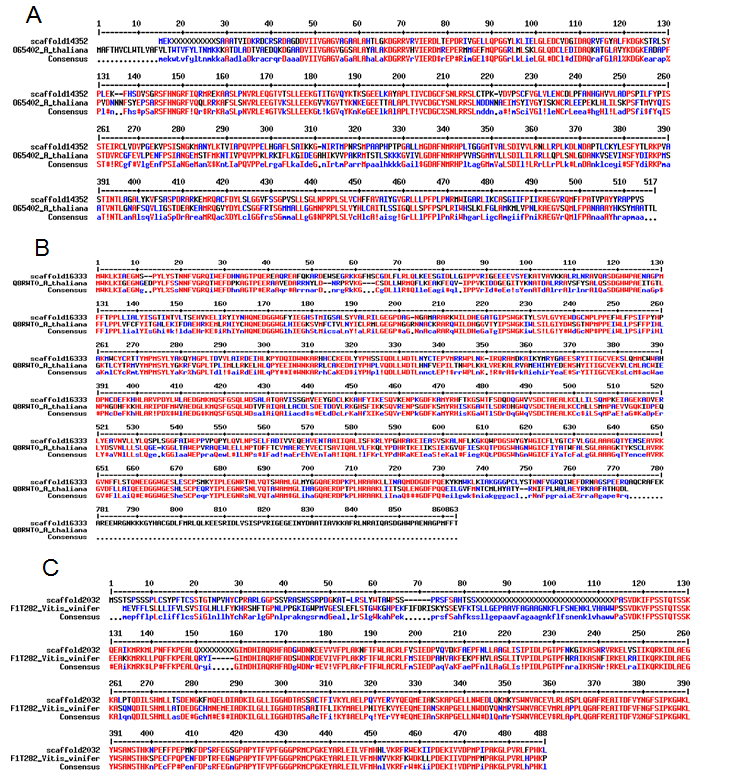

Supplement: Additional file 23: Figure S13. — a. Sequence alignment of metabolite protein predicted from Scaffold 14352 from Ocimum and O65402 protein sequence from Arabidopsis. b. Sequence alignment of protein sequence predicted in scaffold16333 from Ocimum genome and Q8RWT0 protein sequence from Arabidopsis. c. Sequence alignment of protein sequence predicted in scaffold2032 from Ocimum genome and F1T282 protein sequence from Vitis proteome. [file 12870_2015_562_MOESM23_ESM.png]
