# Supplementary material for: Genome sequencing of herb Tulsi (Ocimum tenuiflorum) unravels key genes behind its strong medicinal properties
Source: BMC Plant Biol. 2015 Aug 28;15:212. doi: 10.1186/s12870-015-0562-x (PMC4552454; doi:10.1186/s12870-015-0562-x)
Supplement: Additional file 5: Table S1. — Scaffold lengths distribution in the MP + PE and PE assemblies. [file 12870_2015_562_MOESM5_ESM.doc]

| **Category** | **MP+PE** | **PE** |
| --- | --- | --- |
| Length of longest scaffold | 1,84,679 | 1,05,702 |
| Number of scaffolds | 78,224 | 1,31,419 |

Supplementary Table 1: Scaffold lengths in the MP+PE and PE assemblies
