# Supplementary material for: Genome sequencing of herb Tulsi (Ocimum tenuiflorum) unravels key genes behind its strong medicinal properties
Source: BMC Plant Biol. 2015 Aug 28;15:212. doi: 10.1186/s12870-015-0562-x (PMC4552454; doi:10.1186/s12870-015-0562-x)
Supplement: Additional file 6: Table S2. — Statistics of scaffold length comparison from assemblies of PE and MP + PE together. [file 12870_2015_562_MOESM6_ESM.doc]

| **Category** | **Number of scaffolds** |
| --- | --- |
| Scaffolds in MP+PE are longer than PE assembly | 21,187 |
| Length of scaffolds in MP+PE are equal to PE assembly | 95 |
| Scaffolds in PE are longer than MP+PE assembly | 6,117 |
| **Total** | **27,399** |

Supplementary Table 2: Statistics of scaffold length comparison
