# Supplementary material for: Genome sequencing of herb Tulsi (Ocimum tenuiflorum) unravels key genes behind its strong medicinal properties
Source: BMC Plant Biol. 2015 Aug 28;15:212. doi: 10.1186/s12870-015-0562-x (PMC4552454; doi:10.1186/s12870-015-0562-x)
Supplement: Additional file 7: Table S3. — Completeness of assembly and presence of essential genes by CEGMA results for O. tenuiflorum at two levels; (a) only in PE assembly (b) in PE + PM assembly. [file 12870_2015_562_MOESM7_ESM.doc]

| Features | PE data | MP + PE data |
| --- | --- | --- |
| Number of complete proteins detected | 205 | 211 |
| Percent of complete proteins detected | 82.6% | 85.08% |
| Number of partial proteins detected | 237 | 236 |
| Percent of partial proteins detected | 95.56% | 95.16% |

Supplementary Table 3: Completeness of assembly and presence of essential genes by CEGMA results for *O. tenuiflorum* at two levels; (a) only in PE assembly (b) in PE + PM assembly
