# Supplementary material for: Genome sequencing of herb Tulsi (Ocimum tenuiflorum) unravels key genes behind its strong medicinal properties
Source: BMC Plant Biol. 2015 Aug 28;15:212. doi: 10.1186/s12870-015-0562-x (PMC4552454; doi:10.1186/s12870-015-0562-x)
Supplement: Additional file 8: Table S4. — Presence of essential genes in O.tenuiflorum (Tulsi) at three levels; a) in only paired end assembly (ab-initio gene prediction), b) in paired end and mate-pair assembly’s Level 2 [evidence from RNAseq, EST and known tulsi genes], c) in paired end and mate-paired assembly’s Level 1 (gene prediction). [file 12870_2015_562_MOESM8_ESM.doc]

|  | **a) Paired End** | **b) Paired End + Mate Paired** | **c) Paired End + Mate Paired** |
| --- | --- | --- | --- |
| **Status** | Level1, unmasked [Arabidopsis model] | Level 2,  42% repeat masked | Level1,   42% repeat masked |
| **No. of genes** | 28,374 | 22,110 | 41,663 |
| **Matches with DEG [total 356 DEG genes]** | 341 | 335 | 353 |
| **PFAM validation  (>75% Query coverage)** | 86.0% | 89.0% | 86.5% |
| **PFAM validation (<75% Query coverage)** | 14.0% | 11.0% | 13.5% |

Supplementary Table 4: Presence of essential genes in *O.tenuiflorum* (Tulsi) at three levels; a) in only paired end assembly (ab-initio gene prediction), b) in paired end and mate-pair assembly’s Level 2 [evidence from RNAseq, EST and known tulsi genes], c) in paired end and mate-paired assembly’s Level 1 ( gene prediction).
