# Supplementary material for: Genome sequencing of herb Tulsi (Ocimum tenuiflorum) unravels key genes behind its strong medicinal properties
Source: BMC Plant Biol. 2015 Aug 28;15:212. doi: 10.1186/s12870-015-0562-x (PMC4552454; doi:10.1186/s12870-015-0562-x)
Supplement: Additional file 12: Table S5. — Repeat elements identified in Tulsi genome assembly and classified in different groups of repeats. [file 12870_2015_562_MOESM12_ESM.doc]

| **Type of repeat elements** | **number of elements** | **length occupied (in base pairs)** | **Percentage of sequence (%)** |
| --- | --- | --- | --- |
| **SINEs:** | 0 | 0 | 0 |
| **ALUs** | 0 | 0 | 0 |
| **MIRs** | 0 | 0 | 0 |
| **LINEs:** | 4929 | 1137713 | 0.3 |
| **LINE1** | 3746 | 1061531 | 0.28 |
| **LINE2** | 523 | 26773 | 0.01 |
| **L3/CR1** | 118 | 6398 | 0 |
| **LTR elements:** | 71642 | 41485830 | 11.07 |
| **ERVL** | 27 | 981 | 0 |
| **ERVL-MaLRs** | 0 | 0 | 0 |
| **ERV_classI** | 221 | 10571 | 0 |
| **ERV_classII** | 72 | 3849 | 0 |
| **DNA elements:** | 16982 | 5079052 | 1.36 |
| **hAT-Charlie** | 73 | 4132 | 0 |
| **TcMar-Tigger** | 14 | 819 | 0 |
| **Unclassified:** | 443518 | 104898239 | 27.99 |
| **Total interspersed repeats** |  | 152600834 | 40.71 |
| **Small RNA** | 0 | 0 | 0 |
| **Satellites:** | 0 | 0 | 0 |
| **Simple repeats** | 109946 | 8673640 | 2.31 |
| **Low complexity** | 17569 | 868822 | 0.23 |

Supplementary Table 5: Repeat elements identified in tulsi genome assembly
