# Supplementary material for: Genome sequencing of herb Tulsi (Ocimum tenuiflorum) unravels key genes behind its strong medicinal properties
Source: BMC Plant Biol. 2015 Aug 28;15:212. doi: 10.1186/s12870-015-0562-x (PMC4552454; doi:10.1186/s12870-015-0562-x)
Supplement: Additional file 20: Figure S12. — Pathways of all the 14 important medicinal metabolites of the Tulsi genome which were studied in detail. [file 12870_2015_562_MOESM20_ESM.pdf]

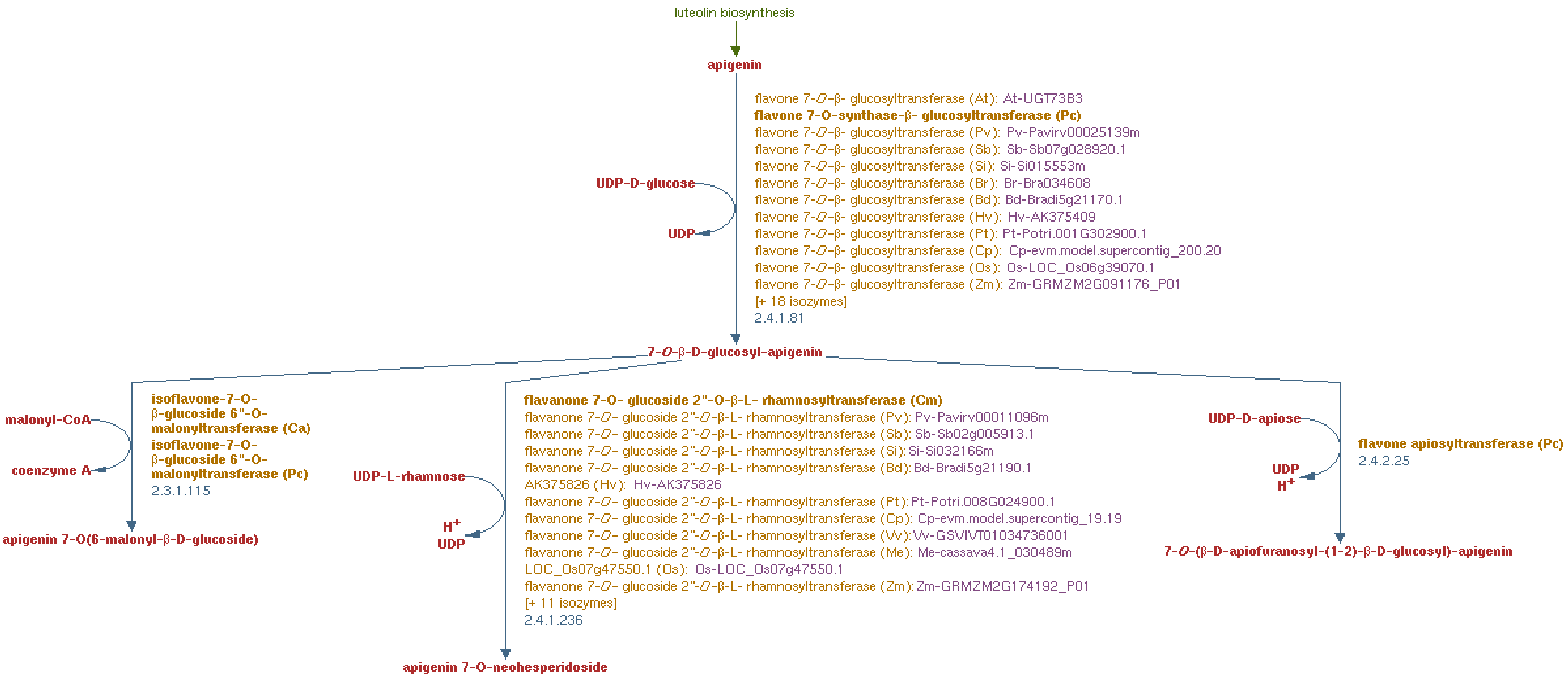
luteolin biosynthesis
apigenin
flavone 7-O-β- glucosyltransferase (At): At-UGT73B3
flavone 7-O-synthase-β- glucosyltransferase (Pc)
flavone 7-O-β- glucosyltransferase (Pv): Pv-Pavirv00025139m
flavone 7-O-β- glucosyltransferase (Sb): Sb-Sb07g028920.1
flavone 7-O-β- glucosyltransferase (Si): Si-Si015553m
flavone 7-O-β- glucosyltransferase (Br): Br-Bra034608
flavone 7-O-β- glucosyltransferase (Bd): Bd-Bradi5g21170.1
flavone 7-O-β- glucosyltransferase (Hv): Hv-AK375409
flavone 7-O-β- glucosyltransferase (Pt): Pt-Potri.001G302900.1
flavone 7-O-β- glucosyltransferase (Cp): Cp-evm.model.supercontig_200.20
flavone 7-O-β- glucosyltransferase (Os): Os-LOC_Os06g39070.1
flavone 7-O-β- glucosyltransferase (Zm): Zm-GRMZM2G091176_P01
[+ 18 isozymes]
2.4.1.81
UDP-D-glucose
UDP
7-O-β-D-glucosyl-apigenin
malonyl-CoA
coenzyme A
isoflavone-7-O-β-glucoside 6"-O-malonyltransferase (Ca)
isoflavone-7-O-β-glucoside 6"-O-malonyltransferase (Pc)
2.3.1.115
apigenin 7-O(6-malonyl-β-D-glucoside)
UDP-L-rhamnose
H+
UDP
flavanone 7-O- glucoside 2"-O-β-L- rhamnosyltransferase (Cm)
flavanone 7-O- glucoside 2"-O-β-L- rhamnosyltransferase (Pv): Pv-Pavirv00011096m
flavanone 7-O- glucoside 2"-O-β-L- rhamnosyltransferase (Sb): Sb-Sb02g005913.1
flavanone 7-O- glucoside 2"-O-β-L- rhamnosyltransferase (Si): Si-Si032166m
flavanone 7-O- glucoside 2"-O-β-L- rhamnosyltransferase (Bd): Bd-Bradi5g21190.1
AK375826 (Hv): Hv-AK375826
flavanone 7-O- glucoside 2"-O-β-L- rhamnosyltransferase (Pt): Pt-Potri.008G024900.1
flavanone 7-O- glucoside 2"-O-β-L- rhamnosyltransferase (Cp): Cp-evm.model.supercontig_19.19
flavanone 7-O- glucoside 2"-O-β-L- rhamnosyltransferase (Vv): Vv-GSVIVT01034736001
flavanone 7-O- glucoside 2"-O-β-L- rhamnosyltransferase (Me): Me-cassava4.1_030489m
LOC_Os07g47550.1 (Os): Os-LOC_Os07g47550.1
flavanone 7-O- glucoside 2"-O-β-L- rhamnosyltransferase (Zm): Zm-GRMZM2G174192_P01
[+ 11 isozymes]
2.4.1.236
apigenin 7-O-neohesperidoside
UDP-D-apiose
UDP
H+
flavone apiosyltransferase (Pc)
2.4.2.25
7-O-(β-D-apiofuranosyl-(1-2)-β-D-glucosyl)-apigenin

1. Apigenin

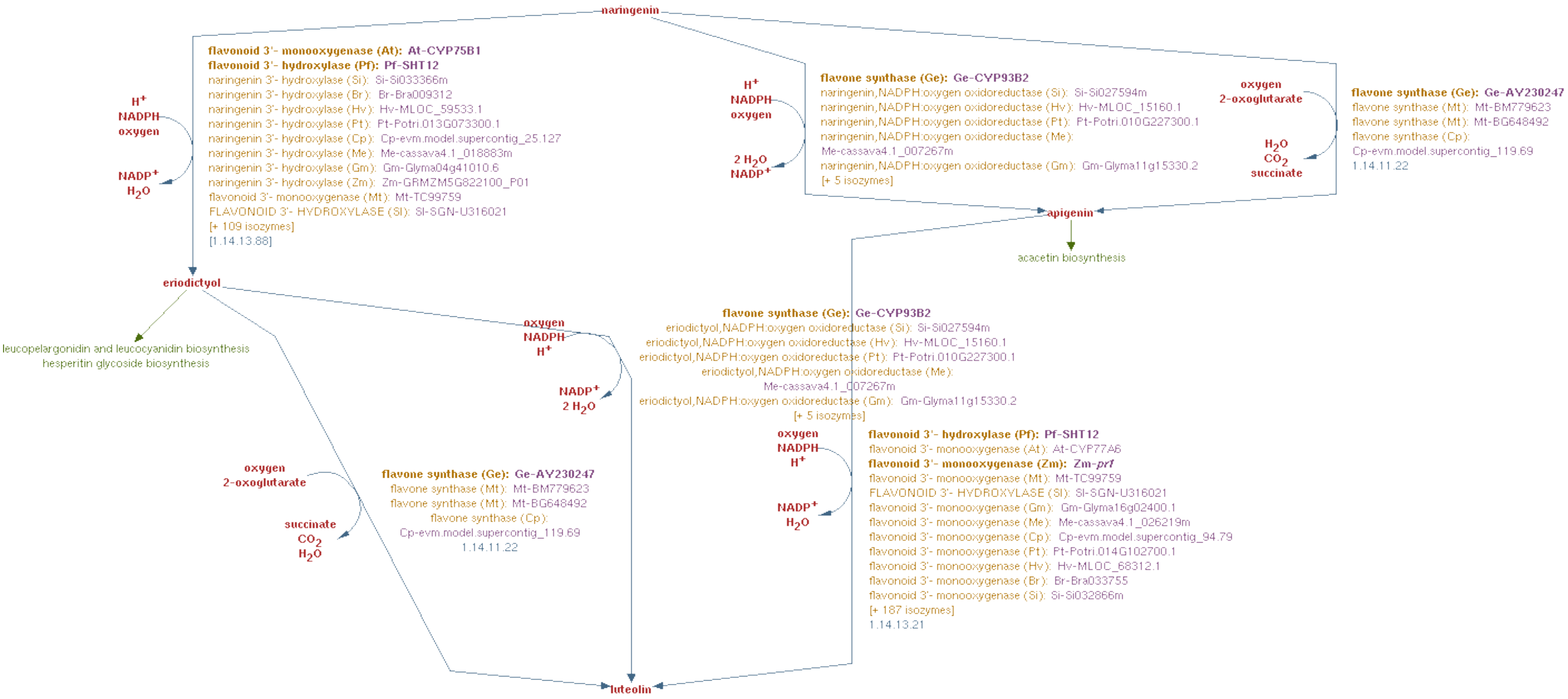
naringenin
flavonoid 3'- monooxygenase (At): At-CYP75B1
flavonoid 3'- hydroxylase (Pf): Pf-SHT12
naringenin 3'- hydroxylase (Si): Si-Si033366m
naringenin 3'- hydroxylase (Br): Br-Bra009312
naringenin 3'- hydroxylase (Hv): Hv-MLOC_59533.1
naringenin 3'- hydroxylase (Pt): Pt-Potri.013G073300.1
naringenin 3'- hydroxylase (Cp): Cp-evm.model.supercontig_25.127
naringenin 3'- hydroxylase (Me): Me-cassava4.1_018883m
naringenin 3'- hydroxylase (Gm): Gm-Glyma04g41010.6
naringenin 3'- hydroxylase (Zm): Zm-GRMZM5G822100_P01
flavonoid 3'- monooxygenase (Mt): Mt-TC99759
FLAVONOID 3'- HYDROXYLASE (Sl): Sl-SGN-U316021
[+ 109 isozymes]
[1.14.13.88]
H^+
NADPH
oxygen
$NADP^+$
H_2O
flavone synthase (Ge): Ge-CYP93B2
naringenin,NADPH:oxygen oxidoreductase (Si): Si-Si027594m
naringenin,NADPH:oxygen oxidoreductase (Hv): Hv-MLOC_15160.1
naringenin,NADPH:oxygen oxidoreductase (Pt): Pt-Potri.010G227300.1
naringenin,NADPH:oxygen oxidoreductase (Me): Me-cassava4.1_007267m
naringenin,NADPH:oxygen oxidoreductase (Gm): Gm-Glyma11g15330.2
[+ 5 isozymes]
H^+
NADPH
oxygen
2 H_2O
$NADP^+$
oxygen
2-oxoglutarate
H_2O
CO_2
succinate
flavone synthase (Ge): Ge-AY230247
flavone synthase (Mt): Mt-BM779623
flavone synthase (Mt): Mt-BG648492
flavone synthase (Cp): Cp-evm.model.supercontig_119.69
1.14.11.22
apigenin
acacetin biosynthesis
eriodictyol
leucopelargonidin and leucocyanidin biosynthesis
hesperitin glycoside biosynthesis
oxygen
NADPH
H^+
$NADP^+$
2 H_2O
flavone synthase (Ge): Ge-CYP93B2
eriodictyol,NADPH:oxygen oxidoreductase (Si): Si-Si027594m
eriodictyol,NADPH:oxygen oxidoreductase (Hv): Hv-MLOC_15160.1
eriodictyol,NADPH:oxygen oxidoreductase (Pt): Pt-Potri.010G227300.1
eriodictyol,NADPH:oxygen oxidoreductase (Me): Me-cassava4.1_007267m
eriodictyol,NADPH:oxygen oxidoreductase (Gm): Gm-Glyma11g15330.2
[+ 5 isozymes]
oxygen
2-oxoglutarate
succinate
CO_2
H_2O
flavone synthase (Ge): Ge-AY230247
flavone synthase (Mt): Mt-BM779623
flavone synthase (Mt): Mt-BG648492
flavone synthase (Cp): Cp-evm.model.supercontig_119.69
1.14.11.22
oxygen
NADPH
H^+
$NADP^+$
H_2O
flavonoid 3'- hydroxylase (Pf): Pf-SHT12
flavonoid 3'- monooxygenase (At): At-CYP77A6
flavonoid 3'- monooxygenase (Zm): Zm-*pr1*
flavonoid 3'- monooxygenase (Mt): Mt-TC99759
FLAVONOID 3'- HYDROXYLASE (Sl): Sl-SGN-U316021
flavonoid 3'- monooxygenase (Gm): Gm-Glyma16g02400.1
flavonoid 3'- monooxygenase (Me): Me-cassava4.1_026219m
flavonoid 3'- monooxygenase (Cp): Cp-evm.model.supercontig_94.79
flavonoid 3'- monooxygenase (Pt): Pt-Potri.014G102700.1
flavonoid 3'- monooxygenase (Hv): Hv-MLOC_68312.1
flavonoid 3'- monooxygenase (Br): Br-Bra033755
flavonoid 3'- monooxygenase (Si): Si-Si032866m
[+ 187 isozymes]
1.14.13.21
luteolin

2. Luteolin

3. Rosmarinic acid Pathway1

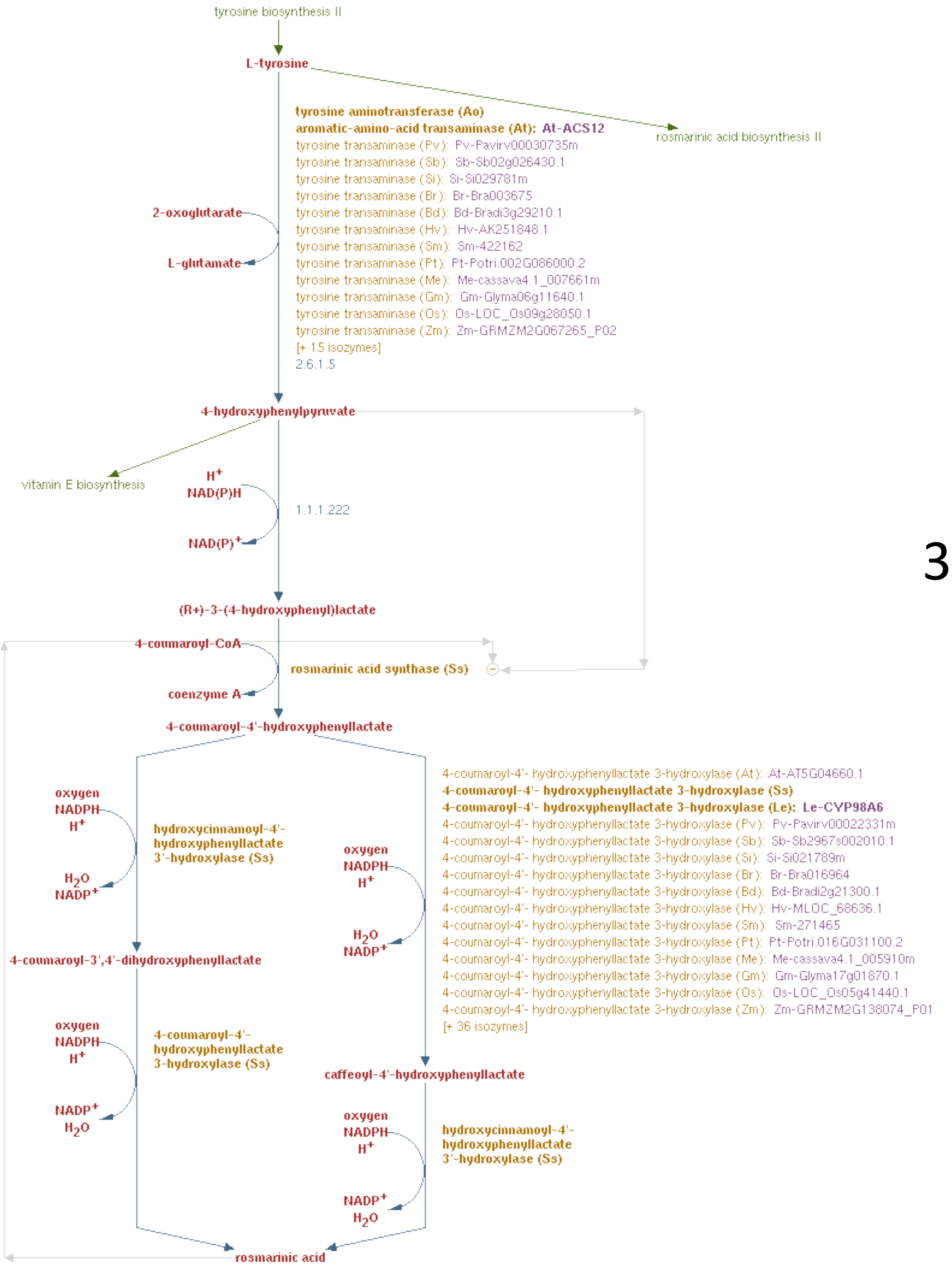

4. Rosmarinic acid Pathway 2

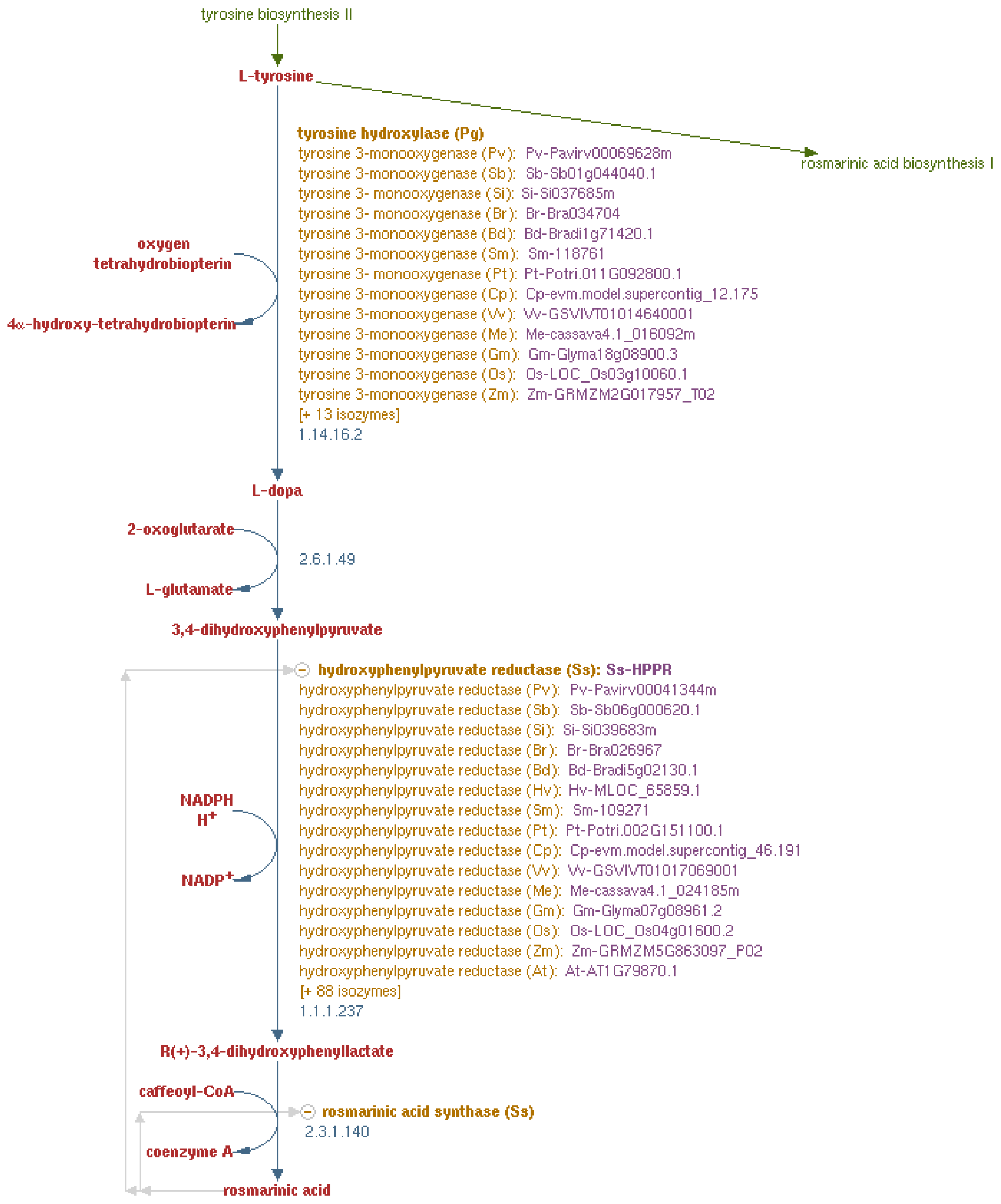

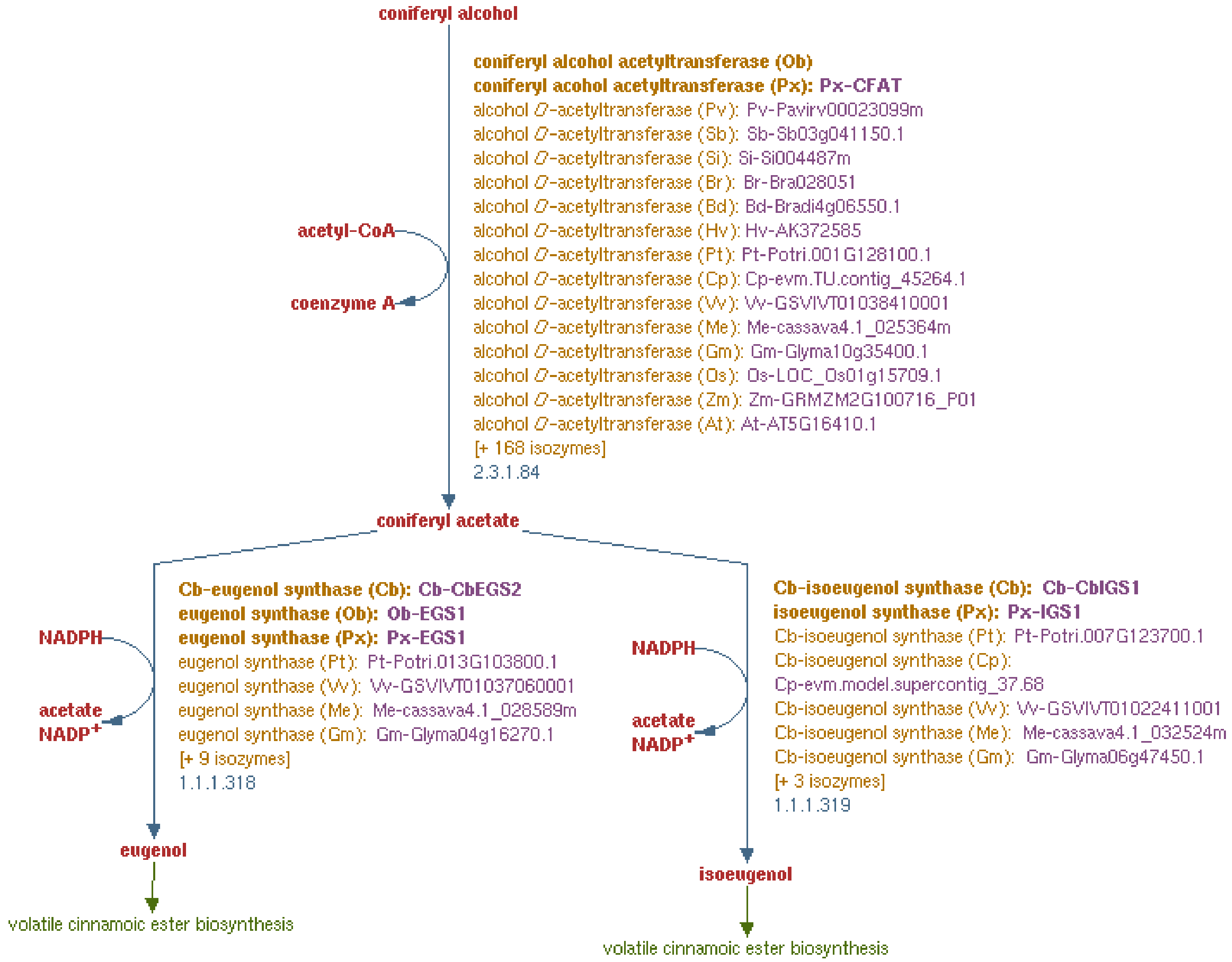
coniferyl alcohol
coniferyl alcohol acetyltransferase (Ob)
coniferyl acohol acetyltransferase (Px): Px-CFAT
alcohol O-acetyltransferase (Pv): Pv-Pavirv00023099m
alcohol O-acetyltransferase (Sb): Sb-Sb03g041150.1
alcohol O-acetyltransferase (Si): Si-Si004487m
alcohol O-acetyltransferase (Br): Br-Bra028051
alcohol O-acetyltransferase (Bd): Bd-Bradi4g06550.1
alcohol O-acetyltransferase (Hv): Hv-AK372585
alcohol O-acetyltransferase (Pt): Pt-Potri.001G128100.1
alcohol O-acetyltransferase (Cp): Cp-evm.TU.contig_45264.1
alcohol O-acetyltransferase (Vv): Vv-GSVIVT01038410001
alcohol O-acetyltransferase (Me): Me-cassava4.1_025364m
alcohol O-acetyltransferase (Gm): Gm-Glyma10g35400.1
alcohol O-acetyltransferase (Os): Os-LOC_Os01g15709.1
alcohol O-acetyltransferase (Zm): Zm-GRMZM2G100716_P01
alcohol O-acetyltransferase (At): At-AT5G16410.1
[+ 168 isozymes]
2.3.1.84
acetyl-CoA
coenzyme A
coniferyl acetate
Cb-eugenol synthase (Cb): Cb-CbEGS2
eugenol synthase (Ob): Ob-EGS1
eugenol synthase (Px): Px-EGS1
eugenol synthase (Pt): Pt-Potri.013G103800.1
eugenol synthase (Vv): Vv-GSVIVT01037060001
eugenol synthase (Me): Me-cassava4.1_028589m
eugenol synthase (Gm): Gm-Glyma04g16270.1
[+ 9 isozymes]
1.1.1.318
NADPH
acetate
NADP+
eugenol
volatile cinnamoic ester biosynthesis
Cb-isoeugenol synthase (Cb): Cb-CbIGS1
isoeugenol synthase (Px): Px-IGS1
Cb-isoeugenol synthase (Pt): Pt-Potri.007G123700.1
Cb-isoeugenol synthase (Cp):
Cp-evm.model.supercontig_37.68
Cb-isoeugenol synthase (Vv): Vv-GSVIVT01022411001
Cb-isoeugenol synthase (Me): Me-cassava4.1_032524m
Cb-isoeugenol synthase (Gm): Gm-Glyma06g47450.1
[+ 3 isozymes]
1.1.1.319
NADPH
acetate
NADP+
isoeugenol
volatile cinnamoic ester biosynthesis

5. Eugenol

6. Methylchavicol

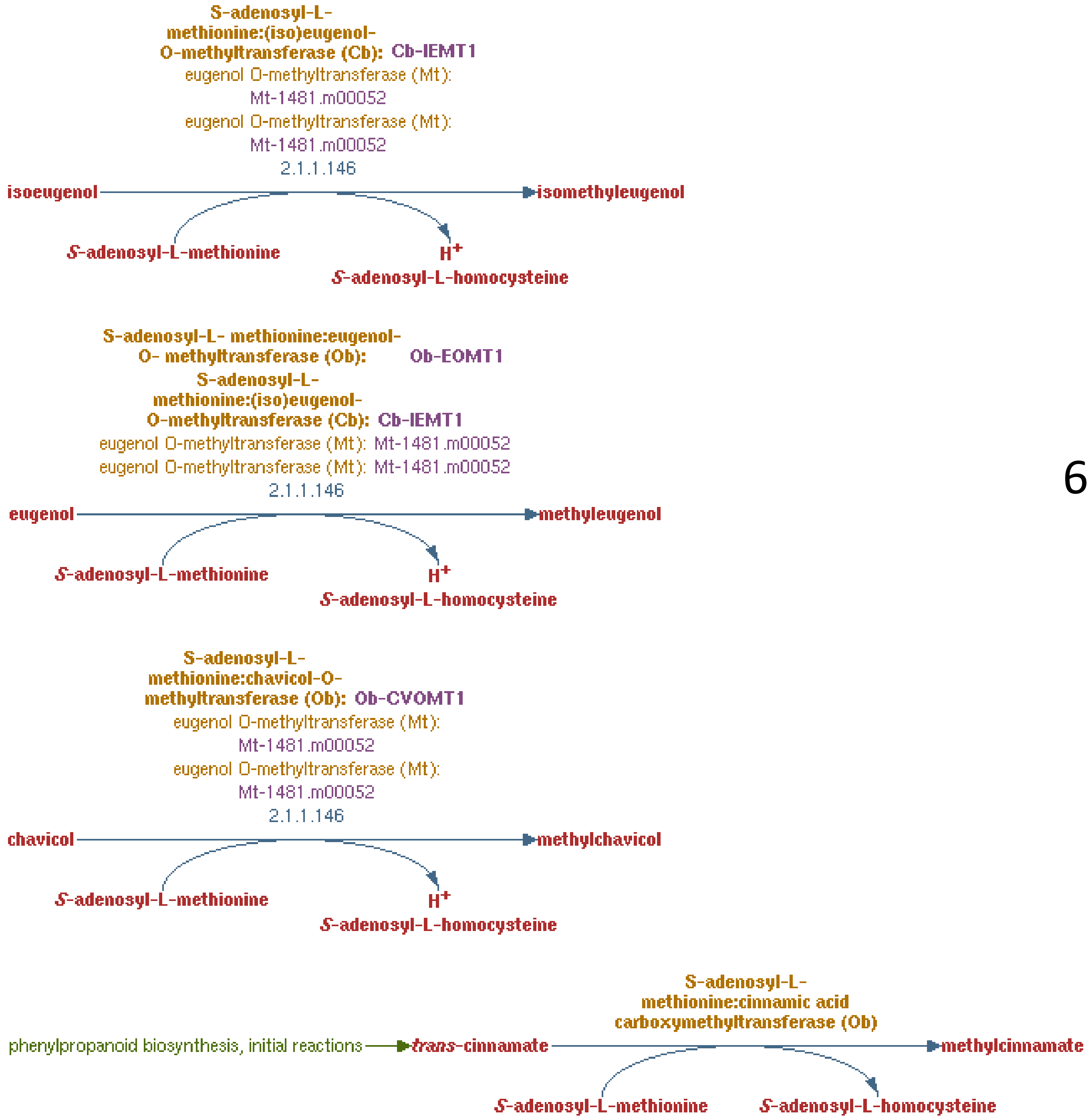

7. Citral

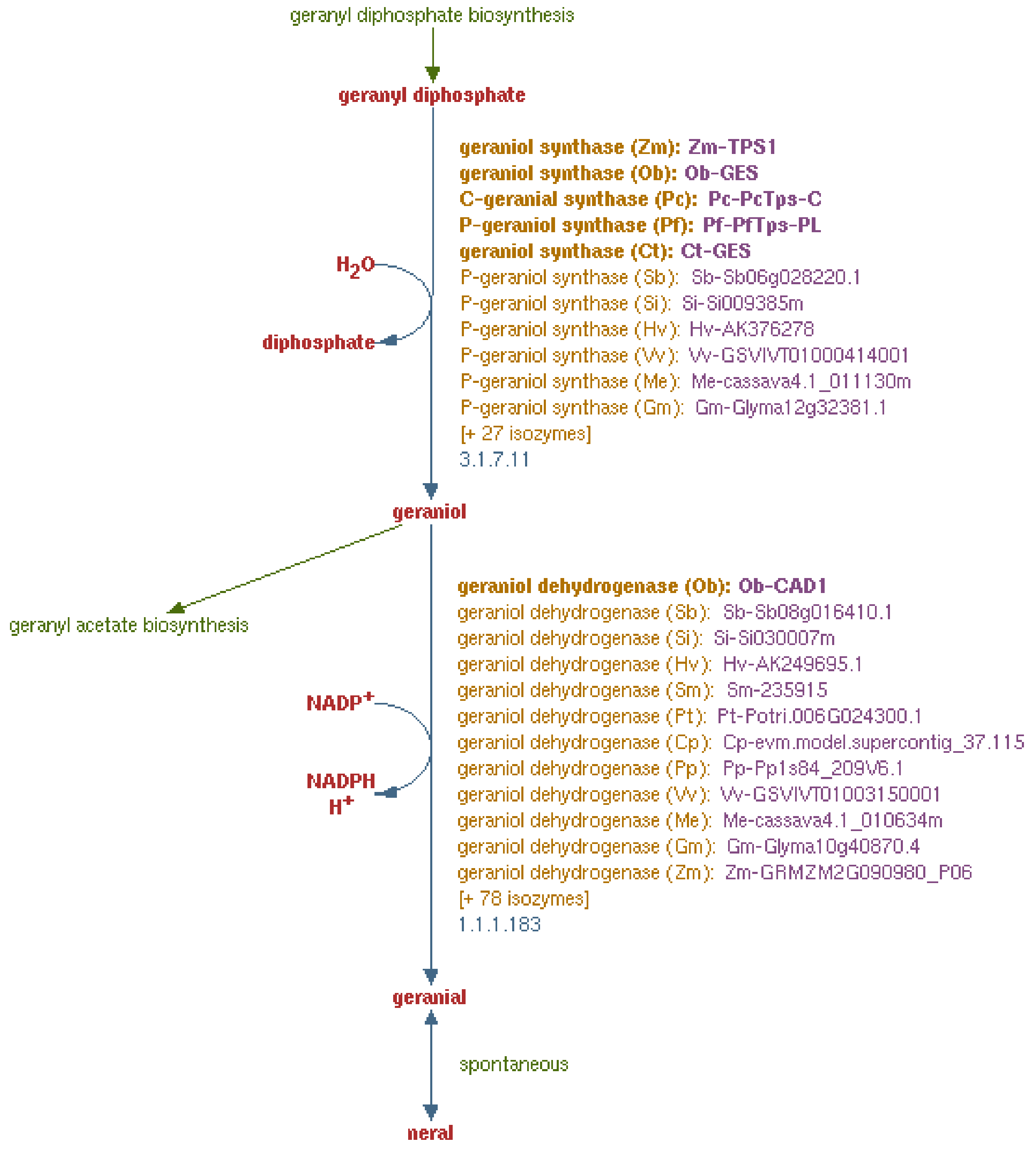
geranyl diphosphate biosynthesis
geranyl diphosphate
geraniol synthase (Zm): Zm-TPS1
geraniol synthase (Ob): Ob-GES
C-geranial synthase (Pc): Pc-PcTps-C
P-geraniol synthase (Pf): Pf-PfTps-PL
geraniol synthase (Ct): Ct-GES
P-geraniol synthase (Sb): Sb-Sb06g028220.1
P-geraniol synthase (Si): Si-Si009385m
P-geraniol synthase (Hv): Hv-AK376278
P-geraniol synthase (Vv): Vv-GSVIVT01000414001
P-geraniol synthase (Me): Me-cassava4.1_011130m
P-geraniol synthase (Gm): Gm-Glyma12g32381.1
[+ 27 isozymes]
3.1.7.11
H_2O
diphosphate
geraniol
geranyl acetate biosynthesis
geraniol dehydrogenase (Ob): Ob-CAD1
geraniol dehydrogenase (Sb): Sb-Sb08g016410.1
geraniol dehydrogenase (Si): Si-Si030007m
geraniol dehydrogenase (Hv): Hv-AK249695.1
geraniol dehydrogenase (Sm): Sm-235915
geraniol dehydrogenase (Pt): Pt-Potri.006G024300.1
geraniol dehydrogenase (Cp): Cp-evm.model.supercontig_37.115
geraniol dehydrogenase (Pp): Pp-Pp1s84_209V6.1
geraniol dehydrogenase (Vv): Vv-GSVIVT01003150001
geraniol dehydrogenase (Me): Me-cassava4.1_010634m
geraniol dehydrogenase (Gm): Gm-Glyma10g40870.4
geraniol dehydrogenase (Zm): Zm-GRMZM2G090980_P06
[+ 78 isozymes]
1.1.1.183
$NADP^+$
NADPH
H^+
geranial
spontaneous
neral

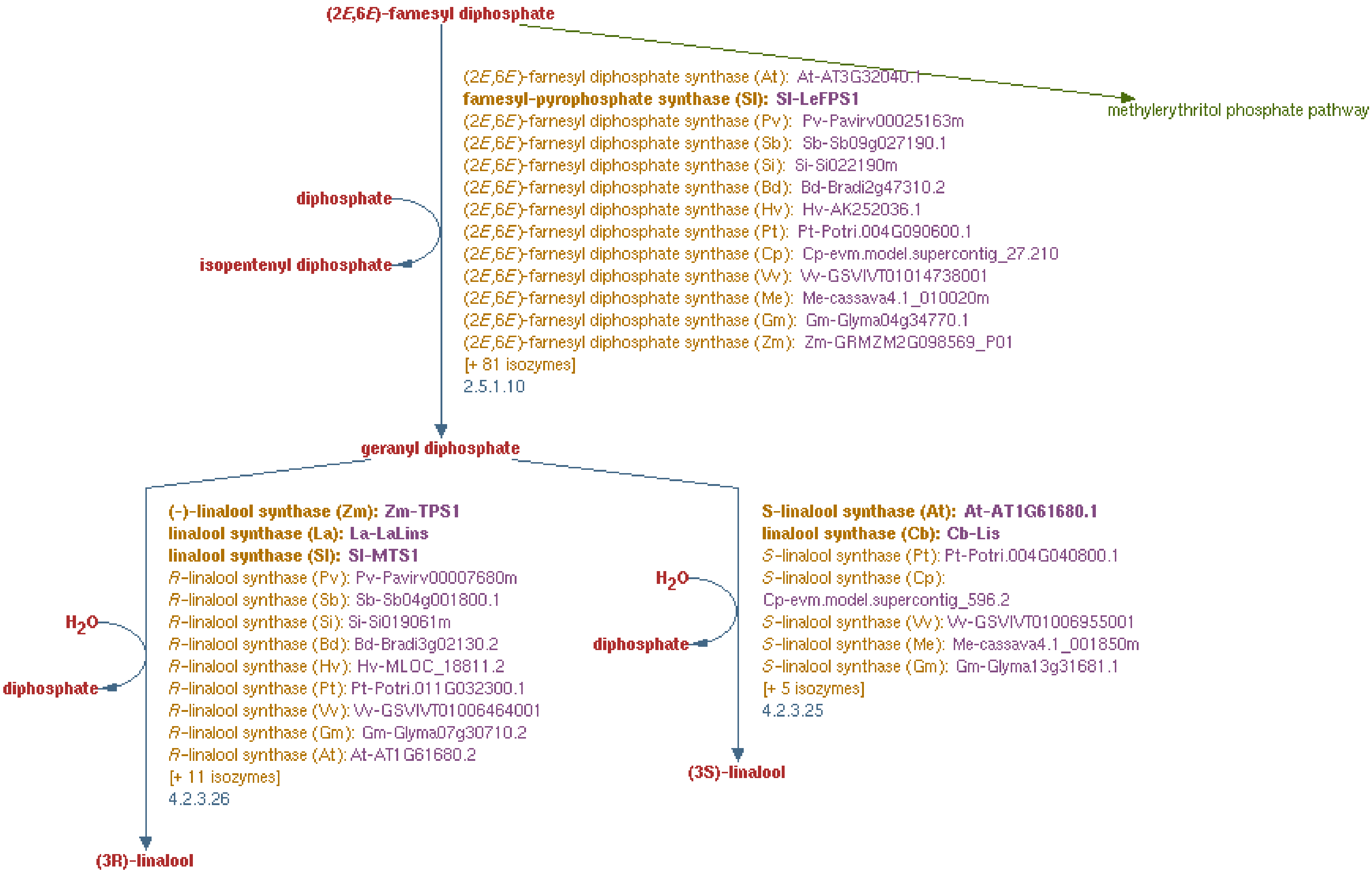

(2E,6E)-farnesyl diphosphate
methylerythritol phosphate pathway
(2E,6E)-farnesyl diphosphate synthase (At): At-AT3G32040.1
farnesyl-pyrophosphate synthase (Sl): Sl-LeFPS1
(2E,6E)-farnesyl diphosphate synthase (Pv): Pv-Pavirv00025163m
(2E,6E)-farnesyl diphosphate synthase (Sb): Sb-Sb09g027190.1
(2E,6E)-farnesyl diphosphate synthase (Si): Si-Si022190m
(2E,6E)-farnesyl diphosphate synthase (Bd): Bd-Bradi2g47310.2
(2E,6E)-farnesyl diphosphate synthase (Hv): Hv-AK252036.1
(2E,6E)-farnesyl diphosphate synthase (Pt): Pt-Potri.004G090600.1
(2E,6E)-farnesyl diphosphate synthase (Cp): Cp-evm.model.supercontig_27.210
(2E,6E)-farnesyl diphosphate synthase (Vv): Vv-GSVIVT01014738001
(2E,6E)-farnesyl diphosphate synthase (Me): Me-cassava4.1_010020m
(2E,6E)-farnesyl diphosphate synthase (Gm): Gm-Glyma04g34770.1
(2E,6E)-farnesyl diphosphate synthase (Zm): Zm-GRMZM2G098569_P01
[+ 81 isozymes]
2.5.1.10
diphosphate
isopentenyl diphosphate
geranyl diphosphate
(-)-linalool synthase (Zm): Zm-TPS1
linalool synthase (La): La-LaLins
linalool synthase (Sl): Sl-MTS1
R-linalool synthase (Pv): Pv-Pavirv00007680m
R-linalool synthase (Sb): Sb-Sb04g001800.1
R-linalool synthase (Si): Si-Si019061m
R-linalool synthase (Bd): Bd-Bradi3g02130.2
R-linalool synthase (Hv): Hv-MLOC_18811.2
R-linalool synthase (Pt): Pt-Potri.011G032300.1
R-linalool synthase (Vv): Vv-GSVIVT01006464001
R-linalool synthase (Gm): Gm-Glyma07g30710.2
R-linalool synthase (At): At-AT1G61680.2
[+ 11 isozymes]
4.2.3.26
H_2O
diphosphate
(3R)-linalool
S-linalool synthase (At): At-AT1G61680.1
linalool synthase (Cb): Cb-Lis
S-linalool synthase (Pt): Pt-Potri.004G040800.1
S-linalool synthase (Cp):
Cp-evm.model.supercontig_596.2
S-linalool synthase (Vv): Vv-GSVIVT01006955001
S-linalool synthase (Me): Me-cassava4.1_001850m
S-linalool synthase (Gm): Gm-Glyma13g31681.1
[+ 5 isozymes]
4.2.3.25
H_2O
diphosphate
(3S)-linalool

8. Linalool

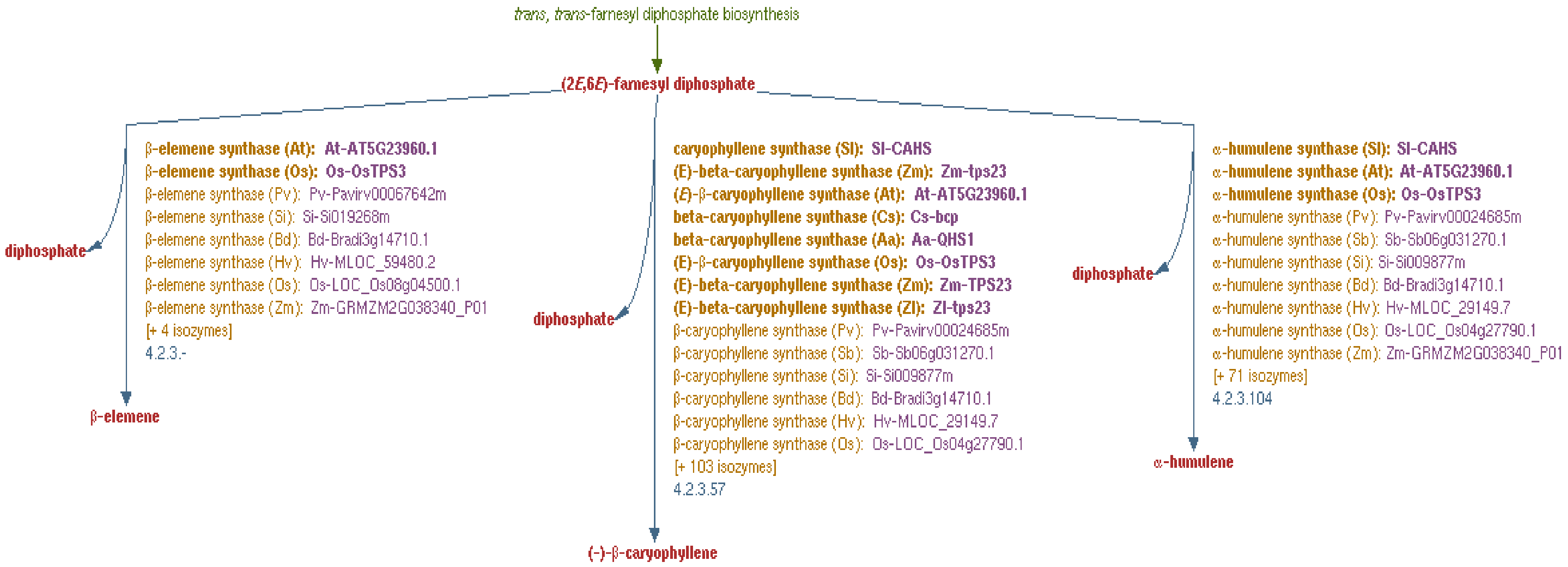

9. Caryophylene

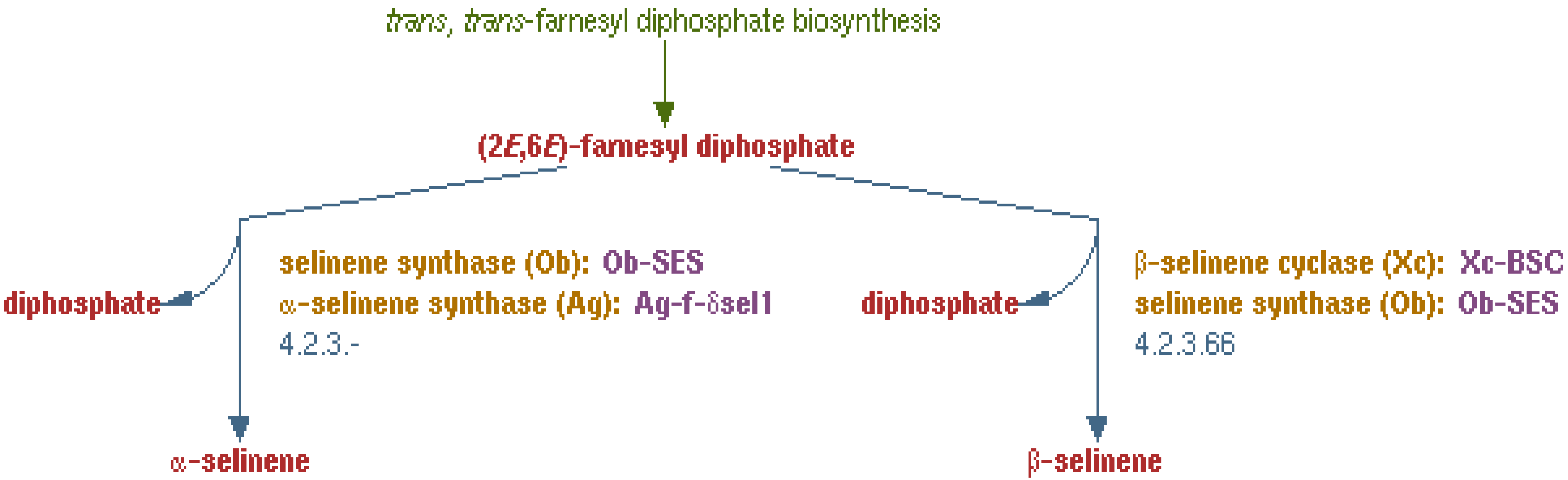
trans, *trans*-farnesyl diphosphate biosynthesis
(2*E*,6*E*)-farnesyl diphosphate
diphosphate
selinene synthase (Ob): Ob-SES
α-selinene synthase (Ag): Ag-f-δsel1
4.2.3.-
α-selinene
diphosphate
β-selinene cyclase (Xc): Xc-BSC
selinene synthase (Ob): Ob-SES
4.2.3.66
β-selinene

10. Selinene

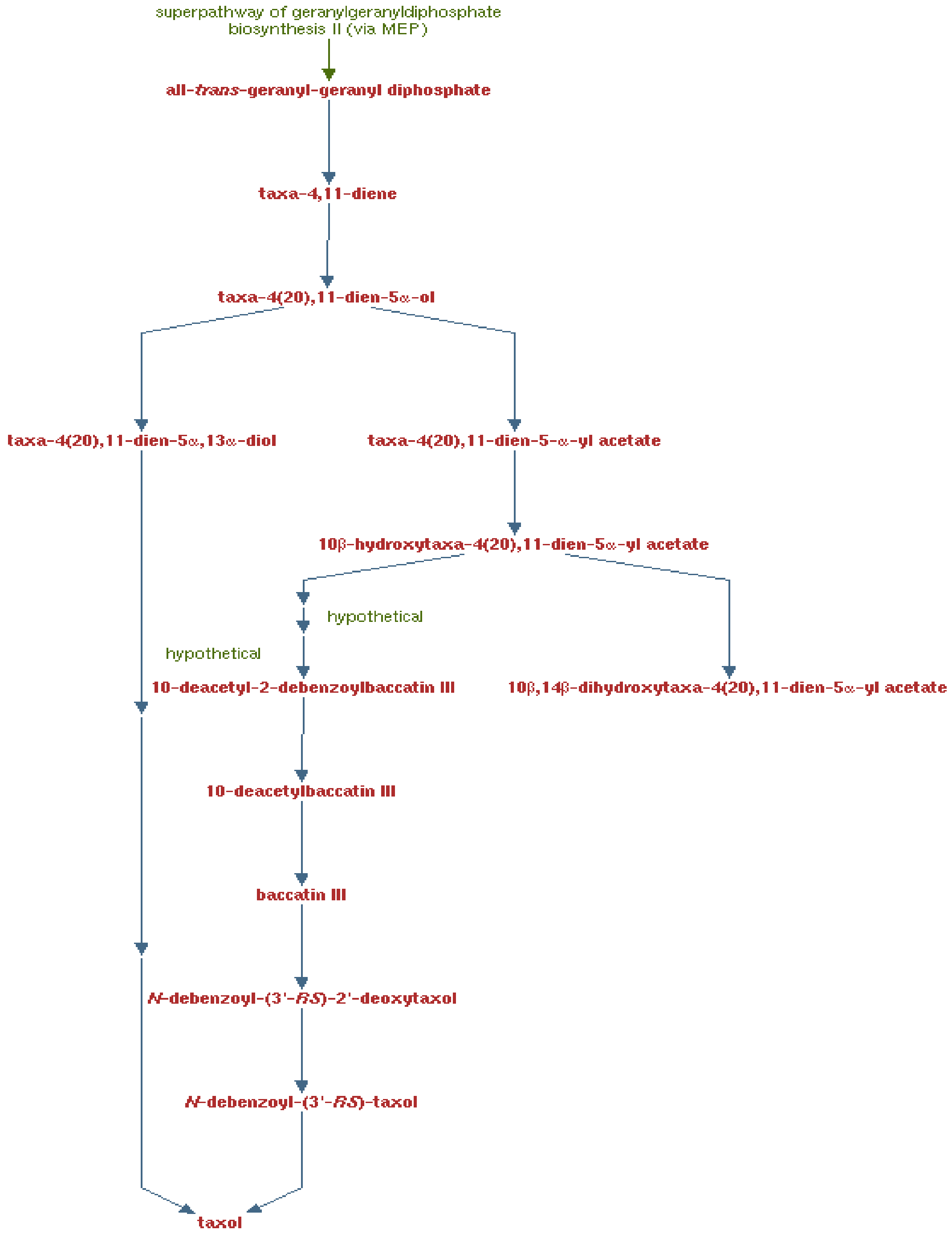
superpathway of geranylgeranyldiphosphate biosynthesis II (via MEP)
all-*trans*-geranyl-geranyl diphosphate
taxa-4,11-diene
taxa-4(20),11-dien-5α-ol
taxa-4(20),11-dien-5α,13α-diol
taxa-4(20),11-dien-5-α-yl acetate
10β-hydroxytaxa-4(20),11-dien-5α-yl acetate
hypothetical
hypothetical
10-deacetyl-2-debenzoylbaccatin III
10β,14β-dihydroxytaxa-4(20),11-dien-5α-yl acetate
10-deacetylbaccatin III
baccatin III
N-debenzoyl-(3'-*RS*)-2'-deoxytaxol
N-debenzoyl-(3'-*RS*)-taxol
taxol

11. Taxol

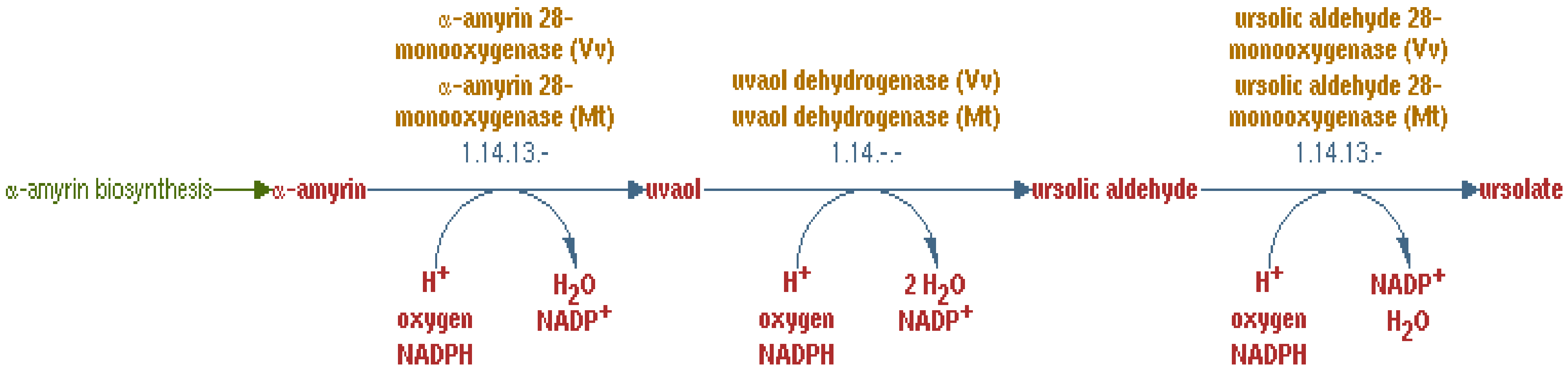

12. Ursolic Acid

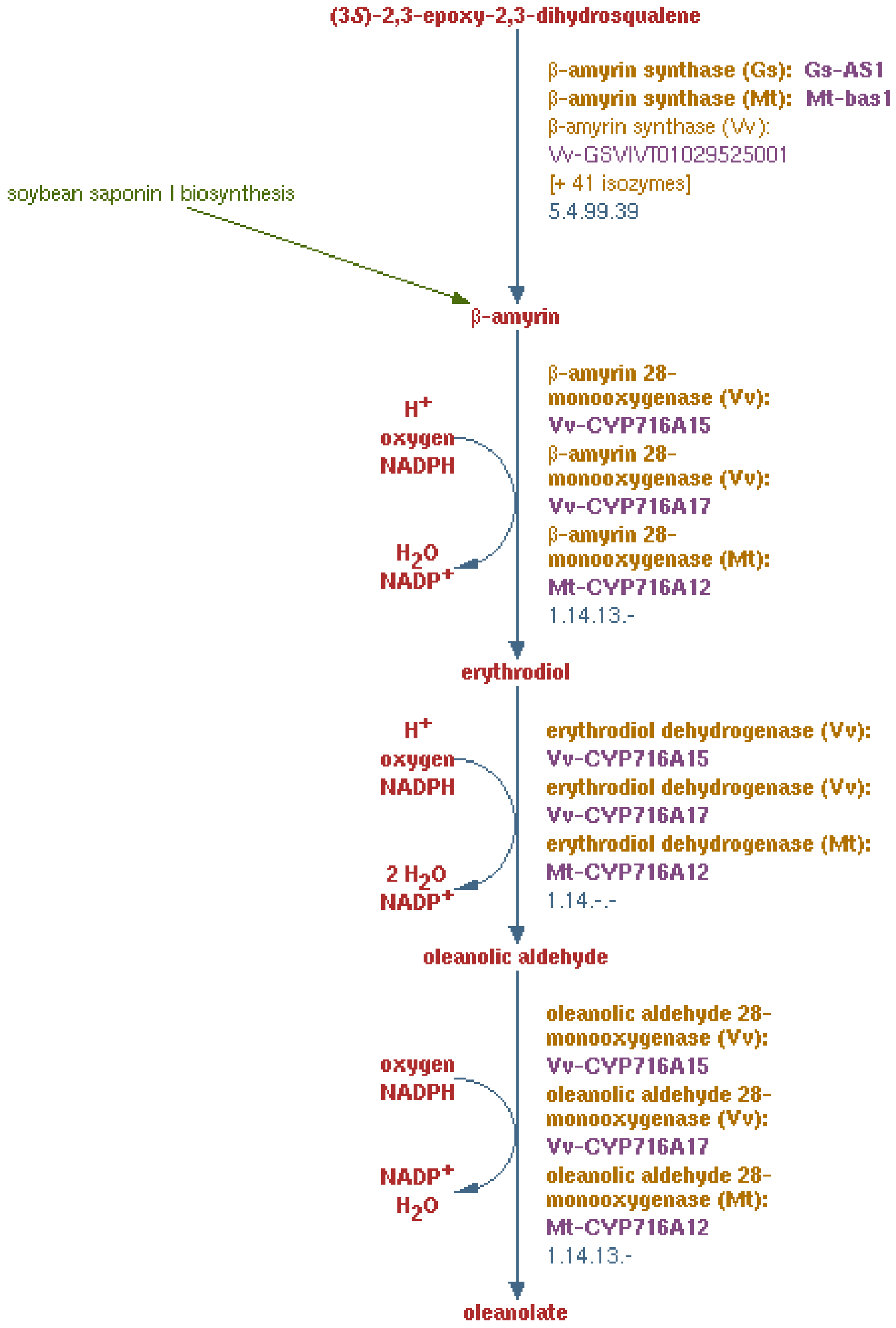

13. Oleanolic acid

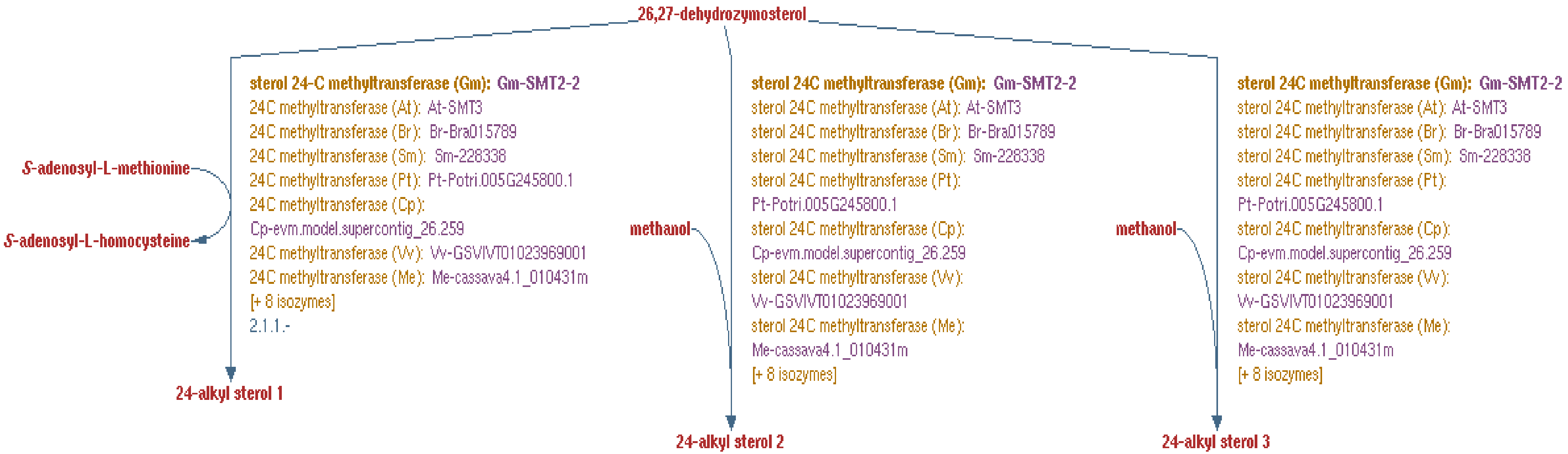

14. Sitosterol
