# Supplementary material for: Genome sequencing of herb Tulsi (Ocimum tenuiflorum) unravels key genes behind its strong medicinal properties
Source: BMC Plant Biol. 2015 Aug 28;15:212. doi: 10.1186/s12870-015-0562-x (PMC4552454; doi:10.1186/s12870-015-0562-x)
Supplement: Additional file 22: Table S10. — Sequences of putative terpene synthases in O. tenuiflorum genome. [file 12870_2015_562_MOESM22_ESM.doc]

**Specialized metabolites detection and validation**

Owing to remote homology between these enzymes, large sequence diversity of protein families (like terpene synthases) and high sensitivity of our sequence search methods, it is often impossible to pinpoint the actual enzymes working on the synthesis of a specialized metabolite. 50% of the genes identified were observed to code for enzymes involved in the biosynthesis of sesquiterpenes (caryophyllene, selinene, taxol, ursolic acid and oleanolic acid). Terpene synthases (Tps) are classified into six groups knows as Tpsa, Tpsb, Tpsc, Tpsd, Tpse and Tpsf owing to their high sequence diversity. Representative sequences of these six classes from the plant kingdom were used to search against Tulsi genome and a phylogeny is constructed to annotate Tulsi genes in different tps classes.

The identified terpene synthases were further analyzed for the presence of known functional motifs (such as RR domain and the catalytic DDXXD or DIDD motif) that are previously well-characterized in terpene synthases. This resulted in 22 gene products, which we label as putative terpene synthases in *O. tenuiflorum* genome (Supplementary Table 10). Multiple sequence alignment indeed confirms the presence of catalytic residues in these gene products (data not shown).

Further, these gene products cluster well with the known terpene synthase subclasses by phylogeny. Tpsa and Tpsb are the angiosperm specific subclasses of terpene synthase, and we observed that the *O. tenuiflorum* terpene synthases mainly cluster with these subclasses (12/22 *O. tenuiflorum* terpene synthases cluster with Tpsa; 4/22 *O. tenuiflorum* terpene synthases cluster with Tpsb; Figure 8). As an evidence of cross-validation, we noticed that none of the identified *O. tenuiflorum* terpene synthase sequences cluster with the gymnosperm specific subclass (Tpsd). In addition, we observed three hits in Tulsi genome (Ote100088210031, Ote100255640111 and Ote100245780031) that have significant homology to terpene synthases and cluster close to tpsc, tpse and tpsf members, bearing sequence similarity to copaloyl synthase family of terpene synthases. These hits are not shown in the phylogeny owing to their short length of gene products.

Supplementary Table 10: Sequences of Putative terpene synthases in *O. tenuiflorum* genome

| S.N0 | Putative terpene synthesis protein sequences from *O. tenuiflorum* |
| --- | --- |
| 1 | >Ote23728349000  MAAATPLEQMILIDTLERLGLAYHFESDIEHKLQQIYDDDVRGHECDLFATALRFRLLRQHRHRVSCDVFDKFVDKGGKFKGDAEGLLSLYEAAHVRFHDENILEEAERFTRHELSCMESQLERPLKDKVKRALERPLHREVPLIYARLFVSIYEKDDSMDEHLLKLAKFNFNFLQNLYRKELCDLSRWWNKFDLKTKLPYIRDRLVEAYLWGVGYHFEPQYSYVRKGLVLGIKIIGIMDDTYDNYATINEAQIFTEILDRWSMDEIDRLPDYMKIVLHFVMSAYEEYERDAEKNGKEFASPYFKET |
| 2 | >Ote237809130001  MQAELIEQVKMLLEEDMEAVQQLELIDDLDNLGLSYFFKDKIKHNLNIIYNHTKVFRMNEAEEEMDLYLAALGFRLLRKHGFNVSQGIFDCFKNEEGSDFKPRLAEDTKALLQLYEASFLLREGEDTLELAREFAAKYLQKKLDEGGDEIDDINLFRSIKHSMDLPLHWRIQRYEARWMLDAYARRPDMNPVIFELAKLDFNIIQATQEEELKYVARWWNSSCLAEKLPFVRDRIVECYFWALGLFEPHQNGYQRKMAAKIITFVTVIDDVYDVYGTLDELQLFTDTIRRFAVSTCSFVSEVAYDVLKDKGFIAFSHLQTSWLSLVEAFFKEAKWYYSGYTPTLEEYLNNSKISISSPTIISQVYFTLQNSSTDKTDIESLYKYHHILYLSGMILRLADDLGTTQFEMKRGDVAKAMQCYMKETNASEEAAQEHVRFLIRKAWKEMNTAMAAAECPFSHDLVEAAANLGRAAQFIYLEGDGHGVQHSKIHQQMADLLFYPYV |
| 3 | >Ote100138760001  MEMISAITNSPRRSANYQPNIWNYDYLISLTDNKHEELEKLEREAETLKEQVCSLFVGSKDPLHKLELIDLFNKLGISHFFEEEVRKSLEEMHHAESLMMMEDDLYSAALYFRILRQHGYHVSQEAISRLLDDKENSWDEKTRVEILEASHLVAYDEDMLDKKFSGVDVSSATSVNSNCPSHRSVSWFNVSSHMHANSNPIILRLAGLSFKAMQLHHHRDLNHILRWWRKVEALEFVKFSRDRVVESFLSAVGVAHEPQHTSLRKWLTKAICMVKEKGGSSVLPHLRKAWTGFCKALLVEAKWNEMGVTPSLEEYLDNGWASSSGQVLCLHVLAGVGDDMADTLGLADHEIIYHTSLIFRLCNDLATFKAEAERGDSPSSIACYMKEANATEKEAREHIKELVSESWKKINGAWMRCPKSQQKMMRHAVNIARVANFLYQNGDGFGXSRSRDQEQSFIMLDPPFAACSTTPLEFYGQPNKYGILNVDSAL |
| 4 | >Ote100212320011  MDEADQLPDYMKTTLQFIMSAYEEYERYAEKNGKQFASPYFKETIQQLGRAYNQELKWIMENQMPPFQDYLKNSEITSCIYILFASVMPGLKSLTQETIDWIKSEPSFAVKAGLIGRYWDDIGSHRREEKGGETLTVIDCYMKQYGVSKQETISRFSDEIEDLWKEVNKEWVVTTSVPREIAVQFVNYSRLCDATYNKNNGDGYTDPKLSALSIRALFLDPIVIS |
| 5 | >Ote100219520011  MLHKEELDVVSRWWKDLDLVAKLPYARDRVVECFFWAMGVYHEPQYSRARIMLTKTIAMTSIIDDTYDAYGTIEELDVFTEAIERCGLTLISQGSQILKYLIYHPLKELVRSYHLEAKWFIQGYLPPFEEYLKNALITCTYCYHTTTSLLGVESAMKEDFEWLSKKPKMLVASLLICRVIDDIATYEVEKDRGQIATGIESYMKENCATKEEAVAKFFEIATDAWKDINEECMRSSPYSRDVLMRILNLERIIDVTYKGNEDGYTQPEKVLKPHIIDLFVDPIEN |
| 6 | >Ote100051830001  MLLQRQMEAVPLLELIDDLQRLGISYHFLDEINQVLSCIYLDNFYAEERDLYSTALAFRLLRDHGFQVPQDADSLENSRLFIEAYGENPQINPTLLQLAKLDFNILQAIHQQELTLLSRWWKDTGLAKMLPFVRDRTVENYFWTLGRLFQPEYGYSRMMATKVNVLITIIDDIFDVYGTWEELQLFNTVVERWDAHAIDQLPDYMKMCFLTLNNFVNEMAYDVLKDQGYASQYTPTLKEYMSNAWIWVSAPYEMERGDVPKSVELYMKEAGASREEAREHVEHMIWETWKKLNEERARD |
| 7 | >Ote100088210031  MYNSVRGQATDTLEVVVSEKIRQLREKIEWMLQNMDDGEISVSPYDTAWVAMVEDINGSGHPQFPASLRWISDNQLADGSWGDRKFVVYDRILNTLACVVALTAWKMHPHKCNKGLTFIRNNIEKLGHENEELMPIGFEVVFPSLIEKAQKLGIEIPNIDSPCIQKIQAMRDYKLRKIPMELLHKKPTSLLHSLEGMQGLVWQKLLDFRSDGSFLCSPSSTAYALHHTKDELCLNYLLKAVKKFDGGVPNVYPVDMFEHLWCVDRLQRLGICRYFRIQIKESLDYVYKYWTDKGICWARNTNVQDIDDTAMGFRLLRMHGYDVSTDVFEQFEKAGEFCSFPGQSTHAITGMYNLYRTSQVMFHGEHILEDARNYSANFLHKRRLANELVDKWIITKDLPGEVGYALDVPFFASLPRLEARFFIEQYGGDEDVWIGKTLYRMPYVNCDAYLELAKLDYNHCQTVHQLEWKSIQKWYKDCNIGEFGLSERSLLLTYYLAASTAFEPERKGERLAWVKTAVLVEAIMCQQLSQEQKREFVDEFENCSILKIENGGRHKTRIRLAEILISTVSQLSFEILVAQGRDIKQQLSNKWQKWLKTWEEGGDLGEAEAQLLLHTLHLSSGLDESSFSHQKYHELMEATSKVCHQLRLFKNRKVREQAIHFSSKHLGSSQVDDAQGCVSDLVIGTTFQIEANMKKLVKLAFTKSSRDLDSITKQSFFAIARSFYYTAYCDAGAINAHIYKVLFQKID |
| 8 | >Ote236375020011  MGVYHEPQYSRARIMLTKTIAMTSIIDDTYDAYGTIEELDLKELVRSYHLEAKWFIQGYLPPFEEYLKNALITCTYCYHTTTSLLGVETAIKEDFEWLSKKPKMLVAGLLICRVIDDIATYEVHQIILYLF |
| 9 | >Ote236417980011  MPPFSDYMANGFITSTYFLLAASSFMGITSASVEAFDWLMKKPRIQVANVTICRVIDDVATYEIEKERGQSATGIECYMKDNGVSEEEAMQEFKNIAENAWKDMNGEMVKEKSVSMEILKRIINLSRLIDVVYKNNQDGYTHPEKVLKPLITALLLHSFQS |
| 10 | >Ote237654730021  MESQSTVAYVKNLEETRRSAKFHPSIWGNHFLSYNSEKTEITVAENEEHAKQKEMVRSLLARVPDDSTHKMELIDAIQRLGVEYHFEKEIEEYLKYIHVEQNCKDRDVDAVSLCFRLLRQGGYNVSSDVFNKFIDNKGNFVESLKDDVKGLLNLWWNSLDIANKMPYARDRLVECFFWMVGVYFEPHYSTARTILTKVIAISSIIDDTYEYATLDELQTLTDAIQGWDNNMAMEDWPPYLQTCYKSIVDTYAGIEEQMEKEGKSYRVEYAKQELKKLVMAYFEEAKWLYRDYVPTAEEYMKVSLYEQKTASVQCYMKQYGMSKDQARDELRKQVKNAWKDMNQECLEPRPASMQILMRVVNLGRVINLLYADDDCYANPIKSKEWVKMVLLDPMDI |
| 11 | >Ote238047470061  MDITSSSRPLANYHPNIWGDRFLVYTPESHTGGEEELVEKLKKEVKLELIDALNDDVKLLKLVDAIQRLGIEYHFEEEIDHALQNFSEKFDHFSRKNSDLFTLALAFRLLRQHGYRVSCSIFEQFKDGEGGFKATEVLAVLEFFEATHLRVHDEDVLDEASIFARNFLESALPSLSNPLAEQVHHALHEYSNRRGLTRVEARHYIPIYGQFESHHQDLLKLAKLDFNILQSMHKRELSEVYRWWKDLDVPTNFPYARDRMVETYFWIMGVYFEPKYALARKILVKLQSLASIMDDTYDAYATFQELQLFTQAIQRDYFVEIKWREEKYKPRSEEYMQVATASCAYTSLIIISFVGMGDCVRKEDFEFVLSQPNIVRAALAICRLTDDLVGHEFEQEREHIPSWVECYTEEHKISKSEAVSEFKNRIESSWKDINEAFLDPTQIPTSQLYRILNFTRVIEVIYSKGDWYTHVGPQMQNLISQLLIQPIP |
| 12 | >Ote100123880021  MMTDVRPPMISFKPSMWGDTFSTFIFDEKVQQKYFEEIETLRKEVRSMLMAATSTKLMILINKLERLGLAYHFQTEIENKLNQFYVDEKDEVEDYDLFQTALRFRLLRQHQYHVSCNDFNKFFDEENKLKETLYSDVAGLLSLYEAAHVRIHDEHILDEALAFTSHHLSCMMPELEAHLKEQVQHALEYPLHRSLTVLHIRFRILSFEEDESRDELVLRLAKLNFNYLQNIYKNELVQVSEWYNKFDLKSKLPYARHRLVECYTWGVAYHSKPRYSYVRMALAKTYQMLTIMDDTYDNYATIEELEVFTRALESVSEEFGRDADKAEKSYAIPYYIEGMKQLSRVYYEEQKWIMERKLRTFEEYTNISQIICLLFVALISVIPGLKSTNEETIQWLLSDSQFIYSAGLAGRLLNNLASYKRKRLFQEEALSSFIKIIEDKWKHINAEWVKSRVVPNEMVEPLINLLSSAEMTYKNNEDGYSMPEGNSNWAQFISVLLVDFPLLT |
| 13 | >Ote100166970011  MPLHWRVQRLEARWYIQFYECGSDANPSLVELAKLDYNMVQATYQEELKRLSRWYEETGLPEKMSFARHRLAECFLWSLGFIPEGQFGFGRENLAKIGVLITILDDVYDIYGTLEELQVLTEIMERWNINLLDRLPEYMQICFLAVFNSANELAYDILRDQGINIISNLRKSWAGLSRSYYKEATWFHGGYIPTTEEYLNVSYISISGPTLLFYGYFTTTNPINKHELESLERLARSPSMVLRLADDLGTSSDEVERGDVSKSIQCFMNETGCCEEEARQHVKRLIDAEWKKMNRDILMEKPFKSFGSIAMNLGRIALCFYENGDGFGLPHSETKKKMVSLVVHPIHMP |
| 14 | >Ote100181330041  MEVYRSLDVTPLTTDETLFEKVEQVRDVLESVEQDSLESLELIDTIQRLGLCYYFHDEIQALLRHQISNDSDDLYQASLRFRLLRQQGFQVLAGELADETRGLMAMFEASHLNTGGEEILYGAAIFSSRHLNDMIMRCIDEEQVKMAKHCLVYPQHKSLARLTARIYLRFLDGKHPWENLLRDLAELEFVSTELLHKQEILQVARWWRGTRLGNELKSSRNQPLKWHMWSTATLIRSADWSKHRLLLTKSISLVYVVDDIFDLYGEIHELSLFTEAVIRWDASVTEKLPSYMKTCFNAIYDTTNEVSSFVSEEYGWNPIHFLIKEWGSLMEAFLIEGKWFRSVECAKSDEYLKNGVISSGVPMVLSHLFCLMGDPLTIQNQTLLNDPHGLTHSVAKLLRLLDDLGSAQDEQQEGYDGSYVECCMREGRVESLEGAREHVMGMVWETWENINKLGLCSTSPFSACFRHACLNAARMVPTMYSYDQNHRLPLLKHYVKSMFHDATLAKSILG |
| 15 | >Ote100193240141  MAICNFPSTPLLLFPPRIPIFLAINPKRKSLRAAACNTTSHKNWNISKDFESQVKGHTFSVAFLLLSYFISSMFPNFYISTFPSMQEDFWADYKGKMEEMKQVLLQHSNLEEKNNRKDSLILVDGIQRLGLDHHFDEEIDMILGKLSNHNFDSVEHDLYDVSLHFRLLRNHGYHVSSDVFNNFKGNGGKFEAELRQDIQGLMELHEAAQLGFTDEDIIDEAENFSRVNLNKCLEDTKVDDYHKKMIKNTLRHPQHKNIARLTARNCMNGGLVKGCSNKRWGKTLTEFAKMDASMGELMHQEELLQVSKWWETVGIAEGLSRARSQPVKWYIWSMAMLIDSPSLRVERIELAKVIAFVYLIDDIFDLYGTTDELSLFTQAINRWEYSASDTLPEYMRKSYKGLLDTTNGIAQKVEDNYGTNPIDSFKVAWISLCSAFMLEARWFRSSELPRAKEYLENGQVSTGAHVILVHLFFLLGLGRGWSSLHLKDTSTLLSSVATICRLSDDLGTAQDEQQDGSDGSYLKCCMNDEAKMSRKEAQQHMESMISNQWKILNKECFLHLNDSSVTCFRRASLNSARMVPLMYTYDQNKRLMLLEQLVNTTKLFH |
| 16 | >Ote100217510041  MKFVDRQNFIPPSGEIIFSPIIRIKRYEITVAENEELAEQKEMVKKLLGRVPDDSTHKMELIDVIQRLGVDYHFEKQIDESLQHIHGNYTQHYSXXXXXXXXXXXXXXXRQGGYNVTCGGTSWNPRGRILDRAIEFCSAHLQASLHQMSNVTLFEHVEEALKMPIRNSLTRLQAKKFMLMYEKDESRDEIVLNLAKLDFNIVQKMHQRELCDITWWWKSLDVANKMPYARDRITELFFWTVEVYFEPHYATARRILTKIIAMASIIDDTYEYATFDELQILTDAIQCWDSNVVLEDSPTHIQTCFKILMDTYTDIEDEMENVGEKYRVQYSKQDMKKLVMAYFEEAKWLYGDCIPTVEEYMKYEQKTSSVHCYMKQYGVSKDEAHEELRKQVQNAWKDMNAE |
| 17 | >Ote100245780031  MSVEDFNRCQDIHREELKELERWVIENRLDELKFARSKSAYCYFSAAGTIFSPELSDARMSWAKNGVLTTVVDDFFDVGGSMEELKNLIHLVELWDVDVSTKCTSPNVQIIFSALKHTICEIGDKGFKLQGRTAMVARHLKQTNNEIRHVQQLTVLGWLDLLYSMMKENEWSRDKFVPTIDEYISNAYVSFALGPIVLPALYLVGPKLSEEMVNHSEYHNLFKLMSTCGRLLNDIRSYERELKDGKLNALSLYMMNNGGEVTMEAAVSEVRSWIERQRRELLRTVLGKTVVPEACKELFWHMCTVVHMFYSKDDGFTSQDLLGVVNAIIKEPLVINDS |
| 18 | >Ote100255490031  MMLVLRQKKEMENLGRAYAVQYSKQEVKKLVRAYFEEAKWLYNDYVPTVEEYMKVSLVSCGYMALSTSCLIGTGDLVTKQDFDWISNKPLIVRASTVICRLMDDLVGDKYEQKPMAVYCYMKQYGASKDEAYRELREQVKKAWKDMNQECLEPRPASMEILTRIVNLARIMNLLYAEDDGYTDPVKSKEWVKMVFVDPIVI |
| 19 | >Ote100255640111  MSLASTLPFXXXXXXXXXSASHGAAFCAPPPPRSSAGHSSFSLSHAHTLLHARTRENGCLPLFRPLTPTSSLQCTALSRPRTEDYIDVIQNGLPVIKWHEIVENDAEKDSLKKETTSDKIRELANVIRSMLQSMGDGEISISPYDTAWAALVEAGGGGRRRPQFPSSLDWISKNQFPDGSWGDKTFSIYDRIINTLXXXXXXXXXLRSWNIHPDKTDKGILFIRKNMIRIDDENLEHMPIGFEVALPSLIEKARKFGIDIPNDTKGLREIYARREIKLKKIPREILHQVPTTLLHSLEGMSGLTWKKLLKLQSEDGSFLFSPSSTAFALQQTRDDNCLNYLTKHIHKFNGGVPNAYPVDLFEHLWAVDRLQRLGVSRYFKPEIEECIAYVHRYWTDKGICWARNSQVKDIDDTAMGFRLLRLHGYEVCADVFENFKSGGEFFCFQGQSTQAVTGMYNLYRASQLMFPGENILEDAAHFSANFLQLKRANNHLFDKWIITKDLPGEVGYALDVPWYASLPRVETRFYLEQYGGDDDVWIGKTLYRMPYVNNNKYLEMAKLDYNNCQALHQKEWRNIQKWYRSCNLGEFGLSERNLLQAYYIAAASVFEPERSQERLAWAKTQILMETIMLHFDFQQLSREQRRAFLDEFQHGSMLKYTNGGRYKTRSSLVGTLVRTLNQLSLDILLAHGRDIHHPLKNAWCKWLKSCGEGGDAELLVATLNLSGGGRRHTWPLESSNPKYEELLKATIGVCDKLRMFQRRKEHDGNGCFSIAGGITTTEIELEMKELVKLVLTENLHSEINQNFLIVAKSFYYAAYCNPGSINFHIAKVLFERVL |
| 20 | >Ote100273630011  MSTISMHVAILNKPAPNFLHNLNNKSSNPQRVPSTRRRPSCSLQLDVETRRSGNYQPSAWDFNYVQSLSNHYYKDEIKIILNSIYNHHKCFHNNEQGEKENADLYFTSLGFRLLRQHGFKVSQEVFDCFKNEEGTDFKASLGDDTKGLLQLYEASFLVREGEDTLEMGRQFSTKILQKKVDLAELIEEENLLSWIRHSLELPLHWRIQRLEARWFLDAYATRPDMNPIIFELAKLEFNVFQGVQQEELKDLS |
| 21 | >Ote100276590011  MYSISMHFGILNKPTPNFLHKRPSMPPRVSSLSAASTTTRCRTSCSLQLDAKPTDNTRRSGNYQPSAWDFSYIQSLNNHHYKPFLYLETCPIEEERYLTRQAELIELVKMMLEDEMETVEQLELIDDLKNMGLSHFFNHQIKQILNTIYNEHKRFHSNEGVEEKDLYFVALGFRLLRQHGFTVSQEVFDCFKNDKGTDFEPNLGEDTKGVLQLYEASFLWREGEDTLELGREFATKILQKKVEEGGDDNLLSSIHHALAIPLHWRIQRLEARWWWNSLGFVEKLPFARDRIVESYFWAVGTFEPLQYEYQRKQVAKIIALVTVIDDIFDVYGTVDELQLFIDTIRRWDTESINQLPYYMQLCYLAVYNFASETAYTILRDKGFMCISYLHRSFLDLVEAYVEEAKWYHSGYTPTLEEYLKNASISVTCPAVISQIYFTLVNSIDKPVIESMYKYHEILVLSGLLLRLPDDIGTAW |
| 22 | >Ote100033670041  MATSSGIVSCLSQVRPPVTSFGPSMWNETFSNFSFDEKVQKKYEETIEELKQEARGMLMAAATPTKQMIFIDALERLGLAYHFEIEIEQKLQEIYDEHVNANDCDLFIASLRFRLLRQHQHCVSCNVFDKFVDKEGKFEETLRNDTEGLLSLYEASHVRFPDEKILEEGERFTRHELRCVESELKSPFKERVKRALEHPFHRDVPIFHARFFISIYEIEPSMDELLLKLAKFNFNFLQNLYKKELYELFRWNKDEADKLPDYLKIVYDFITSTFEDYEHDAAKQGKEFAIPYLKETIQQLGGAYNRELNWVMKRQMPSFREYSKNSETTSCIYIISAATIPGLKSLTKEAIDWIKSEPKLALSAAMVGRFWNDIGSHERENKGGEMLTAVDCYMKQYGVSKQETMSKFAEFVEDKWKDINKEWATTTSPHNETKVQFLNYARMADACYNKNNGDAFTDPKIFKIFVGFESSAMHFAQWGRCGAFHSITLCILPFDIVHPVKWHLRPPTVAYKPSMWGDVFSNFHFDNKLQEKNVEPMEELKEETRSMLMAATSTKLMILVDKLERLGLAYHFQTEIEDRLKQVFDSPEEEDDHDLFATALRFRLLRQHQYNVSCNVFNKFVDEDGKWKETLSSHTEGILSLYEAAHVRIHDENILDEAVAFTVHHLTRMLPKLDPFTKEKVQQALKQSIHRGLPLLTFRFYISLYGRDGSTDEFLLKLAKLNFNFLQNIYRQELAELTRWWDKFDLKTKLFYARDRIVECYLWGNALRYEPRYSDLRLAVAKNMQLCSIMDDTYDNYATLEEDDLFTDILERWSLDEIDVLPDFMKVVYRFIMSLYEDYEREAEKQGKSFAPAYYREAVKQLGRAYNREQKWIMERRMPSFDEYMKNSVITSCIYLMFTAFIPSMKSVDEDAVKWLLSEPKIVISTAKMGRSMEDLGSHERENREGKLPTVVDCYMKDKGISKQEALSDFSKLVENGWKDITAEWVKGSAAPKELMEHLLNYGRIAEVTYKNNEDGYTDPEKYVGPQIASLYMEPIPL |
