# Supplementary material for: Genome sequencing of herb Tulsi (Ocimum tenuiflorum) unravels key genes behind its strong medicinal properties
Source: BMC Plant Biol. 2015 Aug 28;15:212. doi: 10.1186/s12870-015-0562-x (PMC4552454; doi:10.1186/s12870-015-0562-x)
Supplement: Additional file 24: — Text A. List of scaffolds as marked from START to END in Fig. 4. Text B. List of IDs of transcripts more abundant in Krishna as compared to Rama subtype (from top to bottom) marked in Fig. 5a. Text C. List of IDs of transcripts more abundant in Rama as compared to Krishna subtype (from top to bottom) as marked in Fig. 5b. Text D. Multiple sequence alignment of amyrin synthases with hits in Tulsi genome. [file 12870_2015_562_MOESM24_ESM.doc]

Supplementary Text A. List of scaffolds as marked from START to END in Figure 4.

Ote12751400

Ote12004500

Ote16262000

Ote11932400

Ote11150700

Ote14150000

Ote11217600

Ote12139400

Ote11381100

Ote11223300

Ote12451900

Ote12450600

Ote12359500

Ote16703000

Ote12331300

Ote11445300

Ote12197000

Ote11934000

Ote17341000

Ote19109000

Ote11564300

Ote11062100

Ote23765473

Ote12663900

Ote12498900

Ote12730800

Ote23766235

Ote11202700

Ote11809800

Ote23794941

Ote12581300

Ote12138500

Ote11311500

Ote12650000

Ote12773400

Ote15900000

Ote11323400

Ote12622800

Ote11059300

Ote11685900

Ote12659200

Ote12775400

Ote12167000

Ote12556400

Ote18821000

Ote12319400

Ote13513000

Ote11616200

Ote11545100

Ote23729009

Ote11648500

Ote17175000

Ote23795879

Ote12751700

Ote12723000

Ote11977300

Ote12123200

Ote12701800

Ote16372000

Ote11409000

Ote12446000

Ote19104000

Ote11633300

Ote12304300

Ote11227400

Ote15500000

Ote12280800

Ote23776143

Ote11815600

Ote11238800

Ote12782300

Ote11416900

Ote11874200

Ote12032000

Ote11231200

Ote12080100

Ote12555600

Ote11509700

Ote11082000

Ote11832900

Ote11225500

Ote12584900

Ote11813300

Ote12580000

Ote12485400

Ote23773517

Ote11669700

Ote15931000

Ote11618800

Ote23780913

Ote19391000

Ote12409600

Ote17256000

Ote13788000

Ote12138900

Ote12299200

Ote16880000

Ote16357000

Ote12705800

Ote12554900

Ote11272600

Ote11294000

Ote23786801

Ote23786497

Ote11566500

Ote12262800

Ote11609300

Ote11811800

Ote12545200

Ote13528000

Ote11162000

Ote11747600

Ote15968000

Ote12076200

Ote23787117

Ote12666600

Ote11348200

Ote13840000

Ote11558400

Ote11581700

Ote12572900

Ote12174300

Ote11736700

Ote12056300

Ote13367000

Ote12507200

Ote15183000

Ote15546000

Ote12457800

Ote11481800

Supplementary Text B. List of IDs of transcripts more abundant in Krishna as compared to Rama subtype (from top to bottom) marked in Figure 5A.

1) C210834_81.0 2) C205742_60.0 3) C176468_120.0 4) C270314_133.0

5) scaffold6254_Locus_25679_0_120.8_LINEAR 6) C289596_124.0 7) C280804_113.0

8) C122408_127.0 9) C152018_91.0 10) C219312_106.0 11) C56900_117.0

12) scaffold10010_Locus_52797_0_102.4_LINEAR 13) C111838_127.0

14) C193208_103.0 15) scaffold3801_Locus_12195_0_125.5_FORK

16) C211544_127.0 17) C214470_104.0 18) scaffold266_Locus_883_0_126.3_LINEAR

19) C204564_80.0 20) C247892_82.0

21) scaffold10537_Locus_56789_0_115.4_LINEAR

22) C279374_107.0 23) C255900_128.0 24) C290224_121.0 25) C277198_79.0

26) scaffold10740_Locus_58335_0_121.4_LINEAR 27) C287934_123.0

28) C288980_123.0 29) C288600_113.0 30) C287206_126.0 31) C184460_134.0

32) C286096_121.0 33) scaffold5776_Locus_22871_0_115.9_LINEAR 34) C261218_110.0

35) scaffold6680_Locus_28062_0_115.1_LINEAR 36) C225854_46.0

37) C264566_111.0 38) C205402_90.0 39) C287452_108.0 40) C194780_73.0

41) C285468_119.0 42) C244004_141.0

43) scaffold12545_Locus_71737_0_111.4_LINEAR

44) C244036_94.0 45) scaffold7632_Locus_34228_0_114.7_LINEAR 46) C287944_93.0

47) scaffold5532_Locus_21231_0_108.8_LINEAR 48) C265582_89.0

49) scaffold8571_Locus_41405_0_113.1_LINEAR 50) C282040_97.0

Supplementary Text C. List of IDs of transcripts more abundant in Rama as compared to Krishna subtype (from top to bottom) as marked in Figure 5B.

A) C192356_104.0 B) C61182_127.0 C) C207744_82.0 D) C52524_127.0

E) C195714_113.0 F) C236280_70.0 G) C152604_118.0 H) C201414_97.0

I) C187944_129.0 J) C228784_118.0 K) C190976_113.0 L) C220608_83.0

M) C182220_81.0 N) C214192_100.0 O) C236666_123.0 P) C103084_127.0

Q) C157514_162.0 R) C257514_122.0 S) C232008_119.0 T) C257584_104.0

U) C228598_156.0 V) C237388_122.0 W) C219110_29.0 X) C195708_79.0

Y) C247820_128.0 Z) C227632_104.0 AA) C239458_146.0 AB) C263858_116.0

AC) C186330_81.0 AD) C133604_119.0 AE) C229838_102.0 AF) C204152_155.0

AG) C226268_119.0 AH) C187910_109.0 AI) C176828_27.0 AJ) C277706_105.0

AK) C257934_130.0 AL) C250128_109.0 AM) C247984_123.0 AN) C228344_111.0

AO) C138096_159.0 AP) C105682_71.0 AQ) C88368_102.0 AR) C234110_104.0

AS) C215276_99.0 AT) C219698_123.0 AU) C257660_115.0 AV) C139304_117.0

AW) C218510_51.0 AX) C171252_108.0


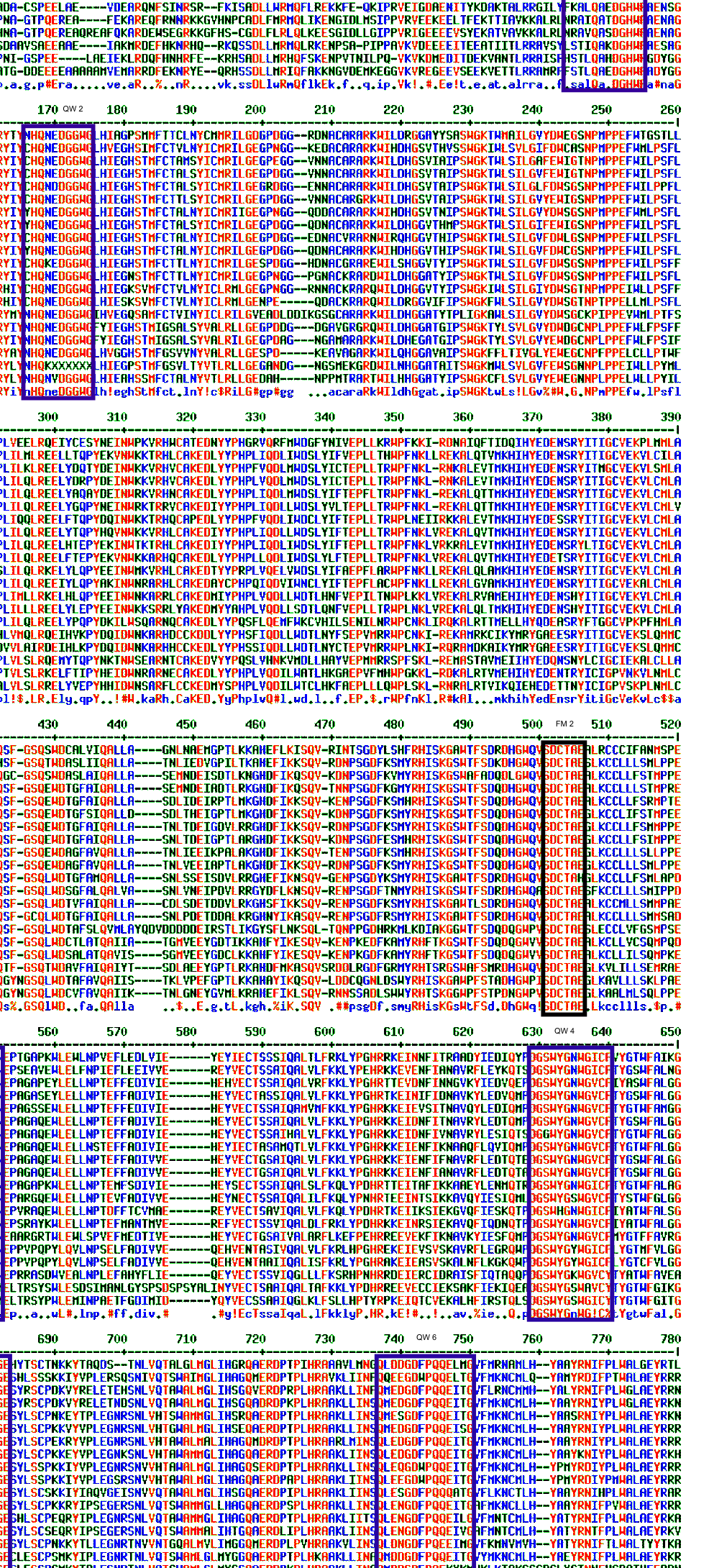


Supplementary Text D. Multiple sequence alignment of amyrin synthases with hits in Tulsi genome.

Important motifs marked on the alignment of amyrin synthases with *O. tenuiflorum* homologues.
